# A 21st Century Evil: Immunopathology and New Therapies of COVID-19

**DOI:** 10.3389/fimmu.2020.562264

**Published:** 2020-10-27

**Authors:** Taylon Felipe Silva, Fernanda Tomiotto-Pellissier, Raquel Arruda Sanfelice, Manoela Daiele Gonçalves, Bruna Taciane da Silva Bortoleti, Mariana Barbosa Detoni, Ana Carolina Jacob Rodrigues, Amanda Cristina Machado Carloto, Virgínia Márcia Concato, Elaine da Silva Siqueira, Idessania Nazareth Costa, Wander Rogério Pavanelli, Ivete Conchon-Costa, Milena Menegazzo Miranda-Sapla

**Affiliations:** ^1^ Laboratory of Immunoparasitology of Neglected Diseases and Cancer—LIDNC, Department of Pathological Sciences, Center of Biological Sciences, State University of Londrina, Londrina, Brazil; ^2^ Biosciences and Biotechnology Graduate Program, Carlos Chagas Institute (ICC), Fiocruz, Curitiba, Brazil; ^3^ Laboratory of Biotransformation and Phytochemistry, Department of Chemistry, Center of Exact Sciences, State University of Londrina, Londrina, Brazil

**Keywords:** Severe Acute Respiratory Syndrome Coronavirus 2, Coronavirus Disease 2019, immunopathology, cytokine storm, cellular exhaustion, treatment, coronavirus

## Abstract

Coronavirus Disease 2019 (COVID-19) has been classified as a global threat, affecting millions of people and killing thousands. It is caused by the SARS-CoV-2 virus, which emerged at the end of 2019 in Wuhan, China, quickly spreading worldwide. COVID-19 is a disease with symptoms that range from fever and breathing difficulty to acute respiratory distress and death, critically affecting older patients and people with previous comorbidities. SARS-CoV-2 uses the angiotensin-converting enzyme 2 (ACE2) receptor and mainly spreads through the respiratory tract, which it then uses to reach several organs. The immune system of infected patients has been demonstrated to suffer important alterations, such as lymphopenia, exhausted lymphocytes, excessive amounts of inflammatory monocytes and macrophages, especially in the lungs, and cytokine storms, which may contribute to its severity and difficulty of establishing an effective treatment. Even though no specific treatment is currently available, several studies have been investigating potential therapeutic strategies, including the use of previously approved drugs and immunotherapy. In this context, this review addresses the interaction between SARS-CoV-2 and the patient’s host immune system during infection, in addition to discussing the main immunopathological mechanisms involved in the development of the disease and potential new therapeutic approaches.

## Introduction

The ongoing outbreak of Coronavirus Disease 2019 (COVID-19) has been classified as a threat of international concern and a public health emergency, having affected almost 30 million people and killed more than 900,000 around the world so far, according to the World Health Organization (WHO) (1). The etiologic agent of this pandemic is Severe Acute Respiratory Syndrome Coronavirus 2 (SARS-CoV-2), the third coronavirus to have emerged as a public health issue and to cause an outbreak in the human population over the past two decades (2). This is the nomenclature referred to in this paper (3), which is derived from its similarity to the SARS-CoV virus that caused the outbreak in 2003, which is now known as “SARS-CoV-1”.

Coronaviruses belong to a group of single-stranded RNA viruses and are regarded as one of the main types of viruses that affect the human respiratory system. SARS-CoV-2 is the seventh coronavirus known to have infected humans; SARS-CoV-1, MERS-CoV, and SARS-CoV-2 can cause serious illness, whereas HKU1, NL63, OC43, and 229E are associated with mild symptoms (4).

SARS-CoV-2 emerged at the end of 2019 in Wuhan, China, with reports of infection in humans and quickly spread around the world. The virus causes COVID-19, which consists of a spectrum of clinical syndromes, ranging from fever and breathing difficulty to acute respiratory distress and death, critically affecting older patients and people with comorbidities, including heart disease, diabetes, and other health conditions (5).

SARS-CoV-2 infection can be subdivided into the following three general phases: the spread of the virus in the body – known as viremia –; the acute phase with the appearance of clinical signs; and the stage of convalescence, which progresses either to recovery or death (6).

The pathological mechanism, so far unraveled, has proposed the role of the host’s angiotensin-converting enzyme 2 (ACE2) and its affinity with viral receptors, especially the glycoprotein spike. The high affinity between these molecules facilitates viral dissemination in the body and allows the infectious condition to be established (7, 8). The immune systems of infected patients have demonstrated important changes, such as lower effector T cells, loss of the antiviral capacity of CD8^+^ T lymphocytes and natural killer cells (NK), and the excessive release of inflammatory mediators, which may contribute to the disease severity and difficulty in establishing an effective treatment (9, 10).

This is a highly transmissible virus, whose contagion usually occurs through droplets released by infected individuals when they cough, sneeze, or talk, directly contaminating other people by reaching mucous membranes on the face or contaminating the environment, later acting as a transmission source. Until now, we have relied on quarantine, social isolation, and infection-control measures to prevent disease spread, as well as supportive care for infected individuals. A specific antiviral agent to treat the infected individuals and decrease viral transmission (11, 12) is yet to be found. Several research groups around the world have been working on possible therapeutic strategies against SARS-CoV-2 by applying commercially available drugs, hoping to accelerate the discovery of an effective treatment (13, 14).

Since many studies are made available online on a daily basis, both in journals and in preprint servers, for this review we used only studies already published and peer-reviewed in order to avoid biased information. In this scenario, understanding the virus dynamics and host response is essential to formulate strategies for antiviral treatment, vaccination, and epidemiological control of COVID-19. Thus, our goal is to review SARS-CoV-2’s interaction with the patient’s host immune system during infection and discuss the main immunopathological mechanisms involved in COVID-19, as well as potential new therapeutical approaches.

## Coronavirus: An Overview

Coronaviruses (CoVs) consist of a group of enveloped, non-segmented, positive-sense single-stranded RNA viruses from the order Nidovirales, family Coronaviridae, and subfamily Orthocoronavirinae. Coronaviruses have the largest genome of all RNA viruses, encoding viral proteins involved in transcribing viral RNA, replication, structure, and accessory proteins (15). The virus has four main proteins – spike, envelope, membrane, and nucleocapsid (S, E, M, and N, respectively) – important for the virus to enter and replicate in the host cell (16), also representing the main molecules used for diagnosis, antiviral treatment, and potential vaccines.

According to antigenic and genetic criteria, CoVs are classified into three groups: α-CoVs, β-CoVs and γ-CoVs (17). Coronaviruses of human infection (hCoVs) are detected in both α-CoVs (hCoV-229E and NL63) and β-CoVs (MERS-CoV, hCoV-OC43, hCoV-HKU1, SARS-CoV-1, and SARS-CoV-2) (18). In addition to infecting humans, α-CoVs and β-CoVs can infect several species of mammals, including bats and pigs, while γ-CoVs infects birds, wild cats, pigs, and some species of marine mammals (19–22). CoVs have a high potential of jumping between species and their genome is characterized by high-frequency recombination and a high mutation rate (23).

hCoVs are responsible for the common cold and other respiratory pathologies with different degrees of severity, especially in babies, the elderly, and immunocompromised patients, characterized by human-to-human transmission (24). Coronavirus severe acute respiratory syndrome (SARS) and Middle East respiratory syndrome (MERS) (25–27) are caused by human β-CoVs and represent a serious illness with a case-fatality ratio of 9 - 10% and 35%, respectively (8, 28).

In contrast, according to data provided by the WHO, COVID-19 caused by SARS-CoV-2 shows an estimated lethality of ~5% of reported cases (data reported until July 2020) (29), reaching rates of up to 15% among elderly patients and patients with comorbidities. Despite the lower case-fatality rate, the high viral transmissibility of SARS-CoV-2 generates an overall number of cases that far outweighs SARS or MERS for spreading more easily among people (5, 28).

The first report of a COVID-19 case in Wuhan, China, occurred in December 2019, and in February the WHO declared the matter a public health emergency of international concern. Until now (September 2020), reports of COVID-19 account for almost 30 million cases and more than 900 thousand deaths in more than 220 countries, territories, or areas (1, 29). Imperial College, UK (30) proposed a mathematical model whose prospects indicate 7 billion infections and 40 million deaths in 2020 in the absence of mitigation measures.

Both SARS-CoV and MERS-CoV were initially believed to have resulted from a zoonotic spread from a bat population (31). α-CoVs and β-CoVs are believed to have evolved over thousands of years, restricted to bats and intermediate mammalian hosts (civet cats for SARS-CoV-1 and dromedary camels for MERS-CoV), which probably contributed to the zoonotic transmission of the new coronavirus to humans (32).

Regarding SARS-CoV-2 transmission, several works have demonstrated that coronaviruses found in pangolins (*Manis javanica*) and SARS-CoV-2 share a genomic similarity of approximately 91%. The presence of the virus in samples of pulmonary fibrosis in pangolins found around the COVID-19 outbreak suggests that these animals were the hosts responsible for spreading the virus among humans (4, 21, 33). In contrast, some researchers claim that SARS-CoV-2 did not come directly from pangolins since, despite their similarity, the viruses found in these animals do not have the essential tools needed to infect human cells (4, 34). Thus, the possibility of other animals, such as ferrets and snakes, acting as intermediate hosts for SARS-CoV-2 and being responsible for zoonotic transmission is still under consideration (35).

Since SARS-CoV-2 genomes’ information is still scarce and genomes of other coronaviruses closely related to this virus have limited availability (36), the evolutionary origin of SARS-CoV-2 is yet to be fully understood. So far, it is known that, compared with other β-CoVs, SARS-CoV-2 shows 50, 79, and 88 - 96% of genome similarity with MERS-CoV, SARS-CoV-1, and the bat SARS-like virus, respectively (37, 38).

The genomic changes of SARS-CoV-2 appear in both non-structural and structural proteins – notably in proteins S, M, and N – affecting viral multiplication, encapsulation, tropism, and transmission (39). Two important characteristics were described in the genome of SARS-CoV-2 that lead to alterations in the S protein: (i) receptor-binding domain (RBD), which is the most variable part of the viral genome, appears to be optimized for binding to the human ACE2 receptor, and (ii) presence of a polybasic (furin) cleavage site at the S1 and S2 boundary, *via* the insertion of twelve nucleotides, which allows effective cleavage by furin and other proteases and has a role in determining viral tropism, infectivity, and host range (4).

**Figure 1:**
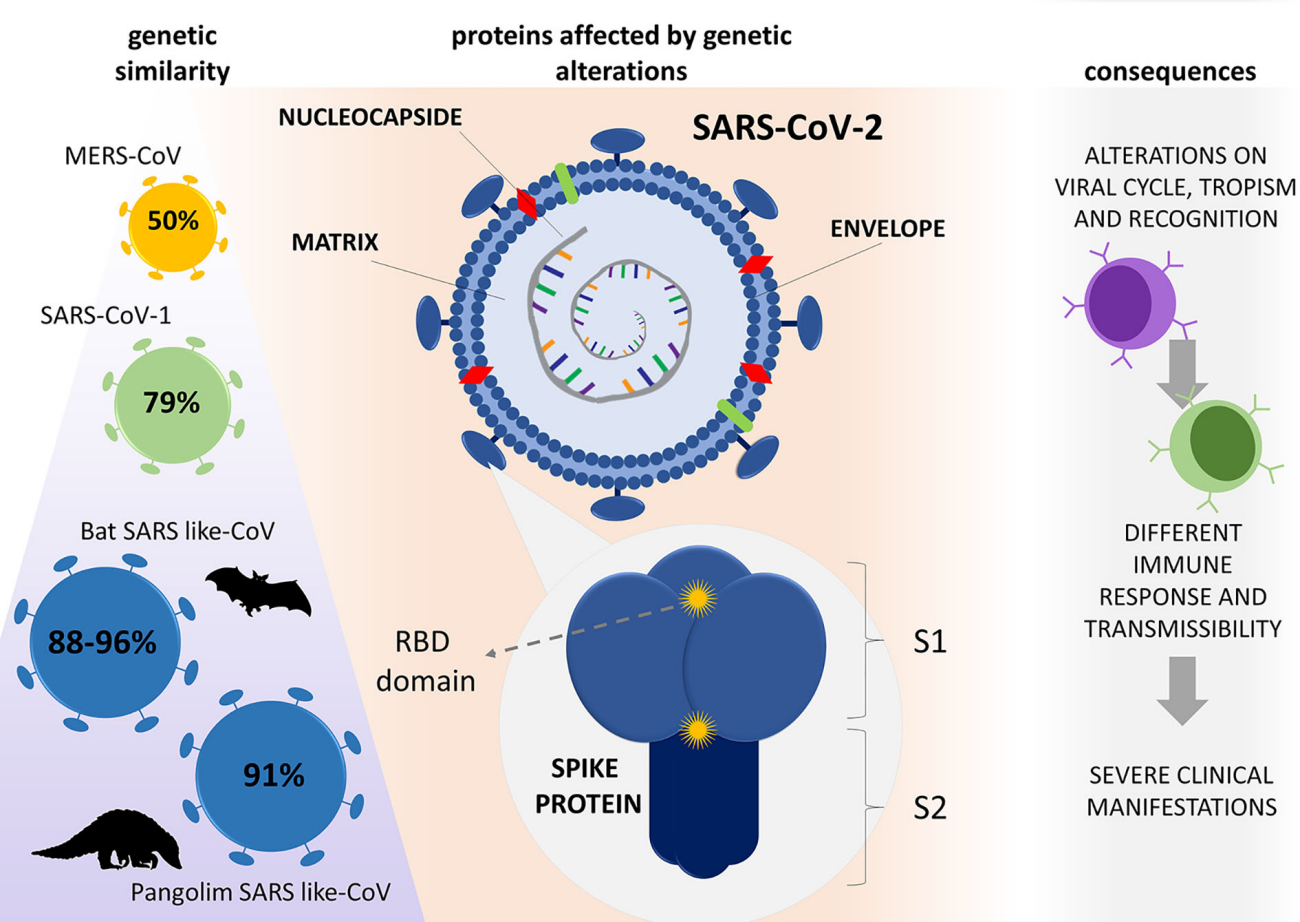
Genetic evolution of SARS-CoV-2 and its consequences. Compared with other β-CoVs, SARS-CoV-2 has similarities of 50, 79, and 88 - 96% to MERS-CoV, SARS-CoV-1, and bat SARS-like-CoV genome, respectively, with 91% similarity with SARS-like CoV found in pangolins. The virus resulted from mutations that caused changes in important proteins for its virulence; notably, the spike, matrix, envelope, and nucleocapsid proteins caused alterations in host cell interactions, which culminated in a new aggressive disease (COVID-19). RBD (receptor binding domain), S1 (subunit 1) S2 (subunit 2).

Such genomic changes also affected the recognition of these viruses by immune cells. Baruah and Bose (40) demonstrated that Sars-CoV-2 has specific regions for B cell and cytotoxic T cell glycoproteins recognition, which does not coincide with those found in bat-derived CoV, SARS-CoV-1, or MERS-CoV (**Figure 1**). Such distinguished interaction of cells and viruses can promote unusual immunomodulation or immune responses that contribute to the severity of the disease. All aspects of immunomodulation and immune evasion will be discussed in the subsequent topics.

## Pathogenesis of SARS-CoV-2

### Viral Entry and Replication

A virus starts its infection by binding viral particles to the host’s surface cellular receptors. The recognition of cellular receptors is the first step towards viral entry into host cells, in addition to determining their tropism. The ability to engage receptors and the affinity of binding can define the efficiency of a virus when infecting an organism, while the amount of these receptors present in cells can indicate the intensity of infection. Viruses that have a high capacity to bind to more conserved receptors are more likely to migrate between different species, which may also reflect the susceptibility of hosts and increase viral pathogenicity (40, 41).

As well as the other β-CoVs, the SARS-CoV-2 genome has a long open reading frame (ORF) 1ab region, followed by regions that encode S, E, M, and N proteins (42). Homotrimers of S proteins are present on the viral surface and are responsible for attaching to host receptors (43). The E protein plays a role in the assembly and release of the virus, in addition to being involved in viral pathogenesis (44). The M protein has three transmembrane domains and shapes the virions, promotes membrane curvature, and binds to the nucleocapsid (45, 46). Lastly, the N protein contains two domains that can bind to the RNA virus and is also an antagonist of interferon (IFN) and a virally-encoded repressor of RNA interference, which appears to benefit viral replication (47, 48).

The S protein of SARS-CoV-2 plays an important role in determining tropism for being able to activate receptors in host cells and induce the invasion process. This protein is cleaved by proteases into the S1 and S2 subunits, which are responsible for receptor recognition and membrane fusion, respectively (39). Several articles have experimentally demonstrated that the RDB in the S protein, especially in the S1 region, binds to the peptidase domain (PD) of the ACE2 receptor, which is part of the renin-angiotensin-aldosterone system, an enzyme present in the plasma membrane mainly of pulmonary, endothelial, cardiac, renal, and intestinal cells (7, 22, 38, 49, 50). The S2 subunit is known to contain the fusion peptide, in which it is inserted into the host cell membrane to trigger the fusogenic reaction (7, 51, 52). The interaction of the S glycoprotein with the CD26 receptor and CD209L (39, 53, 54) is also suggested, however, its role remains unclear.

The binding of the virus to the ACE2 receptor causes stabilization of the RBD in the standing-up state and triggers conformational changes in the S complex, resulting in the release of the S1 subunit and activation of S2 fusogenic activity (55). The S2 subunit contains an N-terminal fusion peptide (FP), heptad repeat 1 (HR1), heptad repeat 2 (HR2), a transmembrane region (TM), and a cytoplasmic tail (CT). During the fusion process, the FP portion is exposed and inserts into the membrane of the target cell, leading to a modification in S2, then the HR1 and HR2 come together to form a six-helical bundle (6-HR) structure, which allows the fusion between the membranes (55–57).

Therefore, CoVs need to elicit exogenous proteases to perform modifications of their binding receptors necessary for the connection to occur. SARS-CoV-2 has its own furin-like proteases, which play a role in these changes, providing it with an evolutionary advantage in relation to other coronaviruses and improving the process of cell infection and viral dissemination. Concerning exogenous proteins, SARS-CoV-2 can also use host proteins to prepare its S glycoprotein for receptor binding (49). Hoffman et al. (7) demonstrated *in vitro* that strains of the virus isolated from COVID-19 patients can use both the host protease transmembrane serine protease 2 (TMPRSS2) and cathepsins B/L to prime the S protein.

The entry mechanism of CoVs in host cells depends on the strain and species considered, as well as tissue and cell-type specificities (receptor/protease availability and local microenvironment) (58). After binding to a target host cell *via* interactions with cellular receptors, viral entry of CoVs can occur in two manners: (i) the endosomal pathway and (ii) the non-endosomal pathway (59, 60). The endosomal pathway is facilitated by low pH and pH-dependent endosomal cysteine protease cathepsins, helping to overcome the energetically unfavorable membrane fusion reaction and facilitating endosomal cell entry of CoVs (61, 62). The non-endosomal pathway is dependent on TMPRSS2, which allows the activation of the S protein for viral entry (63).

Once the viral genome is inside the host cell cytoplasm, translation of viral RNA produces RNA-dependent RNA polymerase (RdRp), which uses viral RNA as a template to generate virus-specific mRNAs (subgenomic mRNAs) from subgenomic negative-strand intermediates (64–66). Translation of subgenomic mRNAs leads to the production of structural and nonstructural viral proteins. Thus, after their formation, structural proteins are inserted into the membrane of the endoplasmic reticulum or Golgi, and viral particles germinate into the endoplasmic reticulum-Golgi intermediate compartment. Finally, the vesicles containing the virus particles fuse with the plasma membrane to release the virus (65, 67, 68).

Another possible mechanism for CoV entry may occur through antibodies. During the binding of the virus-antibody complex, simultaneous binding of viral proteins to antigen-binding fragment (Fab) regions of immunoglobulin G (IgG) and of the fragment crystallizable (Fc) portion of the antibody to Fc gamma receptors (FcγRs) that are expressed by immune cells occurs, promoting viral entry without the use of the ACE2 receptor (69, 70). However, the presence of viral RNA in the endosomes signals *via* the Toll-like 3 (TLR3), TLR7, or TLR8 receptor, activating the host cell to release pro-inflammatory cytokines that lead to exacerbated tissue damage, a phenomenon called antibody-dependent enhancement (ADE) (71).

Such a mechanism for SARS‐CoV‐2 is not yet fully understood, but previous coronavirus infections or SARS‐CoV‐2 convalescent patients with different SARS‐CoV‐2 strains could promote ADE, as experimentally shown for antibodies against the MERS‐CoV or SARS‐CoV-1 spike S protein (72). Several studies have shown that sera administration induced increased SARS-CoV-1 viral entry into cells that express the Fc receptor, and serum-dependent SARS-CoV-1 entry does not pass through the endosome pathway (73, 74).

This mechanism was characterized by Yip et al. (75) and Wang et al. (76), who revealed that the anti-Spike protein antibodies were in fact responsible for the infection of immune cells, and the enhancement of the infection can be improved by increasing the dilutions of antibodies. In relation to MERS-CoV, a similar mechanism has been demonstrated, since neutralizing monoclonal antibodies (nAb) are able to bind to the spike-S surface protein, allowing conformational changes and being subject to proteolytic activation. Meanwhile, nAb binds to the cell surface IgG Fc receptor, guiding viral entry through canonical pathways dependent on the viral receptor (77). Recent studies with COVID-19 patients reported that there was a strong IgG antibody response against the nucleocapsid protein and a delay in eliminating the virus, leading to an increase in the severity of the infection and contributing to the hypothesis of ADE of SARS-CoV-2 (78).

In view of this, the geographic discrepancy in pathogenesis can be explained, since individuals who have experienced previous exposure to coronaviruses are experiencing the effects of ADE due to the heterogeneity of the antigenic epitope (79). In addition, the potential of human antibodies for vaccination will depend on whether antibodies play a role in disease progression or in protecting against viral infection (70).

As an evasion mechanism, CoVs use a glycan conformational shield to prevent the recognition of the virus by the immune system, and, for this reason, S glycoproteins are found in trimers form and require structural alterations to engage with cellular receptors. In most of the hCoVs described, these S trimers are found in a naturally closed conformation, however, this mechanism also causes a delay in the process of cell infection due to the need for major changes in the glycoprotein conformation. It was described that, in SARS-CoV-2, the S trimers seem to exist in a partially open state, which prevents recognition by the immune system, but accelerates the initiation of conformational changes in the receptor and the processes of binding and fusion (49).

### Pathogenic Mechanisms

Considering the similarity between SARS-CoV-1 and SARS-CoV-2, it is likely that their biochemical interactions and pathogenesis are also similar (80, 81). Once SARS-CoV-2 was reported to use ACE2 to enter host cells, it is suggested that the virus may target a cell spectrum similar to SARS-CoV-1 (38, 82, 83). SARS-CoV-1 is known to mainly infect macrophages and pneumocytes in the lungs, as well as other extrapulmonary tissues that express ACE2, which can also be expected for SARS-CoV-2 (82–84). However, the affinity of SARS-CoV-2 to ACE2 is 10–20-fold higher than that of SARS-CoV-1, which could explain its higher transmissibility and demonstrate that it can bind more efficiently to host cells, having a robust infection in ACE2^+^ cells in the upper respiratory tract (7).

ACE2 is an enzyme belonging to the renin-angiotensin system, located on the cell surface of type II alveolar epithelial cells in the lungs and cells of other tissues, and plays a crucial role in controlling vasoactive effects in the body. Despite their similarities, ACE and ACE2 have different substrate specificities with distinct functionalities that perform opposite actions in the body. In brief, ACE cleaves angiotensin I to generate angiotensin II, the peptide that binds and activates angiotensin type 1 receptor (AT1R) to constrict blood vessels, thereby raising blood pressure. In contrast, ACE2 inactivates angiotensin II (Ang-II) while generating angiotensin 1-7 (Ang-1-7), a potent heptapeptide that acts in vasodilation and attenuation of inflammation (85).

Therefore, considering that SARS-CoV-2 uses ACE2 to enter cells, the main hypothesis of pulmonary pathology is that the increased activity of ACE (Ang-II) over ACE2 (Ang-1-7) may cause acute lung injury since the binding of the S protein to ACE2 leads to its blockage. Thus, the suppression of ACE2 occurs due to the increased internalization and release of ACE2 from the cell surface, which leads to a decrease in tissue ACE2 and the generation of Ang-1-7, and consequently higher Ang-II levels. Because of this, as shown in an experimental SARS-CoV-1 model, this process can drive an Ang II-AT1R-mediated inflammatory response in the lungs and potentially induce direct parenchymal injury (67, 80, 86, 87).

Another hypothesis states that SARS-CoV-2 infection blocks ACE2 function when binding to host cells, inhibiting its role of cleaving bradykinin and, as a consequence, bradykinin accumulates in the lung, promoting pulmonary edemas due to vasodilator activity and consequent respiratory failure. The increased bradykinin activation in the pulmonary endothelium can also induce neutrophil migration, enhancing tissue damage caused by the respiratory burst of these cells (88).

ACE2 is also highly expressed and co-expressed with TMPRSS2 in nasal epithelial cells, chalices, and hair cells (89). This finding is in accordance with the high detection of viral RNA in the upper airways present in nasal swabs and throats of both symptomatic and asymptomatic patients, demonstrating that the nasal epithelium is an important site for the infection to initiate and can represent an essential reservoir for viral dissemination and transmission (38).

Although the virus mainly affects the lungs, there are reports that SARS-CoV-2 also has organotropism, accompanied by dysfunction, in multiple organs, including the kidneys, liver, heart, and brain, which can influence the course of the disease and possibly worsen pre-existing conditions. It has been reported that ACE2, TMPRSS2, and cathepsin L can be expressed on glial cells and neurons, cardiomyocytes, liver cells, bile duct cells, and renal tubular cells (90, 91).

Evidence indicates that SARS-CoV-2 “neuroinvasion” can establish a direct entry along the olfactory nerve, mainly through the nasal olfactory epithelium, which expresses ACE2 and TMPRSS2, allowing access to the central nervous system (CNS). The spread of the virus through the hematogenous or transsynaptic pathway has also been widely discussed, however, it is known that the different levels of neurotropism and neurovirulence in patients with COVID-19 can be explained by a combination of viral factors and their interaction with the host (41, 92, 93).

Regarding the evolution of infected individuals, aging, comorbidities, and weakening of the immune system are factors that generally cause the infection to intensify at the acute phase, leading to the manifestation of more severe conditions (6). Thus, according to epidemiological studies, it is known that patients with chronic conditions, such as hypertension, diabetes, and chronic obstructive pulmonary disease (COPD), are more likely to develop a critical form of the disease (94–96).

The risk of applying medication commonly used in hypertension treatments to COVID-19 patients (97, 98) has raised different hypotheses over the issue of invoking a higher expression of ACE2 (99–101). A systematic review assessing the clinical outcomes for SARS-CoV-2-infected individuals regarding treatment using angiotensin-converting enzyme inhibitors (ACEIs) or angiotensin receptor blockers (ARBs) concluded that these types of drugs have no deleterious effects and should continue to be used in COVID-19 patients (102), reinforcing the recommendations of several medical societies, including the American Heart Association (103) and European Society of Cardiology (104).

Respiratory diseases, such as COPD and asthma, cause a reduced lung function and greater susceptibility to lung inflammation, and are expected to show a potentially critical course of COVID-19. COPD patients are already considered more susceptible to the development of pneumonia based on the clinical characteristics exhibited, such as lung structural damage, alterations in local/systemic inflammatory response, impaired host immunity, microbiome imbalance, persistent mucus production, and the presence of potentially pathogenic bacteria in the airways (105). Additionally, in the scenario of COVID-19, smokers and individuals with COPD have shown to have increased airway expressions of ACE-2 (106). It is still worth mentioning that patients who have this type of disorder often use corticosteroid immune-suppressing drugs, whose effect of reducing the immunity to respiratory infections may represent another contributing factor to a higher risk of infection (107).

### Clinical and Radiological Changes

Most COVID-19 patients exhibit mild to moderate symptoms, but approximately 15% progress to critical pneumonia and 5% eventually develop acute respiratory distress syndrome (ARDS), septic shock, multiple organ failure, and death (26, 108). Once the infection is installed, the spectrum of clinical presentations has been reported to range from asymptomatic infection to critical respiratory failure.

According to the severity of symptoms, patients can be classified as mild, severe, and critical. In general, the most commonly reported symptoms are fever, cough, myalgia, fatigue, pneumonia, dyspnea, as well as the loss of smell and taste, whereas less common reported symptoms include headache, diarrhea, hemoptysis, and a runny nose (108, 109). Most critically ill patients present progressive respiratory failure due to alveolar damage caused by hyper inflammation, which can result in lethal pneumonia (26).

A retrospective study conducted by Liu et al. (110) demonstrated that older patients with SARS-CoV-2 showed higher pneumonia severity index scores and had a higher chance of multiple lobe involvement compared with young patients. Elderly adults are more susceptible to SARS-CoV-2 and have a high risk of morbidity and mortality (111). This can be explained by factors such as physiological changes and multiple age-related comorbidities, in addition to associated polymedication (112).

Regarding the potential involvement of COVID-19 in the CNS, studies have investigated the neurological changes developed throughout the course of the disease. Nonspecific symptoms (dizziness, headache, and seizure) and specific symptoms (loss of smell or taste and stroke) were described (91, 113–115). Epidemiological studies have reported that some patients infected with SARS‐CoV‐2 did report headaches (8%), nausea, or vomiting (1%). A more recent study investigating 214 COVID‐19 patients found that about 88% of critically ill patients displayed neurologic manifestations, including acute cerebrovascular diseases and impaired consciousness (26, 116).

Among patients diagnosed with SARS-CoV-2, it has been reported that renal dysfunction is characterized by high levels of blood urea nitrogen, creatinine, uric acid, and D-dimer, associated with proteinuria and hematuria (90, 117–119). Recent studies have reported an incidence between 3-9% of acute kidney injury in COVID-19 patients, demonstrating renal abnormalities (94, 96, 111, 120). Cardiovascular complications are also associated with COVID-19 infection, including myocardial injury, myocarditis, acute myocardial infarction, heart failure, dysrhythmias, and venous thromboembolic events, being significant contributors to the mortality associated with this disease (121, 122).

Several studies found that CoVs can also affect other body regions, such as the gastrointestinal tract and ocular tissues (123, 124); some of them specifically investigated changes in the gastrointestinal tract and identified the presence of SARS-CoV-2 RNA in samples of anal/rectal swabs and feces of infected patients, establishing that the virus could be transmitted orally or fecally as well. Additionally, symptoms such as diarrhea, vomiting, and intestinal pain (125) have also been reported for SARS-CoV-2-positive patients, which can be associated with the expression of ACE2 in gastrointestinal epithelial cells, present especially in the small and large intestines, contributing to viral infection and replication in these cells (126).

Regarding ocular tissues, some studies have also identified the manifestation of conjunctivitis in patients with COVID-19 (<1%) (96), however, it is an underestimated number (127). Currently, it is still unclear how SARS-CoV-2 can cause conjunctivitis, but theories include: (i) conjunctiva can be a direct inoculation site for the virus, (ii) the virus can reach the upper respiratory tract through the nasolacrimal duct, or (iii) infection can occur *via* hematogenous through the lacrimal gland (123).

Histologically, biopsy samples of lungs reveal evident desquamation and hyaline membrane formation of pneumocytes, in addition to bilateral diffused alveolar damages along with cellular fibromyxoid exudate, indicating ARDS. In addition, the cytopathic effects found include multinucleated syncytial cells, increased atypical pneumocytes, and the presence of inflammatory infiltrates of mononuclear cells (26, 108).

More recently, reports on COVID-19 have included the occurrence of coagulation abnormalities in most critically ill patients (128–131). Tang et al. (132) reported the occurrence of disseminated intravascular coagulation in 71.4% of non-surviving COVID-19 patients and in only 0.6% of surviving patients, suggesting a high frequency in severe COVID-19 patients. Autopsies performed on patients with COVID-19 also demonstrated small fibrinous thrombi in pulmonary arterioles with endothelial tumefaction, the presence of megakaryocytes, and indications of coagulation cascade activation (133).

Although it is important to consider the direct procoagulant properties of SARS-CoV-2, the combination of immobility, systemic inflammation, platelet activation, endothelial dysfunction, and stasis of blood flow can lead to thrombotic complications that mimic systemic coagulopathies associated with severe infections, such as sepsis-induced coagulopathy (SIC), disseminated intravascular coagulation (DIC), and thrombotic microangiopathy (130). However, COVID-19 has some distinct features that may establish a new category of coagulopathy, denominated COVID-19 associated coagulopathy (CAC), whose main markers are higher D-dimer concentration and fibrinogen levels, a relatively lower platelet count, and longer prothrombin time (129). In COVID-19 patients, CAC has been associated with higher mortality (131).

Chest computed tomography (CT) in patients with COVID-19 has commonly demonstrated multifocal “*ground-glass*” opacity (GGO) in the lungs, which can occur concurrently with consolidation in posterior and peripheral areas, suggesting a pneumonia pattern in the organization of lung injury and indicating disease progression (134–136). Another important manifestation found through chest CTs is reticular pattern formation with interlobular septal thickening, which might be associated with interstitial lymphocyte infiltration and determine the disease course (108, 137, 138).

CT has highlighted many other alterations, including the “*crazy-paving*” pattern, which may result from the alveolar edema and interstitial inflammation in acute lung injury, and air bronchogram with a pattern of air-filled (low-attenuation) bronchi, but with gelatinous mucus and several airway changes, such as bronchiectasis and bronchial wall thickening resulting from the destruction of bronchial wall structure, proliferation of fibrous tissue, and fibrosis (137–140).

## Immune Response Against SARS-CoV-2

### Cytokine Storm

Antiviral immune response is usually coordinated by IFN-type cytokines that activate cells and intensify the response against these invading agents, triggered by the recognition of pathogen-associated molecular patterns (PAMPs) by pattern recognition receptors (PRRs), such as toll-like receptors (TLR), fundamental for pathogen recognition and activation of innate immunity. Type 7 of TLR (TLR7) – expressed on the surface of endosomes predominantly in the lungs, placenta, and spleen – might play a central role in COVID-19. This receptor has been reported to quickly recognize single-stranded SARS-CoV-1 RNA, inducing the production of pro-inflammatory cytokines such as TNF-α, IL-6, and IL-12 in plasmacytoid dendritic cells (141–143).

The recognition of SARS-CoV-2 RNA by TLR7 can mediate the release of cytokines in response to the virus, a context in which IL-6 may play an important role. It has been well described that IL-6 is a pleiotropic cytokine with distinct functions in different contexts in the immune system, being fundamental for the formation of follicular T helper lymphocytes and the generation of long-lived plasma cells. However, this cytokine can also inhibit the activity of CD8^+^ cytotoxic lymphocytes by inducing the expression of PD1 in these cells, in addition to inhibiting suppressors of cytokine signaling 3 (SOCS3), an important protein responsible for controlling cytokine production, leading to an excessive release of inflammatory mediators (144).

The pathophysiology of COVID-19 is yet to be fully elucidated and several gaps still need to be filled, however, several studies have shown an increase in cytokines, notably pro-inflammatory, in the serum of infected patients, which has been associated with hyper inflammation and the lung injury particular to the disease. The main cytokines described include TNF-α, IFN-γ, IL-1β, IL-1Ra, IL-2R, IL-6, IL-7, IL-8, IL-9, IL-10, basic FGF, G-CSF, GM-CSF, IP-10, MCP-1, MIP-1a, PD6F, and VEGF, in addition to an increase in other inflammation biomarkers, such as C-reactive protein, ferritin, and procalcitonin. However, mediators related to the complement system, such as C3 and C4, did not present any difference in healthy individuals. Furthermore, even higher levels of these mediators were found in patients of critical COVID-19 cases, suggesting that the severity of the disease may be associated with this huge amount of inflammatory mediators, called cytokine storm, which overloads the immune system with information, preventing the establishment of an effective immune response (26). For example, a study published by Valle et al. showed that COVID-19 patients have higher levels of IL-6, IL-8, and TNF-alpha than healthy individuals on hospital admission; moreover, when they stratified the population by low versus high cytokine levels and applied a risk competition model, it was found that each cytokine is an independent predictive factor of the patients’ overall survival and is significantly associated with worse clinical outcomes (145).

On the other hand, in theory, a type I IFN-mediated response activates the JAK-STAT signaling pathway that should be able to suppress viral replication and prevent the virus from spreading early in the infection. This is probably what occurs in asymptomatic individuals who can establish an effective response against SARS-CoV-2 (9, 141). However, in several viruses, viral proteins can modulate the production of this type of IFN, impairing the generation of an effective antiviral response (141, 143, 146, 147). Li et al. (148) conducted an *in vitro* experiment that revealed a strong capacity of ORF6, ORF8, and nucleocapsid proteins of SARS-CoV-2 to inhibit IFN-β and NF-κB activity, in addition to genes containing interferon-stimulated response elements (ISREs), suggesting that the virus has an important IFN antagonist activity.

By monitoring the production of type I IFN in SARS-CoV-2-positive patients, Trouillet-Assant et al. (149) found a peak in IFN-α2 production between 8 and 10 days after the onset of symptoms, in general, which reduces overtime. However, as many as one critically ill patient in five was unable to produce any amount of type I IFN and had a higher viral load, respiratory failure, and worse clinical outcome. Nonetheless, Zhou et al. (150) conducted a study that demonstrated that SARS-CoV-2 infection induced a markedly elevated expression of IFN-related inflammatory genes, which appears to decrease over time in mild cases, but not in severe ones.

Additionally, Major et al. (151) described the role of types I and III of IFN in lung repair during viral infection. The production of IFN-α/β and IFN-λ in C57BL/6 mice was detected immediately at the early stage of influenza virus infection and decreased over time, having reached undetectable levels at the onset of epithelial recovery. Interestingly, the treatment with IFN-α, β, or λ during the recovery phase reduced the proliferation type II alveolar epithelial cells by activation of IFN-induced p53, aggravating lung injury, disease severity, and susceptibility to coinfections. Therefore, time and duration of IFN are critical factors for viral infection response and should be thoroughly considered as a COVID-19 therapeutic strategy.

Similarly, an experimental study conducted with MERS patients indicated that type I IFN has protective activity against this infection and the blockade of its signaling resulted in delayed virus clearance, enhanced neutrophil infiltration, and impaired MERS-CoV–specific T cell responses. Additionally, early treatment using this type of IFN prevented fatal infections in mice. However, the late treatment did not cure the animals and failed to effectively inhibit virus replication, increased infiltration, and activation of monocytes, macrophages, and neutrophils in the lungs, in addition to having enhanced proinflammatory cytokine expression, which led to fatal pneumonia, indicating that type I IFN plays a central role at the very beginning of the infection (152).

Therefore, using IFN in SARS-CoV-2 treatment seems to be beneficial at the early infection stage, especially for patients unable to produce this type of response. Furthermore, as the disease progresses, the use of inflammatory cytokine blockers for patients who fail at regulating their production over time could represent a better strategy.

COVID-19 patients also have high levels of production of anti-inflammatory cytokines, such as IL-10, perhaps as a way of compensating for the exacerbated inflammatory response, which can lead to a picture of immune dissonance and anergy towards the infection (26, 153–155). It is fundamental to perform further studies that elucidate the mechanisms of the immune response and the balance between pro-inflammatory and anti-inflammatory response patterns to understand the immunopathogenesis of COVID-19.

IL-7 is a pleiotropic cytokine that plays an essential role in the differentiation and clonal expansion of lymphocytes. Chi et al. (156) described the production of IL-7 in COVID-19 patients; when compared to healthy controls, both asymptomatic and symptomatic individuals in the acute phase show an increase in the levels of this cytokine, however convalescent individuals return to the basal state equal to that observed in healthy individuals. When symptomatic individuals were stratified according to the severity of the disease, those with moderate to severe conditions had higher levels of IL-7. In addition, SARS-CoV-2-specific T cells from the peripheral blood of convalescent individuals of COVID-19 show high expressions of CD127, a receptor necessary for homeostatic cell proliferation triggered by IL-7, which may be related to the recovery observed (157). Patients with a severe COVID-19 condition, on the other hand, have an increased IL-7 production, but contradictorily they also have severe lymphopenia. Thus, we speculate that the deficiency in the expression of CD127 might occur in severely ill patients, which culminates in the deficiency of cell proliferation induced by IL-7 and consequent lymphopenia. However, studies that seek to evaluate the expression profile of IL-7 and CD127 in COVID-19 patients need to be carried out. In addition, the use of IL-7 as a treatment for COVID-19 patients has been evaluated and will be discussed further.

Several pro-inflammatory cytokines have been described in COVID-19 patients and are associated with the disease’s immunopathogenesis. Among them, IL-1β and TNF-α stand out for playing a central role in this context (26, 156). The respiratory failure characteristic of SARS-CoV-2 infection, especially in individuals who develop the most severe forms of the disease, occurs independently of infection or viral replication in the epithelial bronchial cells and probably occurs due to exacerbated inflammatory dysregulation, resulting from activation of the NLRP3 inflammasome pathway and consequent release of IL-1β (158). However, although several articles have shown an increase in IL-1β production in COVID-19 patients and early treatment with IL-1 receptor blockers has helped prevent respiratory failure (159), its exact role in the immunopathogenesis of the disease has not yet been fully described.

Cytokine storms may have great relevance in the pathogenesis of COVID-19. The induction of inflammatory mediators can induce cell damage, especially in lung tissues, causing respiratory failure. In addition, several of these mediators have potent vasodilator activity, which at the local level can cause pulmonary edemas, while at the systemic level leads to septic shock, worsening the clinical condition of these individuals. Similarly, several studies have shown that viral infections can induce cytokine storms, or take advantage of it, to establish infection and escape from the immune system, intensifying pathological phenomena such as those observed in sepsis, in addition to increasing the mortality rate of this population (160, 161).

Despite the absence of direct evidence of the role of cytokines and chemokines in lung injury, initial studies have shown that the increase in these pro-inflammatory mediators is associated with lung injury in patients with COVID-19 and has a central role in the pathogenesis of the disease (153). The balance of the innate immune response is essential at the beginning of the infection, while its imbalance can culminate in excessive inflammation, which hinders the establishment of an effective immune response against the virus.

Therefore, using hemoperfusion can be an important tool to treat severe COVID-19 patients who developed cytokine storms, as well as other treatments focusing on controlling and reducing hyper inflammation using specific blockers or monoclonal antibodies directed against the mediator or to antagonize its receptor (144).

### Innate Immune Response

The innate immune system is the first line of defense against pathogens through the activation of PRR in macrophages, neutrophils, and dendritic cells by the interaction with PAMPs. An effective innate immune response against viruses like SARS-CoV-2 is essential not only to initiate the response but also to structure the basis for the production of a robust and more specific adaptive response (162). Changes in this process, commonly observed in viral infections, can cause an immune imbalance and susceptibility of the host (163).

Patients who develop severe COVID-19 exhibited a marked increase in neutrophil and reduced lymphocytes counts compared with patients with mild signs of the disease (10). A general increase in the number of circulating neutrophils and the reduction of lymphocytes enhance the neutrophil/lymphocyte ratio, which has been used as a predictor of the infection severity and development of pneumonia. In addition to being a predictor of a worse prognosis, an increase in this ratio also indicates a serious immune imbalance in these patients (153).

In addition to having high levels of cell-free DNA, myeloperoxidase-DNA, and citrullinated histone H3 – important markers of neutrophil extracellular traps (NETs) –, the serum of COVID-19-positive patients was able to strongly trigger NETosis in healthy neutrophils *in vitro* (164). Despite representing important strategies to eliminate pathogens by neutrophils, NETs damage healthy tissue and induce inflammation (165), in addition to featuring a variety of oxidizing agents and being involved in several vascular diseases, as well as pathogen-induced acute lung injury. The release of NETs by neutrophils can be triggered by several factors, such as virus-damaged epithelial cells, activated platelets, activated endothelial cells, and inflammatory cytokines, such as IL-1β, IL-8, and G-CSF, among others (95, 166–169). In this context, it is fundamental to conduct studies assessing the role of neutrophils and NETs to better understand COVID-19 pathogenesis.

Concerning monocytes, COVID-19 patients have shown an abundant circulation of CD14^+^ CD16^+^ cells, with a sharper increase in patients who developed severe respiratory syndrome. This subtype of monocytes can over-secrete TNF-α, IL-1β, and IL-6 and expand quickly in systemic infections, implying that they must play an important role in the rapid defense against pathogens. Controversially, these cells are the main producers of IL-10, which makes their exact function in immune responses elusive (170, 171). Additionally, Dutertre et al. (172) demonstrated that CD14^+^ CD16^+^ monocytes are responsible for TNF overproduction in HIV infections and might be considered the major actor in immune hyperactivation in disease (172).

A study assessing bronchoalveolar lavage of SARS-CoV-2-positive individuals found an abundance of monocytes-derived inflammatory macrophages in critically ill patients. In addition, the authors observed through single-cell analysis that these macrophages can contribute to local inflammation by recruiting inflammatory monocytes and neutrophils through CCR1 and CXCR2 chemokine receptors. However, in patients who presented a moderate form of the disease, macrophages produced chemo-attractants for the recruitment of T cells, such as CXCR3 and CXCR6. Such a difference in response might be the key to understanding the pathogenesis of respiratory failure in COVID-19 (173).

Furthermore, critically ill patients have also manifested rapid proliferation of another subpopulation of monocytes characterized by GM-CSF^+^ IL-6^+^, which may be related to inflammatory risk and impairment of the lungs when migrating in large quantities (170). GM-CSF has been described as an active part of the pathogenesis of autoimmune and inflammatory diseases, mainly in the involvement of myeloid cells, such as monocytes, which can initiate tissue damage in a dependent manner on this marker (174, 175). In addition, high levels of mediators, such as IL-6, TNF-, and IL-10, found in these patients are likely to have been produced by these monocytes and to be highly involved in cytokine storm and pathogenesis of SARS-CoV-2, since as the disease progresses these mediators reduce, which is correlated to the restoration of the immune function of CD4^+^ and CD8^+^ T lymphocytes, which is further discussed later (154).

Critical COVID-19 patients have shown excessive activation of circulating HLA-DR^-^ monocytes, which has been associated with the onset of respiratory failure, suggesting its role as a predictive factor. The lack of expression of HLA-DR in monocytes may indicate a modulatory capacity of the virus, which prevents the antigen presentation and hampers the formation of an adaptive immune response (176, 177).

During an *in vitro* experiment, Yang et al. (178) found that, despite being permissive to infection by SARS-CoV-2, human monocyte-derived macrophages and dendritic cells are not able to effectively produce viral replicates. Despite their central role in pathogenesis, this may indicate that these cells are not important reservoirs for viruses. In addition, neither of the cell types developed a response based on type II IFN, but macrophages had lower production of type I and III IFN than the control, indicating that the virus can inhibit a response mediated by these types of IFNs. Additionally, macrophages were able to trigger an exacerbated inflammatory response with higher TNF-α, IL-8, IP10, MIP1α, and IL-1β. Dendritic cells had not been reported to show such inflammatory phenomenon, which is due to the ability of SARS-CoV-2 to inhibit STAT1 phosphorylation. Such important attenuation of dendritic cell response caused by the virus may have important implications for humans to develop effective immunity, therefore, further studies should seek to better elucidate such a relevant relationship.

Similarly, in the presence of IFN-α and GM-CSF, circulating monocytes should quickly differentiate into monocyte-derived dendritic cells (mDC), which are important antigen-presenting cells capable of phagocyting viruses and initiating the adaptive immune response process, as well as activating CD4^+^ T cells, generating immune memory in the process, and refining the body’s defense against infections (179, 180).

The number of mDCs has not increased in patients infected with SARS-CoV-2 compared with healthy controls, even in the most severe cases of the disease. Interestingly, the levels of GM-CSF in the serum of these patients are highly elevated, which should lead these cells to increase, demonstrating that the virus may have a mechanism to control the production of IFN-α and consequent differentiation of mDCs (9, 26). In the same way, individuals infected and not infected with SARS-CoV-2 have similar levels of IL-12, an important cytokine produced by mDC that is involved in the differentiation of naïve T cells (9, 181).

Thus, we hypothesize that the lack of mDC generation and consequent inability of the infected individual to produce IL-12 may be among the main factors of innate immunity-related pathogenesis of COVID-19. As further discussed, the increase in naïve T cells, reduced cell functionality of CD4^+^, CD8^+^, and natural killers (NK), and delay in the appearance of humoral response found in these patients indicates a failure in the generation and function of mDC. Therefore, it is urgent to carry out studies aimed at analyzing the effect of SARS-CoV-2 on dendritic cells.

### T Helper Cells

Establishing and maintaining immune response and memory generation against viruses depends on the activity of T cells. These lymphocytes originate from bone marrow progenitor cells and migrate to the thymus for maturation, selection, and peripheral export. Peripheral T cells are subdivided into groups that include naïve T cells, which are capable of responding to new antigens, memory T cells derived from previous antigen activations and maintain long-term immunity, and regulatory T cells that coordinate the immune response (163).

The immune response begins when naïve T cells encounter antigens and co-stimulatory molecules presented by antigen-presenting cells, such as dendritic cells that phagocytize the virus, resulting in the production of IL-2, proliferation, and differentiation of effector T cells, which migrate to various sites to promote the elimination of pathogens (163, 182).

Inflammatory factors induced by viruses can trigger a storm of mediators that cause changes in the differentiation and activation of T cells, disturbing the homeostasis of the immune system. In patients with COVID-19, the overall percentage of T lymphocytes is generally reduced, especially CD4^+^ CD3^+^ T lymphocytes, which have an activation phenotype, a reduction much more pronounced in severely ill patients. Furthermore, a higher percentage of CD4^+^ CD45RA^+^ naïve cells and lower CD4^+^ CD3^+^ CD45RO^+^ memory T cells were also found in COVID-19 patients (10, 153, 183, 184).

Polyfunctional CD4^+^ T cells are characterized by the expression of activation markers as well as their capacity to produce IFN-γ, IL-2, and TNF-α. These cells have been linked to an excellent response against viral infections and during the development of immunity by vaccination (185, 186). Even though COVID-19 patients have shown an increase in the expression of molecules related to T CD4^+^ activation, such as CD69, CD38, and CD44, molecules related to their function, such as intracellular IFN-γ, IL-2, and TNF-α, are reduced, especially in individuals with a more severe stage of the disease (9, 170), indicating an impairment of polyfunctional T cells.

Li et al. (187) demonstrated that patients infected with SARS-CoV-1 had elevated levels of polyfunctional CD4^+^ T cells, especially those in a severe condition but who progressed to clinical improvement. In contrast, critically ill patients with SARS-CoV-2 demonstrated a drastic reduction of this cell subtype, which may indicate that this virus has developed its own mechanisms to control cellular responses, thus differing from other coronaviruses (9).

Similarly, Chen et al. (154) demonstrated that CD4^+^ T lymphocytes from COVID-19 patients showed increased expressions of T cell immunoglobulin-3 (Tim-3), a type I transmembrane protein that acts as a negative regulator of Th1 pattern. CD4^+^ cells showed low expression of this marker at the early phase of infection, having progressively increased over time, indicating that the exhaustion of these cells occurs as the disease progresses.

Many studies with SARS-CoV-2 positive patients have described the generation of these exhausted pathological lymphocytes that exacerbate the inflammatory response at the early stage of infection, initiating a cytokine storm, followed by cell exhaustion and loss of functionality, a phenomenon that has appeared mainly in more severe cases of the disease (9, 10, 170, 188).

The vast majority of studies to date show impairment of proliferation, maturation, and response of T cells, especially in sicker patients. This may indicate that SARS-CoV-2, similarly to other viral infections, can interfere with the function of CD4^+^ cells at the very beginning of the infection, causing excessive release of inflammatory mediators and exhaustion of the response capacity of these cells over time, reducing the host’s antiviral immunity (189). What seems to happen in COVID-19 is that the total lymphocyte count is reduced in these patients and, among the remaining T cells, the highest percentage is from naïve CD4^+^ T lymphocytes, while the activated subpopulations, although few, present a phenotype with excess and reduced markers related to activation and function, respectively, indicating that, despite overactivation, these cells fail to exercise effective immune activity.

Another important point is the reduction of regulatory T cells CD4^+^ CD25^+^ Foxp3^+^ verified in these patients. These cells have a fundamental role in the negative regulation of inflammation, control of cell proliferation, and the effector function of several cells, which probably has contributed to the excessive inflammation observed in critically ill patients (153, 190). It has been described that regulatory T cells play a central role in mitigating the immune response in several viral infections (191); reducing the number of these cells in patients with COVID-19 can lead to loss of regulatory functions and consequent cytokine storm.

It has been described in several studies that COVID-19 patients have a reduced number of circulating Treg cells, which may be due to the increase in soluble IL-2 receptors (IL-2R or CD25) that potentially scavenges IL-2, reducing their bioavailability for binding to CD25 on the cell surface, thus preventing the induction of the clonal expansion signal of Treg cells (153, 192, 193).

### Cytotoxic Cells

T lymphocytes CD8^+^ and NK are essential to control viral infections due to their cytotoxic effect. These cells become activated after recognizing antigens attached to molecules of MHC-I presented by infected cells, which usually leads to the death of the infected cell by effector mechanisms (163).

Kamiya et al. (194) demonstrated that SARS-CoV-2 infection in humans dramatically reduces the total CD8^+^ and NK cell count, especially in patients who have developed more severe disease. The inhibition of these cells was characterized by an increase in the expression of NK inhibitory receptor CD94/NK group 2 member A (NKG2A), a type C lectin receptor of cytotoxic cells that acts as a potent suppressor when binding to minimally polymorphic MHC-I that present peptide sequences of other MHC-I molecules, inducing an inhibitory signal through two receptors with tyrosine-based inhibition motifs that suppress cytokine secretion and cytotoxic activity.

In patients who recovered from COVID-19, CD8^+^ and NK cell counts and the reduction in NKG2A expression were restored, suggesting that the inhibition of these cells is a result of SARS-CoV-2-mediated immunomodulation (10). Corroborating these data, it has been reported that other viral infections also manage to increase the expression of NKG2A in NK cells as a way to escape from the immune system (195).

SARS-CoV-2 studies involving CD8^+^ T cells have shown exhaustion of the effector capacity of these cells over time by the reduction of granzyme B, perforins, and lysosome-associated membrane protein 1, also known as LAMP-1 or CD107a, described as a marker of cytotoxic cells’ degranulation and an important parameter to assess the activity of these cells. CD8^+^ T cells from COVID-19 patients have a very marked activation phenotype with an increase in CD69, CD38, CD137, and CD44, especially in critically ill patients. However, despite presenting an increase in these activation molecules, these cells also have enhanced cell exhaustion proteins, such as PD1, Tim3, CTLA-4, and TIGIT, especially in more critically ill patients (9, 154, 170).

TIGIT receptor, present in T and NK cells, can bind to dendritic cell CD155 receptors and induce an increased expression of IL-10 and reduce IL-12, in addition to inhibiting T cell activation and blocking cytotoxicity of NK cells (196). The use of specific blockers for these receptors, such as anti-PD1 and anti-TIGIT, has helped in the recovery of the function of these cells. Therefore, it is logical to assume that specific NKG2A blockers could be an important tool to assist in the treatment of SARS-CoV-2 infections, restoring the functionality of cytotoxic cells (10, 195).

**Figure 2:**
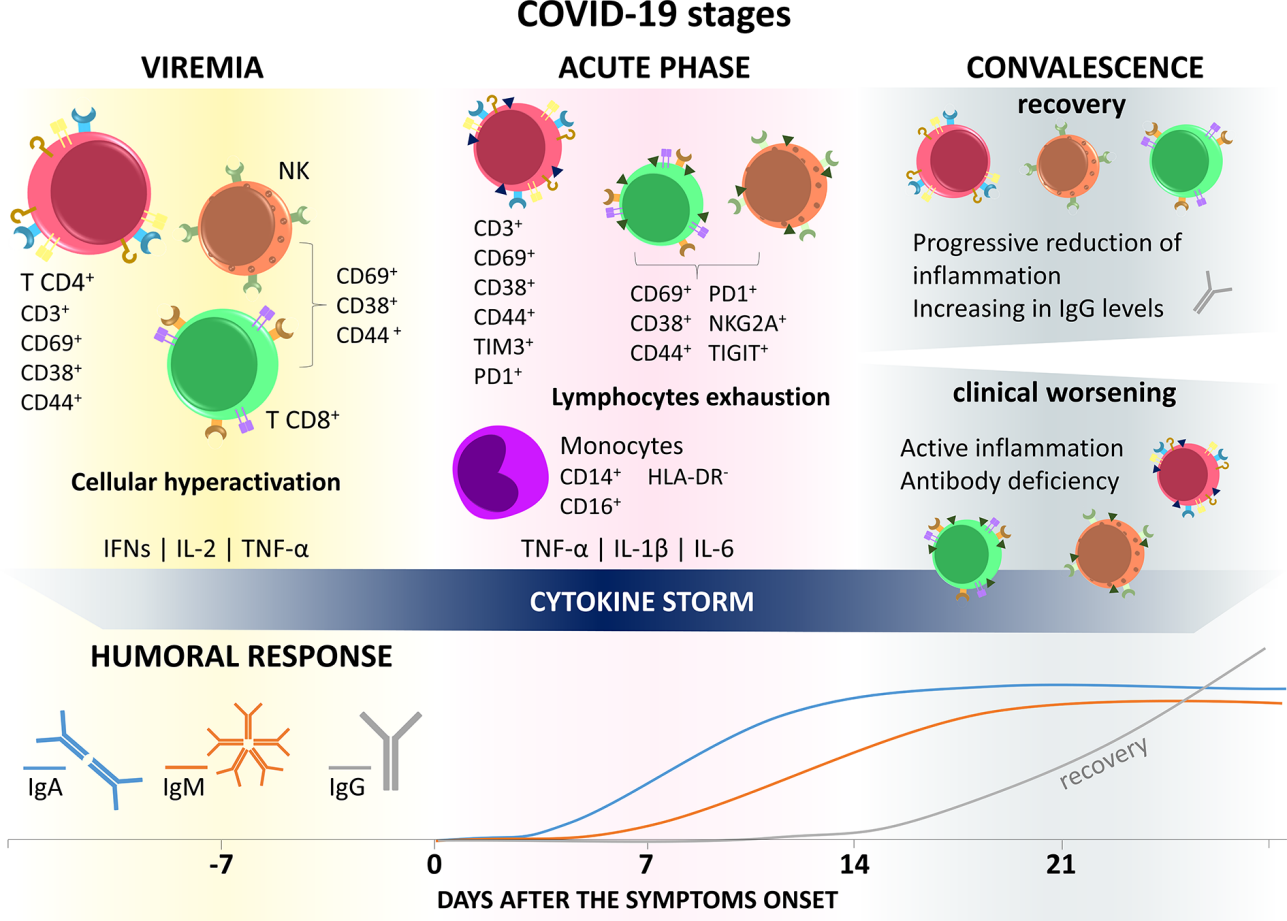
Immune response in COVID-19 stages. SARS-CoV-2 infection is divided into three general phases. In the first one, called viremia, the virus spreads through the body and there is excessive activation of immune cells with exacerbated production of inflammatory mediators, such as IFN-γ, IL-2, and TNF-α, triggering cytokine storms and immune impairment. The second (acute) phase, characterized by the appearance of COVID-19 symptoms, presents a profile of immune cells still hyperactivated, but with the presence of cell exhaustion markers, such as Tim3, PD1, TIGIT, and NKG2A, in addition to losing the functional capacity of producign IFN, IL-2, and TNF-α. In this period there is still the appearance of CD14^+^ CD16^+^ hyperinflammatory monocytes, with a high production capacity of TNF-α, IL-1β, and IL-6, which will migrate to the lungs, contributing to the pathogenesis of respiratory failure and maintaining the cytokine storm. The lethargic state of the immune system in the early stages of infection may be related to the delay in the generation of a humoral response. In the third, or convalescence, phase, the individual can evolve in two opposite directions, recovery or clinical worsening/death. In recovery, cells of lymphoid origin recover their effector function and lose markers of exhaustion, while IgG levels improve. On the other hand, in patients with clinical worsening, this status of immune anergy continues.

Together, these data described the increase in activation markers and cellular exhaustion, in addition to the reduction in functionality markers indicating that, like CD4^+^ T lymphocytes, these cells were probably hyperactivated right at the beginning of the infection, collaborating with the generation of a cytokine storm, until they became exhausted and lost their functional capacity, causing reduction of antigen-specific response and loss of its antiviral effects (**Figure 2**).

### Humoral Response

Detecting a humoral response against SARS-CoV-2 has been the focus of attention to developing faster and more accurate diagnostic tools. A study assessing the presence of IgA, IgM, and IgG against SARS-CoV-2 in infected patients found that IgA levels begin to rise in the first seven days after the onset of symptoms and continue increasing until it stabilizes near the 14^th^ day after the onset of symptoms. Additionally, IgM production appears as early as IgA, the antibody titration in the first seven days after the onset of symptoms was very low, starting to increase only from the eighth day, reaching a plateau after the 14^th^ day. The average time of appearance of specific IgG against SARS-CoV-2 starts 14 days after the appearance of symptoms and grows exponentially until the 21^st^ day (197).

These data corroborate the disease stages proposed by Lin et al. (6) (presented in the introduction), and the changes in the immune response present in the disease. The early appearance of IgA results from the first contact of the virus with the individual’s mucosa at the moment of contagion and continues to increase until the acute phase. Despite viremia and symptom onset, IgM levels only begin to rise from the eighth day after symptom onset, indicating anergy in the immune response during this period, perhaps caused by dysfunction in antigen-presenting cells, such as dendritic cells, and also by reducing the amount of activated T helper cells, which play a central role in triggering the immunity acquired by the activation and clonal expansion of B lymphocytes, in addition to the formation of germinal centers and generation of plasma cells that produce high affinity and avidity antibodies (198, 199).

The appearance of IgG near the third stage of the disease may be related to the clinical evolution of patients and those who fail to establish an efficient immune response might be at risk of death. Interestingly, Guo et al. (197) found that approximately 22% of COVID-19 patients confirmed by RTq-PCR did not present detectable levels of IgM. Most of these individuals were tested in the first seven days after the onset of symptoms, therefore the lack of IgM can be justified by the delay in generating a humoral response against SARS-CoV-2. However, some critically ill patients followed for a longer period remained negative for IgM even 22 days after the onset of symptoms. As for IgG levels, some patients took 30 to 40 days after the appearance of symptoms to show some detectable level of IgG, suggesting a possible failure in the production of antibodies that may have contributed to the severity of the disease. It is possible that the generation of antibodies in more advanced stages of COVID-19 does not benefit the recovery process since most pathological mechanisms at this stage might be more related to the excess of inflammatory mediators than the presence of the virus itself.

In recovered patients, the magnitude of the production of neutralizing antibodies (nAb) against SARS-CoV-2 is positively correlated with the severity of the disease; while asymptomatic individuals have little or no capacity to produce nAb, individuals who recovered from severe cases of COVID-19 had robust nAb production. Also, these severe recovered patients showed an increase in B cell receptor (BCR) rearrangement, which may demonstrate that the effective production of nAb requires enhanced and prolonged BCR stimulation. Asymptomatic or mild symptomatic patients may possibly mount robust SARS-CoV-2 specific CD8 + T cell responses, which can provide protection by directly eliminating the target cells infected by the virus. However, due to the lack of immunity provided by nAb, these individuals might suffer from SARS-CoV-2 reinfection (200, 201).

In the same way, Zhang et al. (202) also demonstrated that patients who recovered from severe COVID-19 have high levels of BCR clonal expansion and B cell activation, indicating a more robust humoral response than patients with mild disease, thus asymptomatic individuals or those with mild COVID-19 probably have different cell and humoral responses than individuals who developed the severe form of the disease.

In an article published by Chen et al, the serum of 26 patients who recovered from COVID-19 were analyzed for the production of IgG anti-SARS-CoV-2 S1 protein antibodies. It was found that, despite the majority of patients presenting high IgG titers, only three individuals had antibodies that effectively neutralized the binding of the viral glycoprotein to the human ACE2 receptor. In addition, the authors successfully managed to clone two different neutralizing antibodies from these patients with the ability to inhibit virus-cell binding, opening up the potential for using them as a possible source of treatment for COVID-19 (203).

In theory, the production of specific neutralizing antibodies against SARS-CoV-2 should be able to combat the virus and reduce viral load. The production of immune memory verified in the blood of recovered patients has also been used to treat COVID-19 patients, as we will discuss further (204).

## COVID-19 Treatment

To date, no effective vaccines or therapeutic antiviral agents have been approved for the treatment of COVID-19 or any other human CoV infection. The main approach to disease management focuses on supportive care. To contain the viral transmission and disease, rapid public health interventions using immune cell-based therapies, antibodies, antivirals, new drugs, or vaccine strategies have focused on reducing mortality, virus spread, and mitigating potential future outbreaks. In this context, we conducted a survey of the main SARS-CoV-2 drugs/treatments following three criteria: peer-reviewed published scientific literature, with clinical trials that are underway, and that display a broad spectrum of action in the face of various viral and parasitic disease. The researched data (until September 2020) for ongoing and completed trials were searched in “clinicaltrials.gov”.

### Enhancing Immunity

As exposed in the previous topics, currently there are no proven treatments for SARS-CoV-2 infections, thus, much has been discussed about the maintenance of a healthy immune system. In this sense, the use of vitamins and other essential components for the proper functioning of the immune response can be an important approach in times of risk like this (205). Several studies have shown that the use of supplements helps in enhancing the immune response and recovery from viral infections, as is the case with the use of vitamin A and D or selenium to improve the humoral immunity of individuals vaccinated against influenza virus (206, 207), or the use of zinc to improve the immune response of individuals infected with torquetenovirus (208).

Among vitamin supplements, vitamin D stands out for having an immunomodulatory effect on both adaptive and innate immune responses, helping in the development of B, T, and NK cells. In addition, it has the ability to stimulate the production of antioxidant responses and microbicidal molecules such as defensins and cathelicidins (209). The use of vitamin D has also been associated with the prevention of respiratory diseases associated with viral infections (210), and epidemiological data suggest that vitamin D deficiency increases the susceptibility to acute viral respiratory infections (211). However, a study in the United Kingdom that evaluated plasmatic concentrations of vitamin D in samples from COVID-19 patients found no association between circulating vitamin D levels and the risk for disease severity (212).

The use of supplementation with other types of vitamins has also been described in viral infections; the use of vitamin C, for example, a potent antioxidant and an important enzyme cofactor, contributes to the development of the immune response, helping in the production of type I IFN. However, a systematic review with meta-analysis found no evidence that the use of vitamin C has any effect in preventing common cold infections (213). As for vitamin E, it has been shown that its deficiency can impair cellular and humoral immune responses (214). However, the use of vitamin E has been associated with an increased risk of pneumonia and has shown no significant effect in preventing lower respiratory tract infections (215, 216).

In view of the controversial results, more than 50 ongoing clinical trials are seeking to clarify the role of vitamins, minerals, and other dietary supplementation in the prophylaxis and treatment of COVID-19, analyzing parameters such as the risk of infection, risk of hospitalization, and clinical outcome.

### Immunotherapy

#### Antibody-Based therapy

Considered an efficient method for the clinical treatment of different infectious diseases, including MERS-CoV and SARS-CoV-1 (217), antibody-based immunotherapy has been studied as a potentially applicable tool to treat COVID-19. The mechanisms involved with its effects against SARS-CoV-2 are related to preventing the virus from entering the host cells, blocking its replication.

The virus entry block was studied for acting both in the cell receptor ACE2 and directly on the virus (neutralizing antibodies [nAbs]), specifically in the S1 subunit of the S protein (218–220). Regarding the blocking of ACE2 receptors, the application of some mechanisms stand out: the soluble version of ACE2 fused to an immunoglobulin Fc domain (ACE2-Fc), RDB domain attached to Fc (RDB-Fc), and receptor-targeted monoclonal antibodies (mAb) (221).

Viral neutralization by nAbs is also an immunotherapeutic approach and directly recognizes epitopic regions of SARS-CoV-2. This effect can be achieved either directly through mAbs manufactured in laboratories or by using polyclonal antibodies (pAbs) (218). nAbs act directly on the virus, preventing its infectivity by activating several pathways, such as the complement system, cell cytotoxicity, and phagocytic clearance (222–224).

The therapeutic use of mAbs has shown good outcomes, mainly due to its high specificity. Recently, several mAbs against viruses have been developed, including SARS-CoV-1 and MERS-CoV, in which the S protein is the major target described both *in vitro* and *in vivo*. According to some studies, the specific nAbs against SARS-CoV-1 RBD in the S protein could effectively block SARS-CoV-2 entry (218, 225). However, Wrapp et al. (226) tested several published SARS-CoV-1 RBD-specific nAbs and found that they do not have substantial binding to the S protein of SARS-CoV-2, suggesting that the cross-reactivity may be limited. Thus, the combination of nAbs with different viral targets and sources could improve treatment efficacy. In addition to experimental studies, to date, more than 10 clinical trials have aimed at testing human mAbs against SARS-Cov-2 (227–235), which could also represent an alternative, effective treatment.

Furthermore, some immunomodulatory mAbs have been tested in the context of COVID-19. It is remarkable that until now most of the data published regarding the use of immunomodulatory mAbs derive from studies using either anti-IL-6 or anti-IL-6R, probably because using IL-6 blockers seems promising at controlling the cytokine storm associated with the development of ARDS in more aggressive patterns of SARS-CoV-2 infection. However, clinical observations remain controversial.

Although some studies found considerable clinical improvements resulting from treatment with IL-6 blockers (236–239), others do not report any significant difference between the clinical features of groups treated with anti-IL6/IL-6R mAbs and their respective controls (without anti-IL-6/IL-6R) (240–243). These controversial results can be explained by the pleiotropic function of IL-6, which also play an important anti-inflammatory role, questioning the use of IL-6 blockade to suppress inflammation-induced tissue damage (244). Additionally, severe side effects have been associated with the use of IL-6 blockers, including enhanced hepatic enzymes, thrombocytopenia, severe bacterial and fungal infections, and sepsis (241, 245). In general, data from analyses on the use of this type of mAbs remain inconclusive (243, 246, 247).

Recent findings are optimistic, but data validation by robust scientific evidence has been hampered by the small sample size in most case reports and studies on the use of mAbs blocking other immune mediators, such as IL-1β, GM-CSF, and complement protein C5 (238, 248–250). However, seeking to verify the effectiveness of using mAbs blocking inflammatory mediators, dozens of clinical trials are currently underway.

Aiming at reducing the hyper inflammation found in the lungs of SARS-CoV-2-infected patients, different clinical studies are currently investigating the activities of mAbs anti-JAK, anti-GM-CSF, anti-GM-CSF receptor, anti-M-CSF receptor, anti-CD14, anti-IFNγ, anti-VEGF, anti-BKT, anti-CCR5, anti-IL-6, anti-IL-6 receptor, anti-TNFα, anti-IL1β, anti-IL1β receptor, and complement C5 inhibitor (220, 251, 252). Similarly, ongoing clinical trials have sought to reverse the hyper-thrombotic state found in critically ill patients by using anti-P-selectin, anti-CTGF, and factor XIIa antagonist mAbs (253, 254). Furthermore, to restore the exhausted T lymphocytes’ and NK cells’ immunity, other clinical studies applied anti-PD1 mAbs under the hypothesis of a stimulus of anti-viral response and prevention of ARDS (255–257).

More recently, the passive administration of pAbs has also been tested in COVID-19 patients (222–224, 258–267), also known as convalescent plasma (CP) or immune plasma, which is already used effectively and safely in the treatment of other severe acute respiratory syndrome infections of viral etiology, such as SARS, MERS, and H1N1, and offers only a short-term but rapid immunity to the susceptible individuals (268).

A strict criterion to select the CP donor states that the individual must show clinical recovery and test negative for the virus presence. Thus, after being confirmed, a high titer of neutralizing antibodies against SARS-CoV-2 must be stored in blood banks (269, 270).

Some reviews related to patients who received transfusion with CP showed a reduction in viral load, improvement in clinical symptoms, better radiological findings, and improved survival (260, 261, 271–273). In addition, after having received CP containing nAbs, COVID-19 patients had significant improvements from the beginning of treatment (until 22 days), presenting lower fever, decreased viral load, and higher nAbs levels. Further, 60% of the patients were discharged one month after the treatment (271). Better outcomes were found in early administration of CP (before SARS-CoV-2 seroconversion), preferably on day 5, for obtaining maximum efficacy (268).

More recently, Li et al. found no statistically significant clinical improvement or mortality reduction in a randomized clinical trial with CP-treated COVID-19 patients (274). However, the authors reported that CP treatment is potentially beneficial to critically ill patients by suggesting a possible antiviral efficiency of high titer of nAbs. Notably, there are clinical controversies, ethical issues, and potential risks associated with convalescent plasma therapy (275), such as the possibility of ADE development, exacerbating the disease severity, and causing a significant illness in future exposure to coronaviruses infection (268, 276, 277) (REF). Divergences between studies may be caused by variations in the composition of CP, which is highly variable and includes a variety of blood-derived components, timing of CP administration, titer of the specific antibody in administered plasma, and presence of blood borne pathogens (268). Nonetheless, understanding the efficacy and safety of CP therapy relies on the completion of the ongoing clinical trials.

Another therapeutic strategy using antibodies is intravenous immunoglobulin (IVIg) that contains polyclonal IgG isolated from healthy donors, which can be further enhanced by using IgG antibodies collected from recovered COVID-19 patients in the same geographical region as the patient. Results have been mostly positive, although many of these therapies have not been formally evaluated through a randomized, double-blind, placebo-controlled clinical trial (278). According to recent studies, IVIg can be used effectively in early stages of SARS-CoV-2 infection (before the initiation of systemic damage), reducing the use of mechanical ventilation, preventing the progression of pulmonary lesions, and promoting early recovery (268). Also, cross-neutralization activity was shown against SARS-CoV-2 in commercial IVIg manufactured prior to the COVID-19 pandemic and are currently under evaluation as potential therapies for COVID-19 (279). Thus, intravenous use of immunoglobulins can prove helpful in therapy against SARS-CoV-2, however, adjustments in the therapeutic regimen are necessary for all IVIg possibilities, as well as a complete understanding of the possible adverse effects, such as the risk of ADE (278, 279), that are being studied in more than 10 ongoing clinical trials.

Some works have shown that therapies focusing on the interaction between SARS-CoV-1 and the ACE2 receptor may be extended for use in SARS-CoV-2 patients as an immunotherapy tool (218). However, other authors refute this idea based on the fact that recent studies showed limited cross-neutralization between SARS-CoV-1 antibodies and SARS-CoV-2 (280, 281). Furthermore, it was shown that SARS-CoV-2 S protein binds ACE2 with a higher affinity than SARS-CoV-1, suggesting that such interaction differs between the two viruses (266).

#### Immune Cell-Based Therapy

In addition to antibody-based therapies, scientists have been studying immune cell-based therapies as a tool to combat COVID-19, focusing especially on NK and T cells. The importance of NK cells as the first antiviral responders can be seen in patients with NK cell deficiency and immunocompromised individuals who have increased susceptibility to viral infections (282). In this sense, Market et al. (282) gathered the main reports so far addressing potential therapies focusing on mediating NK cell activity to mitigate the immunopathological consequences of COVID-19, and consequently lighten the load on our health systems.

Some ongoing clinical trials have been studying the use of NK cell therapy through different approaches. A randomized phase I/II trial studied the infusions of CYNK-001 cells, an allogeneic off-the-shelf cell therapy enriched for CD56^+^/CD3^-^ NK cells expanded from human placental CD34^+^ cells in 86 hospitalized patients with moderate COVID-19 disease (283). Another randomized phase I/II study explored the use of NKG2D-ACE2 CAR-NK cells with each common, severe, and critical type COVID-19. The authors hypothesize that these cells target the S protein of SARS-CoV-2 and NKG2DL on the surface of infected cells with ACE2 and NKG2D, respectively, seeking out the elimination of SARS-CoV-2 virus particles and their infected cells (284).

The unregulated profile of the immune response in critically ill COVID-19 patients may be due to the reduction of Treg cells, which culminates in excessive release of inflammatory mediators and cytokine storms (153, 191–193). Thus, the use of adoptive transfer of these cells as a measure of inflammatory control in critically ill patients is a promising therapeutic approach. The infusion of autologous polyclonal Treg has already been used to treat inflammatory diseases, such as type 1 diabetes (285), however the use of autologous cells takes a long time, due to the period necessary for differentiation and clonal expansion, making this an unviable and costly method for infectious diseases, as is the case with COVID-19 (286).

A viable alternative is the use of allogeneic human leukocyte antigen-matched umbilical cord-derived Tregs (UBC-Treg) which can be widely expanded and used on a larger scale. A recent case study used 1x10^8^ administration of UBC-Treg in two patients with COVID-19 who had severe respiratory failure, and both demonstrated significant clinical improvement and reduced inflammatory markers four days after starting treatment (287).

There are currently two clinical trials underway that aim to infuse Treg cells in patients with severe COVID-19 and ARDS. The first one is a multi-center, prospective, double-blinded, placebo-controlled phase 1 randomized clinical trial, which has 45 patients who will receive cryopreserved UBC-Treg (288). The second one is a randomized, double-blind, placebo-controlled phase 2 study with 88 participants who will receive off-the-shelf allogeneic hybrid Treg/Th2 cells (RAPA-501-ALLO). RAPA-501-ALLO cells will be generated from healthy donors, cryopreserved, banked, and made available for off-the-shelf therapy. The cells are manipulated *ex vivo* to differentiate into two anti-inflammatory phenotypes simultaneously, generating hybrid Treg/Th2 cells, with the potential to reduce inflammation and mediate a protective effect on tissues (289).

In addition to therapeutic approaches using Treg cell infusion, another three clinical trials are underway with the aim of evaluating treatment using specific SARS-CoV-2 T cells isolated from individuals who recovered from COVID-19 (290–292). The use of virus-specific T cells for off-the-shelf treatment has been used in several viral infections, such as cytomegalovirus, HHV6, adenoviruses, Ebola virus, and BK virus (293–296). Although vaccination provides T cells-based virus-specific immunity, the path to its development is long, so the use of adoptive cell transfer techniques from healthy individuals who recovered from COVID-19 and developed an effective cell response is probably the fastest way to treat critically ill individuals (297). Besides that, as mentioned before, asymptomatic or mild symptomatic patients may possibly mount robust SARS-CoV-2 specific CD8+ T cell responses (200, 201), therefore, the use of these individuals’ cells to treat critically ill patients with COVID-19 can be a promising tool.

The clinical use of IL-7 has been implemented in the treatment of cancer patients and infectious diseases, mainly with the objective of improving the immune response by stimulating the generation of lymphocytes (298, 299). In addition, IL-7 administration has been reported to increase CD4 + and CD8 + T lymphocyte counts without inducing the production of pro-inflammatory mediators, making it a promising method of recovering immune function in patients with disorders related to cytokine storms, such as sepsis and COVID-19 (300).

In a case study conducted by Monneret et al. (301), compassionate administration of IL-7 to a patient with severe COVID-19 significantly improved total lymphocyte count and HLA-DR expression in circulating monocytes four days after administration of the first dose. The patient also showed a significant improvement in lung involvement and negative viral load. Another study conducted by Laterre et al. (302), who administered IL-7 to COVID-19 patients found that there was a significant improvement in the lymphocyte count after starting treatment, in addition, the patients did not show any change in TNF-α levels, IL-1β, and IL-12p70, which may indicate that IL-7 therapy may be safe for patients with severe inflammatory changes. Thus, the use of IL-7-based immunotherapy can be an important tool to be used in future clinical trials in patients with severe lymphopenia.

Therefore, the data available to date do not ensure the success of immunotherapy applied in patients with COVID-19, thus, further studies specifically targeting SARS-CoV-2 should be performed to provide more specific data. However, immunotherapy is effective and of immediate use, being of short duration. This approach also presented limitations, such as the possibility of abnormal reactions and other serious risks, such as induction of severe acute lung injury or ADE (225). Although we are living through a unique moment in science, with some mismatched information and novel, important discoveries being made every day, immunotherapy seems to be a possibly effective option to help patients until an effective, safe vaccine or treatment is developed.

### Drug Options Against SARS-CoV-2

Although some drugs appear to be effective against SARS-CoV-2 and are able to improve COVID-19 symptoms, there is no specific antiviral compound for this virus. In the face of such a global health emergency, several clinically used drugs are being reviewed and redirected to be tested in patients who have critical complications of COVID-19 in an attempt to eliminate the virus and modulate the patient’s immune response.

#### Antivirals

Due to the large amount of experimental and clinical studies assessing the effectiveness of antiviral therapy against SARS-CoV-2, we have seen the importance of this class of drugs in reducing the viral load peak at the beginning of the infection. Evidence from laboratory, animal, and clinical studies demonstrate that the use of associated or isolated antivirals can delay the progression of lung lesions and decrease the possibility of respiratory transmission of SARS-CoV-2. In this study, we selected the following most promising treatment options: lopinavir/ritonavir, arbidol, ribavirin, remdesivir, favipiravir, and type I IFN.

In the context of discovering new drugs, it is efficient to test the efficacy of existing antiviral drugs regarding the treatment of related viral infections. After the emergence of SARS in 2003, the screening of approved drugs identified an effective SARS-CoV-2 antiviral-drug candidate: the combination of the human immunodeficiency virus (HIV) protease inhibitors lopinavir and ritonavir. However, lopinavir has insufficient oral bioavailability for significant therapeutic activity due to rapid catabolism by the cytochrome P450 enzyme system. Thus, ritonavir is a cytochrome P450 and glycoproteins inhibitor, which increases the lopinavir plasma half-life, enhancing the pharmacokinetic and pharmacodynamic activities against the viral HIV-protease (303).

Chu et al. (304) described the possible mechanism of action of these drugs on SARS-CoV-1, suggesting that they act by inhibiting intracellular viral multiplication, preventing the action of the protease enzyme, which leads to the formation of an immature and less infectious virus with no ability to replicate.

Choy et al. (305) were successful at demonstrating the antiviral effect of lopinavir against SARS-CoV-2, but this was not the case for ritonavir. In turn, Kang et al. (306) found a lower viral load in infected SARS-CoV-2 Vero cells treated with lopinavir/ritonavir in relation to the untreated infected control. Although no consensus has been reached on its efficacy, dosage, or administration period, the literature includes some case reports, case series, and observational studies reporting a protective effect of the lopinavir/ritonavir combination in COVID-19 patients (110, 307–312).

Conversely, Cao et al. (313) conducted a controlled open‐label study with 199 hospitalized severe COVID‐19 patients randomly divided into two groups: a standard care group and a lopinavir/ritonavir treatment group (400 mg/100 mg). No benefit was observed in the lopinavir/ritonavir treatment group, showing no significant results for faster clinical improvement, lower mortality, or decreased viral RNA detectability. Although there are 85 clinical trials in progress testing lopinavir/ritonavir associated with other drugs on SARS-CoV-2 and/or COVID-19, WHO stopped the study of lopinavir/ritonavir in the Solidarity Trial.

Deng et al. (312) have studied the association of lopinavir/ritonavir with arbidol treatment and demonstrated a significant improvement in COVID-19 patients compared with a group treated only with lopinavir/ritonavir. Arbidol (umifenovir) is a broad-spectrum antiviral and immunomodulatory compound used to treat influenza and many other viruses (314). Analyses of molecular dynamics and structure-guided drug-binding have suggested an efficiency of arbidol at blocking or hampering the trimerization of the SARS-CoV-2 spike glycoprotein, in addition to inhibiting virus-cell interactions, which supports the potential use of arbidol to treat COVID-19 (315).

Chen et al. (316) demonstrated that arbidol therapy was able to shorten the course of the disease and promote clinical improvement, resulting in low fever and improvements in dry cough without side effects faster than the control group. Zhu et al. (317) also demonstrated the effects of arbidol by retrospectively analyzing the clinical data from 50 COVID-19 patients. The study demonstrated that the use of arbidol monotherapy, without association with other drugs, was more effective than the treatment with lopinavir/ritonavir, showing clinical improvement of the disease, presenting a total elimination of viral load over a shorter duration; in addition, no fever or ARDS were reported compared with those in the lopinavir/ritonavir group.

Ribavirin is another antiviral drug used in association with lopinavir/ritonavir to treat SARS-CoV-1 and was able to reduce viral load, risk of adverse clinical outcomes, ARDS, or death in SARS patients (304, 318). Ribavirin has a broad antiviral spectrum as it is a nucleotide analog that competes for the active site of RdRp, a crucial enzyme in the life cycle of RNA viruses, inhibiting viral replication and transcription (221, 319, 320).

Elfiky (320) conducted an *in silico* study demonstrating that ribavirin and other antivirals such as sofosbuvir can strongly bind to coronavirus RdRp, preventing the transcription of new copies of viral RNA. Only a few clinical studies have investigated the effect of ribavirin on COVID-19 patients, with the studies available generally focusing on the association of ribavirin and other therapeutic schemes (321–323). Nevertheless, China’s government (324) has recommended the use of ribavirin in COVID-19 patients.

Remdesivir (RDV) is also among the several potential drugs tested for SARS-CoV-2 treatment. Originally developed to treat Ebola virus infection, RDV is active against RNA viruses from different families, including Coronaviridae (e.g., SARS-CoV-1 and MERS-CoV) (325). RDV showed an *in vitro* effective antiviral activity against SARS-CoV-2 (326). Grein et al. (327) conducted a cohort study with 53 COVID-19 patients treated with RDV and found that 68% of them had improved oxygen-support class, whereas 57% of the patients receiving mechanical ventilation were extubated. Overall mortality reached 13% over a median follow-up of 18 days, however, viral load data were not collected to confirm the antiviral effects of RDV. The biggest issue with this study is that the authors did not include a group without RDV, which hampers the performance of comparative statistical analyses to prove whether the data found resulted from the treatment with RDV.

Another double-blind, randomized, placebo-controlled trial of intravenous RDV conducted in adults hospitalized with COVID-19 with evidence of lower respiratory tract involvement was performed in different parts of the world (328). The study of Beigel and colleagues (328) enrolled 1,063 COVID19 pneumonia patients, 538 of whom were assigned to the treatment with RDV and 521 to a placebo, showed the effectiveness of RDV in treating COVID-19 patients. The drug was superior to the placebo in reducing the recovery time in hospitalized COVID-19 patients and decreased the mortality rate in the RDV group, however, this result did not reach statistical significance.

Antionori et al. (329), analyzing patients with severe COVID-19 pneumonia in an intensive care unit (ICU) who were treated for 10 days with RDV, found that on the 28^th^ day, 38.9% showed improvement, 16.7% were still on mechanical ventilation, and 44.4% died. The data suggest that this treatment can benefit hospitalized patients who are not in the ICU, where the clinical result was better and adverse events are observed less frequently.

Alternatively, the randomized, double-blind, placebo-controlled, multicenter trial with 273 ill individuals performed by Wang et al. revealed that RDV intravenous administration was well-tolerated in COVID-19 patients. However, the authors did not find any clinical improvement or significant antiviral effect. Goldman et al. (330), in another phase 3 clinical trial on 397 patients with severe COVID-19 without mechanical ventilation support, also did not find differences between 5-day and 10-day courses of RDV therapy. The RDV data currently available are still controversial, however, dozens of clinical studies are currently using this drug as an alternative treatment for COVID-19, possibly further elucidating its effects.

The efficiency of favipiravir, another anti-influenza RdRp inhibitor, has also been clinically assessed and was approved for COVID-19 treatment in China, March 2020 (331, 332). An experimental study carried out with the VERO cell line showed that the drug has *in vitro* activity against SARS-CoV-2 (326). Aiming at comparing the effects of favipiravir and lopinavir/ritonavir, Cai et al. (333) conducted an open, non-randomized, before-after controlled study with 80 patients and found that favipiravir favored viral clearance and improved chest CT, having caused fewer adverse effects than the lopinavir/ritonavir group. Currently, 31 clinical trials using this medication are in progress.

Regarding antivirals, type I IFN is a group of cytokines comprising the α and β subtypes, among others, with an important role in antiviral immunity that interferes with viral replication, as discussed above. Many studies have shown the protective effect of type I IFN associated with antiviral therapies for patients with SARS and MERS [reviewed by Sallard et al. (334)], which arouses the interest of the scientific community in type I IFN as a potential treatment against SARS-CoV-2 (334–337). Despite their efficacy against SARS-CoV-2 (338, 339), the results of *in vitro* studies using IFN-α and -β to treat COVID-19 patients remain inconclusive.

Such uncertain nature of the results is associated with biases present in these studies, which include limited-size sample, heterogeneous experimental designs/clinical status, and the type of IFN isoform tested. In addition, since COVID-19 treatments rarely involve monotherapy, it is difficult to assess whether the results derived from the tested IFN or the drugs used in combination (322, 323, 340–343). It is also worth mentioning that, as discussed above, type I IFN appears to exacerbate inflammation in the progression to severe COVID-19; the timing of administration and subgroups targeted for treatment with type I IFN need to be considered with caution.

A recent retrospective multicenter cohort study of 446 Chinese patients with COVID-19 reported that among severe to critical COVID-19 patients, early administration (≤5 days after admission) of IFN-α2b decreased mortality in comparison with no admission of IFN-α2b, whereas no significant benefit was associated with IFN-α2b use in moderately ill patients. However, late use of IFN-α2b increased mortality and delayed recovery of severe to critical COVID-19 patients (344).

Zhou et al. (341), investigated the isolated effect of IFN-α in a cohort study comparing 77 patients with moderate COVID-19 treated with nebulized IFN-α2b (5 mU b.i.d.), oral arbidol (200 mg t.i.d.), or a combination of both. Although the study did not include a control group, the treatment with IFN-α2b, either containing arbidol or not, significantly reduced the duration of detectable virus in the upper respiratory tract and the circulating of inflammatory markers (IL-6 and C-reactive protein levels).

Still, in a retrospective multicenter cohort study with 141 mild COVID-19 patients by Xu et al. (342), the arbidol/IFN-α2b combination proved more effective in accelerating pneumonia recovery than IFN-α2b monotherapy, but this was not the case for viral clearance or reducing the length of hospital stay than IFN-α2b monotherapy.

Hung et al. (322) assessed the effect of IFN-β on COVID-19 patients and found that the triple combination of IFN-β1b, lopinavir/ritonavir, and ribavirin was safer and more effective than lopinavir/ritonavir alone at alleviating symptoms, shortening the duration of viral shedding and hospital stay in patients with mild to moderate COVID-19. Similarly, an open randomized clinical trial was carried out by Danoudi-Monfared et al. (345), analyzing treatment with IFN-β-1a. The IFN group of COVID-19 patients (n=42) received IFN β-1a in addition to the protocol medications (hydroxychloroquine plus lopinavir-ritonavir or atazanavir-ritonavir) while the control group (n=39) received only the protocol medications. The IFN-β-1a-treated patients showed a significantly increased discharge rate on day 14 and decreased mortality within 28 days. A better survival rate was also observed when patients received IFN- β-1a in the early stage of the disease.

The COVID-19 treatment guidelines of many countries already recommend the use of IFNs α/β (335). Currently, all over the world, more than 20 clinical trials are using IFN-α and/or β alone or in association with other drugs.

#### Chloroquine and Hydroxychloroquine

Chloroquine and hydroxychloroquine have been used worldwide for more than 70 years, and they are part of the WHO model list of essential medicines (346). They were synthesized specifically for the treatment and chemoprevention of malaria, but their immunomodulatory activity led these drugs to be used against autoimmune diseases, such as rheumatoid arthritis, systemic lupus erythematosus, and other inflammatory rheumatic diseases; they also show broad-spectrum antiviral effects (347–349).

Regarding the chemical structure, hydroxychloroquine differs from chloroquine in the presence of a hydroxyl group at the end of the side chain: the N-ethyl substituent is β-hydroxylated. Both drugs have similar pharmacokinetics, with rapid gastrointestinal absorption and renal elimination, but different clinical indications and toxic doses, in which hydroxychloroquine is less toxic and more clinically used in the malaria model (348, 350).

The action mechanism of these drugs has direct molecular effects on lysosomal activity, autophagy, and signaling pathways (347). As antivirals, chloroquine is known to block SARS-CoV-1-infection by increasing endosomal pH required for virus entry, as well as interfering with the glycosylation of cellular receptors (351, 352). The possible mechanism against SARS-CoV-2 is the inhibition of virus entry by altering the glycosylation of ACE2, reducing the binding efficiency between ACE2 in host cells and the S protein on the surface of the SARS-CoV-2, thus preventing the virus from binding to target cells (348, 351, 353). In addition to a potent antiviral inhibition, the immunomodulatory activity of these drugs is well established in the literature. Proposed effects of chloroquine on the immune system include increasing the export of soluble antigens into the cytosol of dendritic cells, the blocking of TLR7 and TLR9 signaling, thus reconstructing CD8^+^ cytotoxic viral response, and inhibiting and/or reducing the production of inflammatory cytokines like IL-1, IL-6, TNF, and IFN-α (347, 354–359), which has an important role in the immunopathogenesis of COVID-19, as previously reported in item 4.1.

*In vitro* studies on SARS-CoV-2 have demonstrated the low-dose action of these drugs, having found the lowest half-maximal effective concentrations (EC50s). In addition, their association with azithromycin significantly inhibited viral replication (326, 360–362). In humans, a study by Gao, Tian, and Yang (363) showed that patients treated with chloroquine phosphate had inhibited exacerbation of pneumonia, improving lung imaging findings, promoting a virus-negative conversion, and shortening the COVID-19 course.

The association of hydroxychloroquine with other drugs is also suggested, with emphasis on studies using azithromycin, a broad-spectrum macrolide antibiotic primarily used to treat respiratory, enteric, and genitourinary bacterial infections. Despite not yet being approved for antiviral therapy, it has been studied *in vitro* and in clinical trials for activity against several viruses (364).

Gautret et al. (365) demonstrated the effectiveness of the hydroxychloroquine-azithromycin combination in a non-randomized clinical trial with 36 COVID-19 patients. A 57.1% rate of cure was attributed to the patients treated with hydroxychloroquine, however, when combined with azithromycin, 100% of the patients were cured. The authors suggested a synergistic effect of the drug combination since both were reported to have antiviral and immunomodulatory activity in the literature.

Gautret et al. (366) conducted another analysis to provide evidence of a beneficial effect of co-administration of hydroxychloroquine with azithromycin in a non-comparative and uncontrolled observational study with 80 mildly infected SARS-CoV-2 patients. The hydroxychloroquine/azithromycin treatment showed that 81.3% of the patients had a favorable result with a rapid decrease in nasopharyngeal viral load at day 8 (93%), reducing the mean length of stay in the hospital.

Arshad et al. (367) performed a multicenter observational study, which included 2541 COVID-19 patients. Patients were separated into four groups: untreated (n = 409), treated with hydroxychloroquine (n = 1202), the association of hydroxychloroquine and azithromycin, and azithromycin only (n = 147). The authors suggested that the treatment with hydroxychloroquine alone and in combination with azithromycin was associated with a reduction in the hazard ratio for death when compared with receipt of neither medication.

However, a lot of controversy has been raised about these data, and many important limitations of this study were considered by several authors (368–373), threatening the validity of the reported findings. Among these, there is the potential for immortal time bias and selection bias, the administration of corticosteroids in most patients treated with hydroxychloroquine than in other groups, and a disproportionately high share of patients with cardiovascular comorbidity in the untreated group.

Seeking to analyze the efficacy of early treatment using hydroxychloroquine and azithromycin, Million et al. (374) carried out a retrospective study with 1061 SARS-CoV-2 infected patients. In the study, 91.7% of the patients reached good clinical results and virological cure within 10 days, while 4.3% had a poor outcome associated with advanced age. However, it is worth mentioning that the study did not include a control group to establish a comparison.

To assess the use of hydroxychloroquine as a prophylactic measure, Boulware et al. (375) performed a randomized, double-blind trial in adults who had been exposed to individuals diagnosed with COVID-19, either in the home or work environment. The authors found that postexposure prophylaxis did not prevent the development of the disease.

An important question that may be considered about chloroquine and its derivate is the numerous adverse effects reported, such as nausea, pruritus, headache, hypoglycemia, neuropsychiatric effects, and idiosyncratic hypersensitivity reactions. In long-term treatments, effects such as retinopathy, vacuolar myopathy, neuropathy, restrictive cardiomyopathy, and cardiac conduction disorders are also reported. Furthermore, its concomitant use with azithromycin may predispose patients to arrhythmias (213), which represents a major negative implication.

Huang et al. (376) conducted a randomized clinical trial with 22 patients in China to compare the effects of chloroquine and lopinavir/ritonavir. Even though chloroquine led to some clinical improvement, half of the patients experienced adverse effects such as vomiting, abdominal pain, nausea, diarrhea, skin rashes, cough, and shortness of breath.

Satlin et al. (377), Magagnoli et al. (378), Rosenberg et al. (379), and Ip et al. (380) reported that treatment with hydroxychloroquine, azithromycin, or both were not associated with a survival benefit among patients and there were no significant differences in mortality for patients receiving hydroxychloroquine during hospitalization. Similarly, Mahévas et al. (381) analyzed the efficacy of hydroxychloroquine in patients hospitalized with coronavirus pneumonia who needed oxygen but not intensive care, through a comparative observational study. 181 patients were analyzed, 84 of whom received hydroxychloroquine. Data showed there was no effect on reducing admissions to intensive care or deaths on day 21 after hospital admission and the hydroxychloroquine treatment did not have any effect on survival without acute respiratory distress syndrome on day 21 after hospital admission.

Tang et al. (382) carried out a multicenter, open, randomized, and controlled clinical trial evaluating 150 patients admitted with confirmed mild to severe COVID-19; of these, 75 were treated with hydroxychloroquine. The authors demonstrated that treatment does not contribute to the elimination of the virus.

Borba et al. (383) conducted a phase IIb, double-blind, randomized clinical trial comparing the effects of high doses (600 mg/twice daily for 10 days) and low doses (450 mg twice daily at day 1 and once daily for 4 days) of chloroquine in 81 and 40 patients, respectively. The results did not evidence lower viral load in respiratory secretions, not even in combination with azithromycin. The mortality rate for the high-dose group was over twice as high as the low-dose group (39.0% vs. 16.0%). Additionally, some patients, mainly in the high-dose group, showed adverse effects, such as increased creatine phosphokinase (CK) and CK-MB, while the high-dosage group exhibited more corrected QT (QTc) interval prolongation. Neither of the dosages was able to influence lethality. The authors concluded that critically ill patients should not receive chloroquine at high doses.

In the meantime, a cohort study with 201 patients showed that the use of chloroquine or hydroxychloroquine combined with azithromycin generated a higher increase in QT prolongation than chloroquine or hydroxychloroquine monotherapy (384). More recently, another large observational study involving 1376 cases of COVID-19 from New York found no significant association between the use of hydroxychloroquine and intubation or death (385).

Currently, chloroquine and hydroxychloroquine are the most largely studied compounds in the context of COVID-19 treatment, encompassing at least 320 ongoing clinical trials. However, considering that more recent studies failed to prove any favorable effect of their use in COVID-19 patients, the WHO discontinued the study of hydroxychloroquine in the Solidarity Trial (13).

#### Antihelminthics

Amid the COVID-19 pandemic, the search for active molecules against the coronavirus should use advanced tools of computational biology and artificial intelligence for the recognition of drugs already approved and commercialized with potential effects on the replication of SARS-CoV-2 (386).

In this context, over the past few years, research has shown the antiviral potential *in vitro*, especially against RNA viruses, of Ivermectin, the best known and most widely used antiparasitic drug in human and veterinary medicine, with promising results against SARS-CoV-2 (387). The model of Vero/hSLAM cells infected with a SARS-CoV-2 isolate showed the ivermectin antiviral effect in which 24h-ivermectin treatment reduced 93% of RNA viral load in the cell supernatant and 99.8% of the intracellular viral RNA. The authors hypothesized that its probable mechanism of action occurs through the inhibition of nuclear import of importin-α/β1–mediated the IMPα/β1 heterodimer of viral proteins, as shown for other RNA viruses (387, 388). Corroborating, Lehrer and Rheinstein (389) identified the ivermectin docking site between the region of leucine 91 of viral spike and the histidine 378 of the ACE2 receptor, which may interfere with the attachment of the spike to the human cell membrane.

Although the *in vitro* proliferation inhibition effect of Ivermectin against SARS-CoV-2 has been shown, there is no evidence that the IC50 of ~ 2 µM determined by Caly and colleagues can be achieved in the clinic where pharmacokinetics studies showed that even the maximum tested dosage of 1700 μg/kg presented only 0.28μM of plasma concentration (390).

According to Navarro et al. (391), no adverse effects of high doses of ivermectin have so far been demonstrated in clinical studies with patients, with only a few transient ocular events in those who experienced high doses (up to 400 μg/kg). However, Duthaler et al. (392) demonstrated that the adverse effects of ivermectin in the body can vary according to the patient’s nutritional status, and the effects of high doses can be harmful, especially in patients with malnutrition levels. The general consensus of the authors is that further studies are needed to evaluate the efficacy and safety of ivermectin administered in high doses against SARS-CoV-2.

Xu et al. (386) published a review article regarding niclosamide, an old anthelmintic used to treat tapeworm infections, showing promising antiviral activity against various viral infections, such as SARS-CoV-1 and MERS-CoV. This drug has shown to act *in vitro* by enhancing autophagy and efficiently reducing MERS-CoV replication.

Originally developed as an antiprotozoal agent, nitazoxanide is another broad-spectrum antiviral agent that has been currently developed to treat influenza and other viral respiratory infections. Nitazoxanide exhibited *in vitro* activity against MERS-CoV by inhibiting the expression of viral N protein, in addition to reducing the production of IL-6 in an *in vivo* model (393, 394).

Despite the lack of studies in the literature showing the effect of these anthelmintics on the COVID-19 model, clinical trials have currently included this type of antiviral agent in many countries; there are 37 clinical trials using ivermectin alone or associated with hydroxychloroquine, and 19 with nitazoxanide. These studies are yet to be published and preliminary results are expected in the second half of 2020.

#### Anticoagulants

A high mortality risk in severe COVID-19 patients has been described, especially due to the development of disseminated intravascular coagulation and coagulopathy (395). Patients with sepsis and disseminated intravascular coagulation may develop thromboembolic complications or microvascular clot deposition, contributing to multiple organ failure. In patients with severe pneumonia, the activation of vascular endothelium, platelets, and leukocytes results in the unregulated generation of thrombin, both locally, in the lungs, and systemically, leading to fibrin deposition and subsequent tissue damage and microangiopathy (396). In COVID-19 patients, severe pulmonary inflammation is believed to be associated with the regulation of pro-inflammatory cytokines, which can cause the dysfunction of endothelial cells and consequently higher thrombin production. Therefore, the use of anticoagulant therapy could be beneficial for COVID-19 patients (397).

In a retrospective study with 449 patients with severe COVID-19, Tang et al. (395) observed a lower mortality rate in individuals treated with prophylactic heparin associated with coagulopathy compared with those who had not been treated with an anticoagulant. The study associated the use of thrombosis prophylaxis with lower 28-day mortality in COVID-19 patients, but only for those presenting a high value of either sepsis-induced coagulopathy score (≥4) or D-dimer (≥3.0 mg/L).

Paranjpe et al. (398) carried out a large cohort analysis with 2773 COVID-19 patients in the United States, among which 28% received anticoagulant therapy, and also found an association of anticoagulant-based treatment with lower mortality risk. The mortality rate in patients who required mechanical ventilation and received anticoagulant therapy was lower than those who had not been treated with an anticoagulant.

It is important to highlight that heparin has an anti-inflammatory effect that can bind to inflammatory cytokines, chemokines, and proinflammatory proteins, inhibiting neutrophil chemotaxis and leukocyte migration (399–401). In the current COVID-19 context, there are over 60 ongoing clinical trials covering the use of thromboprophylaxis, which will certainly clarify the potential role of anticoagulants in patients with COVID-19.

#### Dexamethasone

Recent studies have demonstrated great interest in the role of corticosteroids to attenuate the pulmonary and systemic damage in COVID-19 patients because of their potent anti-inflammatory and antifibrotic properties, especially dexamethasone, a synthetic corticosteroid which is on the list of essential medicines of the World Health Organization and is readily available worldwide at low cost. This drug acts as a broad-spectrum immunosuppressor and has greater activity in inflammatory and autoimmune conditions (402, 403).

Recently, the randomized RECOVERY study, conducted by the University of Oxford, declared dexamethasone as the world’s first treatment proven effective in reducing the risk of death among severely ill COVID-19 patients. The trial accompanied a total of 2104 patients treated with dexamethasone and 4321 who received conventional care. The dexamethasone group showed reduced 28-day mortality in COVID-19 patients receiving invasive mechanical ventilation or oxygen therapy without invasive mechanical ventilation, but not in patients who were not receiving any respiratory support (404).

Similar results were published by Tomazini et al. (405) in a Brazilian multicenter, randomized, open-label, clinical trial involving 299 adults with moderate or severe ARDS due to COVID-19. The study showed that 144 patients who received dexamethasone treatment plus the standard treatment showed a significant increase in the number of days without mechanical ventilation during the first 28 days. In the same way, Villar et al. (406) also published a multicenter randomized clinical trial and showed that early administration of dexamethasone in COVID-19 patients who had moderate and severe ARDS presented an increased average number of days without mechanical ventilation, as well as reduced mortality compared to the control group. There are currently 29 clinical trials evaluating the therapeutic efficacy of dexamethasone in COVID-19 patients.

In the face of the huge amount of studies involving clinical trials to test drugs for SARS-CoV-2 and COVID-19 treatment, in addition to the different research methodologies and criteria addressed, on March 22, 2020 the WHO and partners launched the “SOLIDARITY”, an international clinical trial. The purpose is to help find an effective treatment for COVID-19, seeking to establish consistent endpoints, control arms, and inclusion-exclusion criteria for this umbrella trial (13).

The SOLIDARITY trial includes hospitalized patients with COVID-19 from more than 90 countries around the world to compare treatment options with standard care and assess their relative effectiveness against SARS-CoV-2. By enrolling patients from multiple countries, the SOLIDARITY trial aims at rapidly discovering if any of the drugs mitigate disease progression or improve survival. According to the WHO director-general, the study will dramatically cut the time needed to generate robust evidence on how the drugs work. Thus, the two most promising treatment options selected were Remdesivir or Lopinavir/Ritonavir with IFN-β. Other drugs can be added based on emerging evidence (13).

## Conclusion

In conclusion, this collection of works suggests that the genomic changes of SARS-CoV-2 are responsible for its higher transmissibility rate and severity in relation to other hCoVs. Furthermore, the process of tropism and invasion of the virus is favored by its capacity of high-affinity bonding to the human ACE2 receptor. Cytokines have a direct role in the immunopathogenesis of COVID-19 by inducing the hyper inflammation and lung injury peculiar to the disease. Benefits of IFN-mediated response seem to occur only during early infection, and the failed control of its production over time might be related to the worsening of the disease. Monocytes and macrophages have an important role in respiratory failure during COVID-19; several studies have reported that these cells migrate to the lungs, producing pro-inflammatory cytokines, like IL-6, and inducing epithelial damage. Controversially, at later stages, COVID-19 patients present an impaired immune response due to exhausted phenotype and lower effector T cells, CD8^+^ T lymphocytes, and NK cells, culminating in antiviral immunity loss. Theoretically, the production of specific antibodies against SARS-CoV-2 by the immune system should be able to combat the virus and reduce viral load, but in critically ill patients, it does not seem to occur effectively, contributing to the severity of the disease. Unfortunately, no effective vaccines or therapeutic antiviral agents have been approved for the treatment of COVID-19 so far, but immunotherapy and some repositioned drugs originally used to treat inflammatory and coagulation disorders and viral and parasitic infections are ongoing clinical trials. This is a unique moment in science and humanity, with some mismatched information, as well as novel, important discoveries being made every day, therefore, all information must be interpreted carefully. Our review encompassed the most relevant articles in the area seeking to disseminate good-quality information.

## Author Contributions

TS, FT-P, RS: study design, data collection, and manuscript writing. MG, BB, MD, AR, AC, VC, and ES: data collection and manuscript writing. IC, WP, and IC-C: text correction and organization. MM-S: study design, text correction, and organization. All authors contributed to the article and approved the submitted version. The corresponding authors attest that all listed authors meet authorship criteria and that no others meeting the criteria have been omitted.

## Conflict of Interest

The authors declare that the research was conducted in the absence of any commercial or financial relationships that could be construed as a potential conflict of interest.

## References

[1] World Health Organization WHO Coronavirus Disease (COVID-19) Dashboard | WHO Coronavirus Disease (COVID-19) Dashboard (2020). Available at: https://covid19.who.int/ (Accessed September 14, 2020).

[2] Park M Cook AR Lim JT Sun Y Dickens BL A Systematic Review of COVID-19 Epidemiology Based on Current Evidence. J Clin Med (2020) 9:967. 10.3390/jcm9040967 PMC7231098 32244365

[3] Wu Y Ho W Huang Y Jin DY Li S Liu SL SARS-CoV-2 is an appropriate name for the new coronavirus. Lancet (2020) 395:949–50. 10.1016/S0140-6736(20)30557-2 PMC7133598 32151324

[4] Andersen KG Rambaut A Lipkin WI Holmes EC Garry RF The proximal origin of SARS-CoV-2. Nat Med (2020) 26:450–2. 10.1038/s41591-020-0820-9 PMC7095063 32284615

[5] Wu Z McGoogan JM Characteristics of and Important Lessons from the Coronavirus Disease 2019 (COVID-19) Outbreak in China: Summary of a Report of 72314 Cases from the Chinese Center for Disease Control and Prevention. JAMA J Am Med Assoc (2020) 323:1239–42. 10.1001/jama.2020.2648 32091533

[6] Lin L Lu L Cao W Li T Hypothesis for potential pathogenesis of SARS-CoV-2 infection–a review of immune changes in patients with viral pneumonia. Emerg Microbes Infect (2020) 9:1–14. 10.1080/22221751.2020.1746199 32196410 PMC7170333

[7] Hoffmann M Kleine-Weber H Schroeder S Krüger N Herrler T Erichsen S SARS-CoV-2 Cell Entry Depends on ACE2 and TMPRSS2 and Is Blocked by a Clinically Proven Protease Inhibitor. Cell (2020) 181:271–80.e8. 10.1016/j.cell.2020.02.052 32142651 PMC7102627

[8] Letko M Marzi A Munster V Functional assessment of cell entry and receptor usage for SARS-CoV-2 and other lineage B betacoronaviruses. Nat Microbiol (2020) 5:562–9. 10.1038/s41564-020-0688-y PMC7095430 32094589

[9] Zheng H-Y Zhang M Yang C-X Zhang N Wang X-C Yang X-P Elevated exhaustion levels and reduced functional diversity of T cells in peripheral blood may predict severe progression in COVID-19 patients. Cell Mol Immunol (2020) 17:541–3. 10.1038/s41423-020-0401-3 PMC7091621 32203186

[10] Zheng M Gao Y Wang G Song G Liu S Sun D Functional exhaustion of antiviral lymphocytes in COVID-19 patients. Cell Mol Immunol (2020) 17:533–5. 10.1038/s41423-020-0402-2 PMC7091858 32203188

[11] World Health Organization Clinical management of severe acute respiratory infection when COVID-19 is suspected - Interim guidance V 1.2 (2020). Available at: https://www.who.int/publications-detail/clinical-management-of-severe-acute-respiratory-infection-when-novel-coronavirus-(ncov)-infection-is-suspected (Accessed April 22, 2020).

[12] World Health Organization Infection prevention and control during health care when novel coronavirus (nCoV) infection is suspected (2020). Available at: https://www.who.int/publications-detail/infection-prevention-and-control-during-health-care-when-novel-coronavirus-(ncov)-infection-is-suspected-20200125 (Accessed April 22, 2020).

[13] WHO WHO “Solidarity” clinical trial for COVID-19 treatments . Available at: https://www.who.int/emergencies/diseases/novel-coronavirus-2019/global-research-on-novel-coronavirus-2019-ncov/solidarity-clinical-trial-for-covid-19-treatments (Accessed July 9, 2020).

[14] Tu YF Chien CS Yarmishyn AA Lin YY Luo YH Lin YT A review of sars-cov-2 and the ongoing clinical trials. Int J Mol Sci (2020) 21:2657. 10.3390/ijms21072657 PMC7177898 32290293

[15] Shi Z-L Guo D Rottier PJM Coronavirus: epidemiology, genome replication and the interactions with their hosts. Virol Sin (2016) 31:1–2. 10.1007/s12250-016-3746-0 26908210 PMC7091033

[16] Li F Structure, Function, and Evolution of Coronavirus Spike Proteins. Annu Rev Virol (2016) 3:237–61. 10.1146/annurev-virology-110615-042301 PMC5457962 27578435

[17] Schoeman D Fielding BC Coronavirus envelope protein: current knowledge. Virol J (2019) 16:69. 10.1186/s12985-019-1182-0 31133031 PMC6537279

[18] Li R Pei S Chen B Song Y Zhang T Yang W Substantial undocumented infection facilitates the rapid dissemination of novel coronavirus (SARS-CoV2). Science (2020) 368(6490):489–93. 10.1126/science.abb3221 PMC7164387 32179701

[19] Woo PCY Lau SKP Lam CSF Lai KKY Huang Y Lee P Comparative analysis of complete genome sequences of three avian coronaviruses reveals a novel group 3c coronavirus. J Virol (2009) 83:908–17. 10.1128/JVI.01977-08 PMC2612373 18971277

[20] Woo PCY Lau SKP Lam CSF Lau CCY Tsang AKL Lau JHN Discovery of seven novel Mammalian and avian coronaviruses in the genus deltacoronavirus supports bat coronaviruses as the gene source of alphacoronavirus and betacoronavirus and avian coronaviruses as the gene source of gammacoronavirus and deltacoronavi. J Virol (2012) 86:3995–4008. 10.1128/JVI.06540-11 22278237 PMC3302495

[21] Lam TT-Y Shum MH-H Zhu H-C Tong Y-G Ni X-B Liao Y-S Identifying SARS-CoV-2 related coronaviruses in Malayan pangolins. Nature (2020) 583:282–5. 10.1038/s41586-020-2169-0 32218527

[22] Wan Y Shang J Graham R Baric RS Li F Receptor Recognition by the Novel Coronavirus from Wuhan: an Analysis Based on Decade-Long Structural Studies of SARS Coronavirus. J Virol (2020) 94:e00127-20. 10.1128/jvi.00127-20 31996437 PMC7081895

[23] Holmes EC The Evolution and Emergence of RNA Viruses (2009). Available at: https://books.google.com.br/books?hl=pt-BR&lr=&id=fpoUDAAAQBAJ&oi=fnd&pg=PR9&dq=The+evolution+and+emergence+of+RNA+viruses&ots=6krXgdrz2Y&sig=sbZ8bUimug4XX1WLSq5_WiB7bsI#v=onepage&q=TheevolutionandemergenceofRNAviruses&f=false (Accessed April 1, 2020).

[24] Hu B Ge X Wang LF Shi Z Bat origin of human coronaviruses Coronaviruses. Virol J (2015) 12:221. 10.1186/s12985-015-0422-1 26689940 PMC4687304

[25] Zhu N Zhang D Wang W Li X Yang B Song J A novel coronavirus from patients with pneumonia in China, 2019. N Engl J Med (2020) 382:727–33. 10.1056/NEJMoa2001017 PMC7092803 31978945

[26] Huang C Wang Y Li X Ren L Zhao J Hu Y Clinical features of patients infected with 2019 novel coronavirus in Wuhan, China. Lancet (2020) 395:497–506. 10.1016/S0140-6736(20)30183-5 31986264 PMC7159299

[27] Assiri A Al-Tawfiq JA Al-Rabeeah AA Al-Rabiah FA Al-Hajjar S Al-Barrak A Epidemiological, demographic, and clinical characteristics of 47 cases of Middle East respiratory syndrome coronavirus disease from Saudi Arabia: A descriptive study. Lancet Infect Dis (2013) 13:752–61. 10.1016/S1473-3099(13)70204-4 PMC7185445 23891402

[28] Cascella M Rajnik M Cuomo A Dulebohn SC Di Napoli R Features, Evaluation and Treatment Coronavirus (COVID-19). In: StatPearls [Internet]. (Treasure Island (FL): StatPearls Publishing) (2020). 32150360

[29] World Health Organization Coronavirus disease (COVID-19) Situation Report-170.

[30] Gt Walker P Whittaker C Watson O Baguelin M Ainslie KEC Bhatia S Report 12: The Global Impact of COVID-19 and Strategies for Mitigation and Suppression. Imperial College (2020). 10.25561/77735

[31] Banerjee A Kulcsar K Misra V Frieman M Mossman K Bats and coronaviruses. Viruses (2019) 11:41. 10.3390/v11010041 PMC6356540 30634396

[32] Giwa A Desai A Novel coronavirus COVID-19: an overview for emergency clinicians. Emerg Med Pract (2020) 22:1–21. 32105049

[33] Zhang T Wu Q Zhang Z Probable Pangolin Origin of SARS-CoV-2 Associated with the COVID-19 Outbreak. Curr Biol (2020) 30:1346–51.e2. 10.1016/j.cub.2020.03.022 32197085 PMC7156161

[34] Li X Zai J Zhao Q Nie Q Li Y Foley BT Evolutionary history, potential intermediate animal host, and cross-species analyses of SARS-CoV-2. J Med Virol (2020) 92:jmv.25731. 10.1002/jmv.25731 PMC7228310 32104911

[35] Tiwari R Dhama K Sharun K Iqbal Yatoo M Singh Malik Y Singh R Veterinary Quarterly COVID-19: animals, veterinary and zoonotic links. Vet Q (2020) 40(1):169–82. 10.1080/01652176.2020.1766725 PMC7755411 32393111

[36] Cagliani R Forni D Clerici M Sironi M Computational inference of selection underlying the evolution of the novel coronavirus, SARS-CoV-2. J Virol (2020) 94:e00411-20. 10.1128/jvi.00411-20 32238584 PMC7307108

[37] Lu R Zhao X Li J Niu P Yang B Wu H Genomic characterisation and epidemiology of 2019 novel coronavirus: implications for virus origins and receptor binding. Lancet (2020) 395:565–74. 10.1016/S0140-6736(20)30251-8 PMC7159086 32007145

[38] Zhou P Yang XL XG W Hu B Zhang L Zhang W A pneumonia outbreak associated with a new coronavirus of probable bat origin. Nature (2020) 579:270–3. 10.1038/s41586-020-2012-7 PMC7095418 32015507

[39] Wu A Peng Y Huang B Ding X Wang X Niu P Genome Composition and Divergence of the Novel Coronavirus (2019-nCoV) Originating in China. Cell Host Microbe (2020) 27:325–8. 10.1016/j.chom.2020.02.001 PMC7154514 32035028

[40] Baruah V Bose S Immunoinformatics-aided identification of T cell and B cell epitopes in the surface glycoprotein of 2019-nCoV. J Med Virol (2020) 92:495–500. 10.1002/jmv.25698 32022276 PMC7166505

[41] Baig AM Khaleeq A Ali U Syeda H Evidence of the COVID-19 Virus Targeting the CNS: Tissue Distribution, Host–Virus Interaction, and Proposed Neurotropic Mechanisms. ACS Chem Neurosci (2020) 11(7): 995–8. 10.1021/acschemneuro.0c00122 32167747

[42] Phan T Genetic diversity and evolution of SARS-CoV-2. Infect Genet Evol (2020) 81:104260. 10.1016/j.meegid.2020.104260 32092483 PMC7106203

[43] Beniac DR Andonov A Grudeski E Booth TF Architecture of the SARS coronavirus prefusion spike. Nat Struct Mol Biol (2006) 13:751–2. 10.1038/nsmb1123 PMC7097490 16845391

[44] Nieto-Torres JL DeDiego ML Verdiá-Báguena C Jimenez-Guardeño JM Regla-Nava JA Fernandez-Delgado R Severe Acute Respiratory Syndrome Coronavirus Envelope Protein Ion Channel Activity Promotes Virus Fitness and Pathogenesis. PLoS Pathog (2014) 10:e1004077. 10.1371/journal.ppat.1004077 24788150 PMC4006877

[45] Nal B Chan C Kien F Siu L Tse J Chu K Differential maturation and subcellular localization of severe acute respiratory syndrome coronavirus surface proteins S, M and E. J Gen Virol (2005) 86:1423–34. 10.1099/vir.0.80671-0 15831954

[46] Neuman BW Kiss G Kunding AH Bhella D Baksh MF Connelly S A structural analysis of M protein in coronavirus assembly and morphology. J Struct Biol (2011) 174:11–22. 10.1016/j.jsb.2010.11.021 21130884 PMC4486061

[47] Cui L Wang H Ji Y Yang J Xu S Huang X The Nucleocapsid Protein of Coronaviruses Acts as a Viral Suppressor of RNA Silencing in Mammalian Cells. J Virol (2015) 89:9029–43. 10.1128/jvi.01331-15 PMC4524063 26085159

[48] Chen Y Liu Q Guo D Emerging coronaviruses: Genome structure, replication, and pathogenesis. J Med Virol (2020) 92:418–23. 10.1002/jmv.25681 PMC7167049 31967327

[49] Walls AC Park Y-J Tortorici MA Wall A McGuire AT Veesler D Structure, Function, and Antigenicity of the SARS-CoV-2 Spike Glycoprotein. Cell (2020) 181(2):281–92.e6. 10.1016/j.cell.2020.02.058 PMC7102599 32155444

[50] Yan R Zhang Y Li Y Xia L Guo Y Zhou Q Structural basis for the recognition of the SARS-CoV-2 by full-length human ACE2. Science (2020) 367:1444–8. 10.1126/science.abb2762 PMC7164635 32132184

[51] Epand RM Fusion peptides and the mechanism of viral fusion. Biochim Biophys Acta Biomembr (2003) 1614:116–21. 10.1016/S0005-2736(03)00169-X 12873772

[52] Tang T Bidon M Jaimes JA Whittaker GR Daniel S Coronavirus membrane fusion mechanism offers a potential target for antiviral development. Antiviral Res (2020) 178:104792. 10.1016/j.antiviral.2020.104792 32272173 PMC7194977

[53] Vankadari N Wilce JA Emerging WuHan (COVID-19) coronavirus: glycan shield and structure prediction of spike glycoprotein and its interaction with human CD26. Emerg Microbes Infect (2020) 9:601–4. 10.1080/22221751.2020.1739565 PMC7103712 32178593

[54] Jeffers SA Tusell SM Gillim-Ross L Hemmila EM Achenbach JE Babcock GJ CD209L (L-SIGN) is a receptor for severe acute respiratory syndrome coronavirus. Proc Natl Acad Sci U S A (2004) 101:15748–53. 10.1073/pnas.0403812101 PMC524836 15496474

[55] Song W Gui M Wang X Xiang Y Cryo-EM structure of the SARS coronavirus spike glycoprotein in complex with its host cell receptor ACE2. PLoS Pathog (2018) 14:e1007236. 10.1371/journal.ppat.1007236 30102747 PMC6107290

[56] Walls AC Tortorici MA Snijder J Xiong X Bosch BJ Rey FA Tectonic conformational changes of a coronavirus spike glycoprotein promote membrane fusion. Proc Natl Acad Sci U S A (2017) 114:11157–62. 10.1073/pnas.1708727114 PMC5651768 29073020

[57] Liu S Xiao G Chen Y He Y Niu J Escalante CR Interaction between heptad repeat 1 and 2 regions in spike protein of SARS-associated coronavirus: Implications for virus fusogenic mechanism and identification of fusion inhibitors. Lancet (2004) 363:938–47. 10.1016/S0140-6736(04)15788-7 PMC7140173 15043961

[58] Millet JK Whittaker GR Physiological and molecular triggers for SARS-CoV membrane fusion and entry into host cells. Virology (2018) 517:3–8. 10.1016/j.virol.2017.12.015 29275820 PMC7112017

[59] Belouzard S Millet JK Licitra BN Whittaker GR Mechanisms of Coronavirus Cell Entry Mediated by the Viral Spike Protein. Viruses (2012) 4:1011–33. 10.3390/v4061011 PMC3397359 22816037

[60] Matsuyama S Ujike M Morikawa S Tashiro M Taguchi F Protease-mediated enhancement of severe acute respiratory syndrome coronavirus infection. Proc Natl Acad Sci U S A (2005) 102:12543–7. 10.1073/pnas.0503203102 PMC1194915 16116101

[61] Gierer S Bertram S Kaup F Wrensch F Heurich A Kramer-Kuhl A The Spike Protein of the Emerging Betacoronavirus EMC Uses a Novel Coronavirus Receptor for Entry, Can Be Activated by TMPRSS2, and Is Targeted by Neutralizing Antibodies. J Virol (2013) 87:5502–11. 10.1128/jvi.00128-13 PMC3648152 23468491

[62] Qian Z Dominguez SR Holmes KV Role of the Spike Glycoprotein of Human Middle East Respiratory Syndrome Coronavirus (MERS-CoV) in Virus Entry and Syncytia Formation. PLoS One (2013) 8:e76469. 10.1371/journal.pone.0076469 24098509 PMC3789674

[63] Shirato K Kawase M Matsuyama S Middle East Respiratory Syndrome Coronavirus Infection Mediated by the Transmembrane Serine Protease TMPRSS2. J Virol (2013) 87:12552–61. 10.1128/jvi.01890-13 PMC3838146 24027332

[64] Baric RS Yount B Subgenomic Negative-Strand RNA Function during Mouse Hepatitis Virus Infection. J Virol (2000) 74:4039–46. 10.1128/jvi.74.9.4039-4046.2000 PMC111917 10756015

[65] Masters PS The Molecular Biology of Coronaviruses. Adv Virus Res (2006) 65:193–292. 10.1016/S0065-3527(06)66005-3 PMC7112330 16877062

[66] Sawicki SG Sawicki DL Coronavirus transcription: subgenomic mouse hepatitis virus replicative intermediates function in RNA synthesis. J Virol (1990) 64:1050–6. 10.1128/jvi.64.3.1050-1056.1990 PMC249216 2154591

[67] De Wit E Van Doremalen N Falzarano D Munster VJ SARS and MERS: Recent insights into emerging coronaviruses. Nat Rev Microbiol (2016) 14:523–34. 10.1038/nrmicro.2016.81 PMC7097822 27344959

[68] Li X Geng M Peng Y Meng L Lu S Molecular immune pathogenesis and diagnosis of COVID-19. J Pharm Anal (2020) 10:102–8. 10.1016/j.jpha.2020.03.001 PMC7104082 32282863

[69] Karthik K Senthilkumar TMA Udhayavel S Raj GD Role of antibody-dependent enhancement (ADE) in the virulence of SARS-CoV-2 and its mitigation strategies for the development of vaccines and immunotherapies to counter COVID-19. Hum Vaccin Immunother (2020) 1–6:1–6. 10.1080/21645515.2020.1796425 PMC7484565 32845733

[70] Arvin AM Fink K Schmid MA Cathcart A Spreafico R Havenar-Daughton C Virgin HW. A perspective on potential antibody-dependent enhancement of SARS-CoV-2. Nature (2020) 584:353–63. 10.1038/s41586-020-2538-8 32659783

[71] Iwasaki A Yang Y The potential danger of suboptimal antibody responses in COVID-19. Nat Rev Immunol (2020) 20:339–41. 10.1038/s41577-020-0321-6 PMC7187142 32317716

[72] Ulrich H Pillat MM Tárnok A Dengue Fever,COVID-19 (SARS-CoV-2), and Antibody-Dependent Enhancement (ADE): A Perspective. Cytom Part A (2020) 97:662–7. 10.1002/cyto.a.24047 PMC7300451 32506725

[73] Kam YW Kien F Roberts A Cheung YC Lamirande EW Vogel L Antibodies against trimeric S glycoprotein protect hamsters against SARS-CoV challenge despite their capacity to mediate FcγRII-dependent entry into B cells in vitro. Vaccine (2007) 25:729–40. 10.1016/j.vaccine.2006.08.011 PMC7115629 17049691

[74] Jaume M Yip MS Cheung CY Leung HL Li PH Kien F Anti-Severe Acute Respiratory Syndrome Coronavirus Spike Antibodies Trigger Infection of Human Immune Cells via a pH- and Cysteine Protease-Independent Fc R Pathway. J Virol (2011) 85:10582–97. 10.1128/jvi.00671-11 PMC3187504 21775467

[75] Yip MS Leung NHL Cheung CY Li PH Lee HHY Daëron M Antibody-dependent infection of human macrophages by severe acute respiratory syndrome coronavirus. Virol J (2014) 11:82. 10.1186/1743-422X-11-82 24885320 PMC4018502

[76] Wang SF Tseng SP Yen CH Yang JY Tsao CH Shen CW Antibody-dependent SARS coronavirus infection is mediated by antibodies against spike proteins. Biochem Biophys Res Commun (2014) 451:208–14. 10.1016/j.bbrc.2014.07.090 PMC7092860 25073113

[77] Wan Y Shang J Sun S Tai W Chen J Geng Q Molecular Mechanism for Antibody-Dependent Enhancement of Coronavirus Entry. J Virol (2019) 94: e02015-19. 10.1128/jvi.02015-19 PMC7022351 31826992

[78] Tan W Lu Y Zhang J Wang J Dan Y Tan Z Viral Kinetics and Antibody Responses in Patients with COVID-19. medRxiv (2020), 2020.03.24.20042382. 10.1101/2020.03.24.20042382

[79] Tetro JA Is COVID-19 receiving ADE from other coronaviruses? Microbes Infect (2020) 22:72–3. 10.1016/j.micinf.2020.02.006 PMC7102551 32092539

[80] Kuba K Imai Y Rao S Gao H Guo F Guan B A crucial role of angiotensin converting enzyme 2 (ACE2) in SARS coronavirus-induced lung injury. Nat Med (2005) 11:875–9. 10.1038/nm1267 PMC7095783 16007097

[81] Rabi FA Al Zoubi MS Kasasbeh GA Salameh DM Al-Nasser AD SARS-CoV-2 and Coronavirus Disease 2019: What We Know So Far. Pathogens (2020) 9:231. 10.3390/pathogens9030231 PMC7157541 32245083

[82] Ding Y He L Zhang Q Huang Z Che X Hou J Organ distribution of severe acute respiratory syndrome(SARS) associated coronavirus(SARS-CoV) in SARS patients: implications for pathogenesis and virus transmission pathways. J Pathol (2004) 203:622–30. 10.1002/path.1560 PMC7167761 15141376

[83] Hamming I Timens W Bulthuis M Lely A Navis G van Goor H Tissue distribution of ACE2 protein, the functional receptor for SARS coronavirus. A first step in understanding SARS pathogenesis. J Pathol (2004) 203:631–7. 10.1002/path.1570 PMC7167720 15141377

[84] Gu J Gong E Zhang B Zheng J Gao Z Zhong Y Multiple organ infection and the pathogenesis of SARS. J Exp Med (2005) 202:415–24. 10.1084/jem.20050828 PMC2213088 16043521

[85] South AM Shaltout HA Washburn LK Hendricks AS Diz DI Chappell MC Fetal programming and the angiotensin-(1-7) axis: A review of the experimental and clinical data. Clin Sci (2019) 133:55–74. 10.1042/CS20171550 PMC6716381 30622158

[86] South AM Tomlinson L Edmonston D Hiremath S Sparks MA Controversies of renin–angiotensin system inhibition during the COVID-19 pandemic. Nat Rev Nephrol (2020) 16:1–3. 10.1038/s41581-020-0279-4 32246101 PMC7118703

[87] Imai Y Kuba K Rao S Huan Y Guo F Guan B Angiotensin-converting enzyme 2 protects from severe acute lung failure. Nature (2005) 436:112–6. 10.1038/nature03712 PMC7094998 16001071

[88] Sodhi CP Wohlford-Lenane C Yamaguchi Y Prindle T Fulton WB Wang S Attenuation of pulmonary ACE2 activity impairs inactivation of des-Arg 9 bradykinin/BKB1R axis and facilitates LPS-induced neutrophil infiltration. Am J Physiol Lung Cell Mol Physiol (2018) 314:17–31. 10.1152/ajplung PMC5866432 28935640

[89] Sungnak W Huang N Bécavin C Berg M Network HLB SARS-CoV-2 Entry Genes Are Most Highly Expressed in Nasal Goblet and Ciliated Cells within Human Airways (2020). Available at: http://arxiv.org/abs/2003.06122 (Accessed April 4, 2020).

[90] Puelles VG Lütgehetmann M Lindenmeyer MT Sperhake JP Wong MN Allweiss L Multiorgan and Renal Tropism of SARS-CoV-2. N Engl J Med (2020) 383:590–2. 10.1056/NEJMc2011400 PMC7240771 32402155

[91] Dhama K Patel SK Pathak M Yatoo MI Tiwari R Malik YS An update on SARS-CoV-2/COVID-19 with particular reference to its clinical pathology, pathogenesis, immunopathology and mitigation strategies. Travel Med Infect Dis (2020) 101755. 10.1016/j.tmaid.2020.101755 32479816 PMC7260597

[92] Pennisi M Lanza G Falzone L Fisicaro F Ferri R Bella R SARS-CoV-2 and the Nervous System: From Clinical Features to Molecular Mechanisms. Int J Mol Sci (2020) 21:5475. 10.3390/ijms21155475 PMC7432482 32751841

[93] Li YC Bai WZ Hashikawa T The neuroinvasive potential of SARS-CoV2 may play a role in the respiratory failure of COVID-19 patients. J Med Virol (2020) 92:552–5. 10.1002/jmv.25728 PMC7228394 32104915

[94] Chen N Zhou M Dong X Qu J Gong F Han Y Epidemiological and clinical characteristics of 99 cases of 2019 novel coronavirus pneumonia in Wuhan, China: a descriptive study. Lancet (2020) 395:507–13. 10.1016/S0140-6736(20)30211-7 PMC7135076 32007143

[95] Zhou F Yu T Du R Fan G Liu Y Liu Z Clinical course and risk factors for mortality of adult inpatients with COVID-19 in Wuhan, China: a retrospective cohort study. Lancet (2020) 395:1054–62. 10.1016/S0140-6736(20)30566-3 PMC7270627 32171076

[96] Guan W Ni Z Hu Y Liang W Ou C He J Clinical Characteristics of Coronavirus Disease 2019 in China. N Engl J Med (2020) 382(18):1708–20. 10.1056/nejmoa2002032 PMC7092819 32109013

[97] Gurwitz D Angiotensin receptor blockers as tentative SARS-CoV-2 therapeutics. Drug Dev Res (2020) 81:ddr.21656. 10.1002/ddr.21656 PMC7228359 32129518

[98] Fang L Karakiulakis G Roth M Are patients with hypertension and diabetes mellitus at increased risk for COVID-19 infection? Lancet Respir Med (2020) 8:e21. 10.1016/S2213-2600(20)30116-8 32171062 PMC7118626

[99] Chamsi-Pasha MAR Shao Z Tang WHW Angiotensin-converting enzyme 2 as a therapeutic target for heart failure. Curr Heart Fail Rep (2014) 11:58–63. 10.1007/s11897-013-0178-0 24293035 PMC3944399

[100] Li XC Zhang J Zhuo JL The vasoprotective axes of the renin-angiotensin system: Physiological relevance and therapeutic implications in cardiovascular, hypertensive and kidney diseases. Pharmacol Res (2017) 125:21–38. 10.1016/j.phrs.2017.06.005 28619367 PMC5607101

[101] Furuhashi M Moniwa N Mita T Fuseya T Ishimura S Ohno K Urinary Angiotensin-Converting Enzyme 2 in Hypertensive Patients May Be Increased by Olmesartan, an Angiotensin II Receptor Blocker. Am J Hypertens (2015) 28:15–21. 10.1093/ajh/hpu086 24842388

[102] Grover A Oberoi M A systematic review and meta-analysis to evaluate the clinical outcomes in COVID-19 patients on angiotensin-converting enzyme inhibitors or angiotensin receptor blockers. Eur Hear J Cardiovasc Pharmacother (2020) 15:pvaa064. 10.1093/ehjcvp/pvaa064 PMC7314072 32542337

[103] American Heart Association Patients taking ACE-i and ARBs who contract COVID-19 should continue treatment, unless otherwise advised by their physician | American Heart Association (2020). Available at: https://newsroom.heart.org/news/patients-taking-ace-i-and-arbs-who-contract-covid-19-should-continue-treatment-unless-otherwise-advised-by-their-physician?utm_campaign=sciencenews19-20&utm_source=science-news&utm_medium=phd-link&utm_content=phd03-21-20 (Accessed June 24, 2020).

[104] Society of Cardiology E ESC Guidance for the Diagnosis and Management of CV Disease during the COVID-19 Pandemic. European Society of Cardiology (2020).

[105] Restrepo MI Sibila O Anzueto A Pneumonia in patients with chronic obstructive pulmonary disease. Tuberc Respir Dis (Seoul) (2018) 81:187–97. 10.4046/trd.2018.0030 PMC6030662 29962118

[106] Leung JM Yang CX Tam A Shaipanich T Hackett T-L Singhera GK Early View ACE-2 Expression in the Small Airway Epithelia of Smokers and COPD Patients: Implications for COVID-19. Eur Respir J (2020) 55(5):2000688. 10.1183/13993003.00688-2020 32269089 PMC7144263

[107] Lippi G Henry BM Chronic obstructive pulmonary disease is associated with severe coronavirus disease 2019 (COVID-19). Respir Med (2020) 167: 105941. 10.1016/j.rmed.2020.105941 32421537 PMC7154502

[108] Xu Z Shi L Wang Y Zhang J Huang L Zhang C Pathological findings of COVID-19 associated with acute respiratory distress syndrome. Lancet Respir Med (2020) 8:420–2. 10.1016/S2213-2600(20)30076-X PMC7164771 32085846

[109] He F Deng Y Li W Coronavirus Disease 2019 (COVID-19): What we know? J Med Virol (2020) 92:719–25. 10.1002/jmv.25766 PMC7228340 32170865

[110] Liu K Chen Y Lin R Han K Clinical features of COVID-19 in elderly patients: A comparison with young and middle-aged patients. J Infect (2020) 80(6):14–8. 10.1016/j.jinf.2020.03.005 PMC7102640 32171866

[111] Wang D Hu B Hu C Zhu F Liu X Zhang J Clinical Characteristics of 138 Hospitalized Patients with 2019 Novel Coronavirus-Infected Pneumonia in Wuhan, China. JAMA J Am Med Assoc (2020) 323:1061–9. 10.1001/jama.2020.1585 PMC7042881 32031570

[112] Nikolich-Zugich J Knox KS Rios CT Natt B Bhattacharya D Fain MJ SARS-CoV-2 and COVID-19 in older adults: what we may expect regarding pathogenesis, immune responses, and outcomes. GeroScience (2020) 1:505–14. 10.1007/s11357-020-00186-0 PMC7145538 32274617

[113] Mao L Jin H Wang M Hu Y Chen S He Q Neurologic Manifestations of Hospitalized Patients with Coronavirus Disease 2019 in Wuhan, China. JAMA Neurol (2020) 77:683–90. 10.1001/jamaneurol.2020.1127 PMC7149362 32275288

[114] Zhou Z Kang H Li S Zhao X Understanding the neurotropic characteristics of SARS-CoV-2: from neurological manifestations of COVID-19 to potential neurotropic mechanisms. J Neurol (2020) 267:2179–84. 10.1007/s00415-020-09929-7 PMC7249973 32458193

[115] Yachou Y El Idrissi A Belapasov V Ait Benali S Neuroinvasion, neurotropic, and neuroinflammatory events of SARS-CoV-2: understanding the neurological manifestations in COVID-19 patients. Neurol Sci (2020) 41:2657–69. 10.1007/s10072-020-04575-3 PMC7385206 32725449

[116] Mao L Wang M Chen S He Q Chang J Hong C Neurological Manifestations of Hospitalized Patients with COVID-19 in Wuhan, China: a retrospective case series study JAMA Neurol (2020) 77(6):683–90. 10.1101/2020.02.22.20026500 PMC7149362 32275288

[117] Patel SK Singh R Rana J Tiwari R Natesan S Harapan H The kidney and COVID-19 patients – Important considerations. Travel Med Infect Dis (2020) 37:101831. 10.1016/j.tmaid.2020.101831 32750416 PMC7395611

[118] Naicker S Yang CW Hwang SJ Liu BC Chen JH Jha V The Novel Coronavirus 2019 epidemic and kidneys. Kidney Int (2020) 97:824–8. 10.1016/j.kint.2020.03.001 PMC7133222 32204907

[119] Pan X Xu D Zhang H Zhou W Wang Lh Cui Xg Identification of a potential mechanism of acute kidney injury during the COVID-19 outbreak: a study based on single-cell transcriptome analysis. Intens Care Med (2020) 46:1114–6. 10.1007/s00134-020-06026-1 PMC7106051 32236644

[120] Cheng Y Luo R Wang K Zhang M Wang Z Dong L Kidney impairment is associated with in-hospital death of COVID-19 patients. medRxiv (2020). 10.1101/2020.02.18.20023242 PMC7110296 32247631

[121] Chen L Li X Chen M Feng Y Xiong C The ACE2 expression in human heart indicates new potential mechanism of heart injury among patients infected with SARS-CoV-2. Cardiovasc Res (2020) 116:1097–100. 10.1093/cvr/cvaa078 PMC7184507 32227090

[122] Long B Brady WJ Koyfman A Gottlieb M Cardiovascular complications in COVID-19. Am J Emerg Med (2020) 38:1504–7. 10.1016/j.ajem.2020.04.048 PMC7165109 32317203

[123] Seah I Agrawal R Can the Coronavirus Disease 2019 (COVID-19) Affect the Eyes? A Review of Coronaviruses and Ocular Implications in Humans and Animals. Ocul Immunol Inflamm (2020) 28:391–5. 10.1080/09273948.2020.1738501 PMC7103678 32175797

[124] Xiao F Tang M Zheng X Liu Y Li X Shan H Evidence for Gastrointestinal Infection of SARS-CoV-2. Gastroenterology (2020) 158(6):1831–3. 10.1053/j.gastro.2020.02.055 PMC7130181 32142773

[125] Gu J Han B Wang J COVID-19: Gastrointestinal Manifestations and Potential Fecal–Oral Transmission. Gastroenterology (2020) 0:1518–9. 10.1053/j.gastro.2020.02.054 PMC7130192 32142785

[126] Zhang W Du RH Li B Zheng XS Yang XL Hu B Molecular and serological investigation of 2019-nCoV infected patients: implication of multiple shedding routes. Emerg Microbes Infect (2020) 9:386–9. 10.1080/22221751.2020.1729071 PMC7048229 32065057

[127] Guo D Xia J Shen Y Tong J SARS-CoV-2 may be related to conjunctivitis but not necessarily spread through the conjunctiva SARS-CoV-2 and conjunctiva. J Med Virol (2020) 92:1757–8. 10.1002/jmv.25856 PMC7262022 32275079

[128] Iba T Levy JH Levi M Thachil J Coagulopathy in COVID-19. J Thromb Haemost (2020) 18:jth.14975. 10.1111/jth.14975 PMC7323352 32558075

[129] Iba T Levy JH Connors JM Warkentin TE Thachil J Levi M The unique characteristics of COVID-19 coagulopathy. Crit Care (2020) 24:360. 10.1186/s13054-020-03077-0 32552865 PMC7301352

[130] Levi M Thachil J Iba T Levy JH Coagulation abnormalities and thrombosis in patients with COVID-19. Lancet Haematol (2020) 7:e438–40. 10.1016/S2352-3026(20)30145-9 PMC7213964 32407672

[131] Ribes A Vardon-Bounes F Mémier V Poette M Au-Duong J Garcia C Thromboembolic events and Covid-19. Adv Biol Regul (2020) 77:100735. 10.1016/j.jbior.2020.100735 32773098 PMC7833411

[132] Tang N Li D Wang X Sun Z Abnormal coagulation parameters are associated with poor prognosis in patients with novel coronavirus pneumonia. J Thromb Haemost (2020) 18:844–7. 10.1111/jth.14768 PMC7166509 32073213

[133] Dolhnikoff M Duarte-Neto AN de Almeida Monteiro RA da Silva LFF de Oliveira EP Saldiva PHN Pathological evidence of pulmonary thrombotic phenomena in severe COVID-19. J Thromb Haemost (2020) 18:1517–9. 10.1111/jth.14844 PMC7262093 32294295

[134] Kanne JP Chest CT findings in 2019 novel coronavirus (2019-NCoV) infections from Wuhan, China: Key points for the radiologist. Radiology (2020) 295:16–7. 10.1148/radiol.2020200241 PMC7233362 32017662

[135] Wang D Hu B Hu C Zhu F Liu X Zhang J Clinical Characteristics of 138 Hospitalized Patients With 2019 Novel Coronavirus–Infected Pneumonia in Wuhan, China. JAMA (2020) 323:1061. 10.1001/jama.2020.1585 32031570 PMC7042881

[136] Chung M Bernheim A Mei X Zhang N Huang M Zeng X CT imaging features of 2019 novel coronavirus (2019-NCoV). Radiology (2020) 295:202–7. 10.1148/radiol.2020200230 PMC7194022 32017661

[137] Wu J Wu X Zeng W Guo D Fang Z Chen L Chest CT Findings in Patients with Corona Virus Disease 2019 and its Relationship with Clinical Features. Invest Radiol (2020) 1:257–61. 10.1097/rli.0000000000000670 PMC7147284 32091414

[138] Song F Shi N Shan F Zhang Z Shen J Lu H Emerging 2019 Novel Coronavirus (2019-nCoV) Pneumonia. Radiology (2020) 295:210–7. 10.1148/radiol.2020200274 PMC7233366 32027573

[139] Ye Z Zhang Y Wang Y Huang Z Song B Chest CT manifestations of new coronavirus disease 2019 (COVID-19): a pictorial review. Eur Radiol (2020) 30:1–9. 10.1007/s00330-020-06801-0 32193638 PMC7088323

[140] Xie X Zhong Z Zhao W Zheng C Wang F Liu J Chest CT for Typical 2019-nCoV Pneumonia: Relationship to Negative RT-PCR Testing. Radiology (2020) 296:200343. 10.1148/radiol.2020200343 PMC7233363 32049601

[141] Wang BX Fish EN Global virus outbreaks: Interferons as 1st responders. Semin Immunol (2019) 43:101300. 10.1016/j.smim.2019.101300 31771760 PMC7128104

[142] Lim KH Staudt LM Toll-Like receptor signaling. Cold Spring Harb Perspect Biol (2013) 5:a011247. 10.1101/cshperspect.a011247 23284045 PMC3579400

[143] Li Y Chen M Cao H Zhu Y Zheng J Zhou H Extraordinary GU-rich single-strand RNA identified from SARS coronavirus contributes an excessive innate immune response. Microbes Infect (2013) 15:88–95. 10.1016/j.micinf.2012.10.008 23123977 PMC7110875

[144] Ahmadpoor P Rostaing L Why the immune system fails to mount an adaptive immune response to a Covid -19 infection. Transpl Int (2020) 33: 824–5. 10.1111/tri.13611 32236983

[145] Del Valle DM Kim-Schulze S Huang H-H Beckmann ND Nirenberg S Wang B An inflammatory cytokine signature predicts COVID-19 severity and survival. Nat Med (2020) 26:1–8. 10.1038/s41591-020-1051-9 32839624 PMC7869028

[146] Salem ESB Vonberg AD Borra VJ Gill RK Nakamura T RNAs and RNA-Binding Proteins in Immuno-Metabolic Homeostasis and Diseases. Front Cardiovasc Med (2019) 6:106. 10.3389/fcvm.2019.00106 31482095 PMC6710452

[147] Cervantes-Barragan L Züst R Weber F Spiegel M Lang KS Akira S Control of coronavirus infection through plasmacytoid dendritic-cell- derived type I interferon. Blood (2007) 109:1131–7. 10.1182/blood-2006-05-023770 PMC8254533 16985170

[148] Li J-Y Liao C-H Wang Q Tan Y-J Luo R Qiu Y The ORF6, ORF8 and nucleocapsid proteins of SARS-CoV-2 inhibit type I interferon signaling pathway. Virus Res (2020) 286:198074. 10.1016/j.virusres.2020.198074 32589897 PMC7309931

[149] Trouillet-Assant S Viel S Gaymard A Pons S Richard JC Perret M Type I IFN immunoprofiling in COVID-19 patients. J Allergy Clin Immunol (2020) 146(1):P206–8. 10.1016/j.jaci.2020.04.029 PMC7189845 32360285

[150] Zhou Z Ren L Zhang L Jin Q Li M Correspondence JW Heightened Innate Immune Responses in the Respiratory Tract of COVID-19 Patients. Cell Host Microbe (2020) 27:883–90. 10.1016/j.chom.2020.04.017 PMC7196896 32407669

[151] Major J Crotta S Llorian M McCabe TM Gad HH Priestnall SL Type I and III interferons disrupt lung epithelial repair during recovery from viral infection. Science (80 ) (2020) 369:eabc2061. 10.1126/science.abc2061 PMC7292500 32527928

[152] Channappanavar R Fehr AR Zheng J Wohlford-Lenane C Abrahante JE Mack M IFN-I response timing relative to virus replication determines MERS coronavirus infection outcomes. J Clin Invest (2019) 129:3625–39. 10.1172/JCI126363 PMC6715373 31355779

[153] Qin C Zhou L Hu Z Zhang S Yang S Tao Y Dysregulation of immune response in patients with COVID-19 in Wuhan, China. Clin Infect Dis (2020) 71(15):762–8. 10.1093/cid/ciaa248 32161940 PMC7108125

[154] Chen Y Diao B Wang C Chen X Liu Y Ning L Reduction and Functional Exhaustion of T Cells in Patients with Coronavirus Disease 2019 (COVID-19). Front Immunol (2020) 11:827. 10.3389/FIMMU.2020.00827 32425950 PMC7205903

[155] Russell B Moss C George G Santaolalla A Cope A Papa S Associations between immune-suppressive and stimulating drugs and novel COVID-19—a systematic review of current evidence. Ecancermedicalscience (2020) 14:1022. 10.3332/ecancer.2020.1022 32256705 PMC7105343

[156] Chi Y Ge Y Wu B Zhang W Wu T Wen T Serum Cytokine and Chemokine Profile in Relation to the Severity of Coronavirus Disease 2019 in China. J Infect Dis (2020) 222:746–54. 10.1093/infdis/jiaa363 PMC7337752 32563194

[157] Neidleman J Luo X Frouard J Ghosn E Lee S Roan NR SARS-CoV-2-Specific T Cells Exhibit Phenotypic Features of Helper Function, Lack of Terminal Differentiation, and High Proliferation Potential. Cell Rep Med (2020) 1(6):100081. 10.1016/j.xcrm.2020.100081 32839763 PMC7437502

[158] Van Dyk LF Chung KF Di PR Swartz TH Freeman TL Targeting the NLRP3 Inflammasome in Severe COVID-19. Front Immunol www.frontiersin.org (2020) 1:1518. 10.3389/fimmu.2020.01518 PMC7324760 32655582

[159] Cauchois R Koubi M Delarbre D Manet C Carvelli J Blasco VB Early IL-1 receptor blockade in severe inflammatory respiratory failure complicating COVID-19. Proc Natl Acad Sci U S A (2020) 117:18951–3. 10.1073/pnas.2009017117 PMC7430998 32699149

[160] Silva TF Concato VM Tomiotto-Pellissier F Gonçalves MD Bortoleti BT da S Tavares ER Reactivation of Cytomegalovirus Increases Nitric Oxide and IL-10 Levels in Sepsis and is Associated with Changes in Renal Parameters and Worse Clinical Outcome. Sci Rep (2019) 9:1–9. 10.1038/s41598-019-45390-x 31227794 PMC6588619

[161] Lin GL McGinley JP Drysdale SB Pollard AJ Epidemiology and Immune Pathogenesis of Viral Sepsis. Front Immunol (2018) 9:2147. 10.3389/fimmu.2018.02147 30319615 PMC6170629

[162] Ma Z Ni G Damania B Innate Sensing of DNA Virus Genomes. Annu Rev Virol (2018) 5:341–62. 10.1146/annurev-virology-092917-043244 PMC6443256 30265633

[163] Kumar BV Connors TJ Farber DL Human T Cell Development, Localization, and Function throughout Life. Immunity (2018) 48:202–13. 10.1016/j.immuni.2018.01.007 PMC5826622 29466753

[164] Zuo Y Yalavarthi S Shi H Gockman K Zuo M Madison JA Neutrophil extracellular traps in COVID-19. JCI Insight (2020) 5:e138999. 10.1172/jci.insight.138999 PMC7308057 32329756

[165] Brinkmann V Reichard U Goosmann C Fauler B Uhlemann Y Weiss DS Neutrophil Extracellular Traps Kill Bacteria. Sci (80 ) (2004) 303:1532–5. 10.1126/science.1092385 15001782

[166] Warnatsch A Ioannou M Wang Q Papayannopoulos V Neutrophil extracellular traps license macrophages for cytokine production in atherosclerosis. Sci (80 ) (2015) 349:316–20. 10.1126/science.aaa8064 PMC4854322 26185250

[167] Tanaka K Koike Y Shimura T Okigami M Ide S Toiyama Y In Vivo Characterization of Neutrophil Extracellular Traps in Various Organs of a Murine Sepsis Model. PLoS One (2014) 9:e111888. 10.1371/journal.pone.0111888 25372699 PMC4221155

[168] Narasaraju T Yang E Samy RP Ng HH Poh WP Liew AA Excessive neutrophils and neutrophil extracellular traps contribute to acute lung injury of influenza pneumonitis. Am J Pathol (2011) 179:199–210. 10.1016/j.ajpath.2011.03.013 21703402 PMC3123873

[169] Lefrançais E Mallavia B Zhuo H Calfee CS Looney MR Maladaptive role of neutrophil extracellular traps in pathogen-induced lung injury. JCI Insight (2018) 3:e98178. 10.1172/jci.insight.98178 PMC5821185 29415887

[170] Zhou Y Fu B Zheng X Wang D Zhao C Qi Y Pathogenic T cells and inflammatory monocytes incite inflammatory storm in severe COVID-19 patients | National Science Review | Oxford Academic(2020). Available at: https://academic.oup.com/nsr/advance-article/doi/10.1093/nsr/nwaa041/5804736 (Accessed April 5, 2020). 10.1093/nsr/nwaa041 PMC7108005 34676125

[171] Kapellos TS Bonaguro L Gemünd I Reusch N Saglam A Hinkley ER Human monocyte subsets and phenotypes in major chronic inflammatory diseases. Front Immunol (2019) 10:2035. 10.3389/fimmu.2019.02035 31543877 PMC6728754

[172] Dutertre CA Amraoui S DeRosa A Jourdain JP Vimeux L Goguet M Pivotal role of M-DC8+ monocytes from viremic HIV-infected patients in TNFα overproduction in response to microbial products. Blood (2012) 120:2259–68. 10.1182/blood-2012-03-418681 22802339

[173] Liao M Liu Y Yuan J Wen Y Xu G Zhao J Single-cell landscape of bronchoalveolar immune cells in patients with COVID-19. Nat Med (2020) 26:842–4. 10.1038/s41591-020-0901-9 32398875

[174] Huang H Wang S Jiang T Fan R Zhang Z Mu J High levels of circulating GM-CSF+CD4+ T cells are predictive of poor outcomes in sepsis patients: a prospective cohort study. Cell Mol Immunol (2019) 16:602–10. 10.1038/s41423-018-0164-2 PMC6804788 30327490

[175] Croxford AL Lanzinger M Hartmann FJ Schreiner B Mair F Pelczar P The Cytokine GM-CSF Drives the Inflammatory Signature of CCR2+ Monocytes and Licenses Autoimmunity. Immunity (2015) 43:502–14. 10.1016/j.immuni.2015.08.010 26341401

[176] Giamarellos-Bourboulis EJ Netea MG Rovina N Akinosoglou K Antoniadou A Antonakos N Complex Immune Dysregulation in COVID-19 Patients with Severe Respiratory Failure. Cell Host Microbe (2020) 27:992–1000. 10.1016/j.chom.2020.04.009 32320677 PMC7172841

[177] Spinetti T Hirzel C Fux M Walti LN Schober P Stueber F Reduced monocytic HLA-DR expression indicates immunosuppression in critically ill COVID-19 patients. Anesth Analg (2020) 131(4):993–9. 10.1213/ane.0000000000005044 PMC7288784 32925314

[178] Yang D Chu H Hou Y Chai Y Shuai H Chak A Attenuated Interferon and Proinflammatory Response in SARS-CoV-2-Infected Human Dendritic Cells Is Associated With Viral Antagonism of STAT1 Phosphorylation. J Infect Dis (2020) XX:1–12. 10.1093/infdis/jiaa356 PMC7337793 32563187

[179] Luft T Pang KC Thomas E Hertzog P Hart DN Trapani J Type I IFNs enhance the terminal differentiation of dendritic cells(1998). 9712065

[180] Dauer M IFN- promotes definitive maturation of dendritic cells generated by short-term culture of monocytes with GM-CSF and IL-4. J Leukoc Biol (2006) 80:278–86. 10.1189/jlb.1005592 16769767

[181] Gautier G Humbert M Deauvieau F Scuiller M Hiscott J Bates EEM A type I interferon autocrine-paracrine loop is involved in Toll-like receptor-induced interleukin-12p70 secretion by dendritic cells. J Exp Med (2005) 201:1435–46. 10.1084/jem.20041964 PMC2213193 15851485

[182] Grant EJ Nüssing S Sant S Clemens EB Kedzierska K The role of CD27 in anti-viral T-cell immunity. Curr Opin Virol (2017) 22:77–88. 10.1016/j.coviro.2016.12.001 28086150

[183] Ruan Q Yang K Wang W Jiang L Song J Clinical predictors of mortality due to COVID-19 based on an analysis of data of 150 patients from Wuhan, China. Intens Care Med (2020) 22:77–88. 10.1007/s00134-020-05991-x PMC7080116 32125452

[184] Cao X COVID-19: immunopathology and its implications for therapy. Nat Rev Immunol (2020) 20:269–70. 10.1038/s41577-020-0308-3 PMC7143200 32273594

[185] Van Braeckel E Desombere I Clement F Vandekerckhove L Verhofstede C Vogelaers D Polyfunctional CD4(+) T cell responses in HIV-1-infected viral controllers compared with those in healthy recipients of an adjuvanted polyprotein HIV-1 vaccine. Vaccine (2013) 31:3739–46. 10.1016/j.vaccine.2013.05.021 23707169

[186] Han Q Bagheri N Bradshaw EM Hafler DA Lauffenburger DA Love JC Polyfunctional responses by human T cells result from sequential release of cytokines. Proc Natl Acad Sci U S A (2012) 109:1607–12. 10.1073/pnas.1117194109 PMC3277116 22160692

[187] Li CK Wu H Yan H Ma S Wang L Zhang M T Cell Responses to Whole SARS Coronavirus in Humans. J Immunol (2008) 181:5490–500. 10.4049/jimmunol.181.8.5490 PMC2683413 18832706

[188] Zhang JY Wang XM Xing X Xu Z Zhang C Song JW Single-cell landscape of immunological responses in patients with COVID-19. Nat Immunol (2020) 21:1107–18. 10.1038/s41590-020-0762-x 32788748

[189] Saeidi A Zandi K Cheok YY Saeidi H Wong WF Lee CYQ Shankar EM. T-Cell Exhaustion in Chronic Infections: Reversing the State of Exhaustion and Reinvigorating Optimal Protective Immune Responses. Front Immunol (2018) 9:2569. 10.3389/fimmu.2018.02569 30473697 PMC6237934

[190] Wing JB Tay C Sakaguchi S Control of regulatory T cells by co-signal molecules. In: Azuma M Yagita H, editors. Advances in Experimental Medicine and Biology, vol. 1189 (Singapore: Springer) (2019). p. 179–210. 10.1007/978-981-32-9717-3_7 31758535

[191] Sakaguchi S Miyara M Costantino CM Hafler DA FOXP3 + regulatory T cells in the human immune system. Nat Rev Immunol (2010) 10:490–500. 10.1038/nri2785 20559327

[192] Chen G Wu D Guo W Cao Y Huang D Wang H Clinical and immunological features of severe and moderate coronavirus disease 2019. J Clin Invest (2020) 130:2620–9. 10.1172/JCI137244 PMC7190990 32217835

[193] Wang F Hou H Luo Y Tang G Wu S Huang M The laboratory tests and host immunity of COVID-19 patients with different severity of illness. JCI Insight (2020) 5:5. 10.1172/JCI.INSIGHT.137799 PMC7259533 32324595

[194] Kamiya T Seow SV Wong D Robinson M Campana D Blocking expression of inhibitory receptor NKG2A overcomes tumor resistance to NK cells. J Clin Invest (2019) 129:2094–106. 10.1172/JCI123955 PMC6486333 30860984

[195] Wei H Li F Wei H Gao Y Xu L Yin W Blocking the natural killer cell inhibitory receptor NKG2A increases activity of human natural killer cells and clears hepatitis B virus infection in mice. Gastroenterology (2013) 144:392–401. 10.1053/j.gastro.2012.10.039 23103614

[196] Joller N Kuchroo VK Tim-3, Lag-3, and TIGIT. In: Current Topics in Microbiology and Immunology. (Cham: Springer Verlag) (2017). p. 127–56. 10.1007/82_2017_62 PMC5902028 28900677

[197] Guo L Ren L Yang S Xiao M Chang D Yang F Profiling Early Humoral Response to Diagnose Novel Coronavirus Disease (COVID-19). Clin Infect Dis (2020) 71(15):778–85. 10.1093/cid/ciaa310 PMC7184472 32198501

[198] Bannard O Cyster JG Germinal centers: programmed for affinity maturation and antibody diversification. Curr Opin Immunol (2017) 45:21–30. 10.1016/j.coi.2016.12.004 28088708

[199] Mesin L Ersching J Victora GD Germinal Center B Cell Dynamics. Immunity (2016) 45:471–82. 10.1016/j.immuni.2016.09.001 PMC5123673 27653600

[200] Chen X Pan Z Yue S Yu F Zhang J Yang Y Disease severity dictates SARS-CoV-2-specific neutralizing antibody responses in COVID-19. Signal Transd Target Ther (2020) 5:180. 10.1038/s41392-020-00301-9 PMC7464057 32879307

[201] Yang L Gou J Gao J Huang L Zhu Z Ji S Immune characteristics of severe and critical COVID-19 patients. Signal Transd Target Ther (2020) 5:179. 10.1038/s41392-020-00296-3 PMC7456639 32868756

[202] Zhang F Gan R Zhen Z Hu X Li X Zhou F Adaptive immune responses to SARS-CoV-2 infection in severe versus mild individuals. Signal Transd Target Ther (2020) 5:1–11. 10.1038/s41392-020-00263-y PMC7426596 32796814

[203] Chen X Li R Pan Z Qian C Yang Y You R Human monoclonal antibodies block the binding of SARS-CoV-2 spike protein to angiotensin converting enzyme 2 receptor. Cell Mol Immunol (2020) 17:647–9. 10.1038/s41423-020-0426-7 PMC7167496 32313207

[204] Shen C Wang Z Zhao F Yang Y Li J Yuan J Treatment of 5 Critically Ill Patients With COVID-19 With Convalescent Plasma. JAMA (2020) 323 (16):1582–9. 10.1001/jama.2020.4783 PMC7101507 32219428

[205] Jayawardena R Sooriyaarachchi P Chourdakis M Jeewandara C Ranasinghe P Enhancing immunity in viral infections, with special emphasis on COVID-19: A review. Diabetes Metab Syndr Clin Res Rev (2020) 14:367–82. 10.1016/j.dsx.2020.04.015 PMC7161532 32334392

[206] Patel N Penkert RR Jones BG Sealy RE Surman SL Sun Y Baseline serum Vitamin A and D levels determine benefit of oral Vitamin A&D supplements to humoral immune responses following pediatric influenza vaccination. Viruses (2019) 11:907. 10.3390/v11100907 PMC6832482 31575021

[207] Ivory K Prieto E Spinks C Armah CN Goldson AJ Dainty JR Selenium supplementation has beneficial and detrimental effects on immunity to influenza vaccine in older adults. Clin Nutr (2017) 36:407–15. 10.1016/j.clnu.2015.12.003 PMC5381341 26803169

[208] Iovino L Mazziotta F Carulli G Guerrini F Morganti R Mazzotti V High-dose zinc oral supplementation after stem cell transplantation causes an increase of TRECs and CD4+ naïve lymphocytes and prevents TTV reactivation. Leuk Res (2018) 70:20–4. 10.1016/j.leukres.2018.04.016 29747074

[209] Grant WB Lahore H McDonnell SL Baggerly CA French CB Aliano JL Evidence that vitamin d supplementation could reduce risk of influenza and covid-19 infections and deaths. Nutrients (2020) 12:988. 10.3390/nu12040988 PMC7231123 32252338

[210] Zdrenghea MT Makrinioti H Bagacean C Bush A Johnston SL Stanciu LA Vitamin D modulation of innate immune responses to respiratory viral infections. Rev Med Virol (2017) 27:e1909. 10.1002/rmv.1909 27714929

[211] Monlezun DJ Bittner EA Christopher KB Camargo CA Quraishi SA Vitamin D status and acute respiratory infection: Cross sectional results from the United States national health and nutrition examination survey, 2001-2006. Nutrients (2015) 7:1933–44. 10.3390/nu7031933 PMC4377891 25781219

[212] Hastie CE Mackay DF Ho F Celis-Morales CA Katikireddi SV Niedzwiedz CL Vitamin D concentrations and COVID-19 infection in UK Biobank. Diabetes Metab Syndr Clin Res Rev (2020) 14:561–5. 10.1016/j.dsx.2020.04.050 PMC7204679 32413819

[213] Douglas RM Chalker EB Treacy B Vitamin C for the common cold. Cochrane Database Syst Rev (1988) (2):CD000980. 10.1002/14651858.CD000980 10796569

[214] Moriguchi S Muraga M Vitamin E and immunity. Vitam Horm (2000) 59:305–36. 10.1016/s0083-6729(00)59011-6 10714244

[215] Hemilä H Kaprio J Vitamin E supplementation and pneumonia risk in males who initiated smoking at an early age: Effect modification by body weight and dietary vitamin C. Nutr J (2008) 7:33. 10.1186/1475-2891-7-33 19019244 PMC2603040

[216] Meydani SN Leka LS Fine BC Dallal GE Keusch GT Singh MF and respiratory tract infections in elderly nursing home residents: A randomized controlled trial. J Am Med Assoc (2004) 292:828–36. 10.1001/jama.292.7.828 PMC2377357 15315997

[217] AminJafari A Ghasemi S The possible of immunotherapy for COVID-19: A systematic review. Int Immunopharmacol (2020) 83:106455. 10.1016/j.intimp.2020.106455 32272396 PMC7128194

[218] Shanmugaraj B Siriwattananon K Wangkanont K Phoolcharoen W Perspectives on monoclonal antibody therapy as potential therapeutic intervention for Coronavirus disease-19 (COVID-19). Asian Pacific J Allergy Immunol (2020) 38:10–18. 10.12932/AP-200220-0773 32134278

[219] Dhama K Sharun K Tiwari R Dadar M Malik YS Singh KP COVID-19, an emerging coronavirus infection: advances and prospects in designing and developing vaccines, immunotherapeutics, and therapeutics. Hum Vaccines Immunother (2020) 16:1232–8. 10.1080/21645515.2020.1735227 PMC7103671 32186952

[220] Owji H Negahdaripour M Hajighahramani N Immunotherapeutic approaches to curtail COVID-19. Int Immunopharmacol (2020) 88:106924. 10.1016/j.intimp.2020.106924 32877828 PMC7441891

[221] Elfiky AA Mahdy SM Elshemey WM Quantitative structure-activity relationship and molecular docking revealed a potency of anti-hepatitis C virus drugs against human corona viruses. J Med Virol (2017) 89:1040–7. 10.1002/jmv.24736 PMC7167072 27864902

[222] Jiang S Hillyer C Du L Neutralizing Antibodies against SARS-CoV-2 and Other Human Coronaviruses. Trends Immunol (2020) 41(5):P355–9. 10.1016/j.it.2020.03.007 PMC7129017 32249063

[223] Teixeira da Silva JA Convalescent plasma: A possible treatment of COVID-19 in India. Med J Armed Forces India (2020) 76(2):236–7. 10.1016/j.mjafi.2020.04.006 PMC7158785 32296259

[224] Van Erp EA Luytjes W Ferwerda G Van Kasteren PB Fc-mediated antibody effector functions during respiratory syncytial virus infection and disease. Front Immunol (2019) 10:548. 10.3389/fimmu.2019.00548 30967872 PMC6438959

[225] Rabaan AA Al-Ahmed SH Sah R Tiwari R Yatoo MI Patel SK SARS-CoV-2/COVID-19 and advances in developing potential therapeutics and vaccines to counter this emerging pandemic. Ann Clin Microbiol Antimicrob (2020) 19:40. 10.1186/s12941-020-00384-w 32878641 PMC7464065

[226] Wrapp D Wang N Corbett KS Goldsmith JA Hsieh C-L Abiona O Cryo-EM structure of the 2019-nCoV spike in the prefusion conformation (2019). Available at: http://science.sciencemag.org/ (Accessed September 8, 2020). 10.1126/science.abb2507 PMC7164637 32075877

[227] COVID-19 Neutralizing Human Monoclonal Antibodies Against SARS-Cov-2 - Full Text View - ClinicalTrials.gov. Available at: https://clinicaltrials.gov/ct2/show/NCT04354766?term=monoclonal+antibody&cond=COVID-19&draw=2&rank=1 (Accessed April 28, 2020).

[228] Tolerability,Safety,Pharmacokinetic Profile and Immunogenicity of a Recombinant Humanized Anti-SARS-CoV-2 Monoclonal Antibody (JS016) for Injection in Chinese Health Subjects - Full Text View - ClinicalTrials.gov . Available at: https://clinicaltrials.gov/ct2/show/NCT04441918?term=monoclonal+antibody&cond=COVID-19&draw=2&rank=1 (Accessed July 9, 2020).

[229] Safety, Tolerability, and Efficacy of Anti-Spike (S) SARS-CoV-2 Monoclonal Antibodies for the Treatment of Ambulatory Adult Patients With COVID-19 - Full Text View - ClinicalTrials.gov. Available at: https://clinicaltrials.gov/ct2/show/NCT04425629?term=monoclonal+antibody&cond=COVID-19&draw=2&rank=2 (Accessed July 9, 2020).

[230] Safety, Tolerability, and Efficacy of Anti-Spike (S) SARS-CoV-2 Monoclonal Antibodies for Hospitalized Adult Patients With COVID-19 - Full Text View - ClinicalTrials.gov. Available at: https://clinicaltrials.gov/ct2/show/NCT04426695?term=monoclonal+antibody&cond=COVID-19&draw=2&rank=3 (Accessed July 9, 2020).

[231] Safety, Tolerability and Pharmacokinetics of SCTA01, an Anti-SARS-CoV-2 Monoclonal Antibody, in Healthy Chinese Subjects - Full Text View - ClinicalTrials.gov. Available at: https://clinicaltrials.gov/ct2/show/NCT04483375 (Accessed September 10, 2020).

[232] Safety of TY027 a Treatment for COVID-19, in Humans - Full Text View - ClinicalTrials.gov. Available at: https://clinicaltrials.gov/ct2/show/NCT04429529?term=monoclonal+antibody&cond=COVID-19&draw=2 (Accessed July 9, 2020).

[233] VIR-7831 for the Early Treatment of COVID-19 in Outpatients - Full Text View - ClinicalTrials.gov. Available at: https://clinicaltrials.gov/ct2/show/NCT04545060 (Accessed September 10, 2020).

[234] Study Assessing the Efficacy and Safety of Anti-Spike SARS CoV-2 Monoclonal Antibodies for Prevention of SARS CoV-2 Infection Asymptomatic in Healthy Adults Who Are Household Contacts to an Individual With a Positive SARS-CoV-2 RT-PCR Assay - Full Text View - ClinicalTrials.gov Available at: https://clinicaltrials.gov/ct2/show/NCT04452318 (Accessed September 10, 2020).

[235] Study to Evaluate STI-1499 (COVI-GUARD) in Patients With Moderate COVID-19 - Full Text View - ClinicalTrials.gov. Available at: https://clinicaltrials.gov/ct2/show/NCT04454398 (Accessed September 10, 2020).

[236] Luo P Liu Y Qiu L Liu X Liu D Li J Tocilizumab treatment in COVID-19: A single center experience. J Med Virol (2020) 92:814–8. 10.1002/jmv.25801 PMC7262125 32253759

[237] Xu X Han M Li T Sun W Wang D Fu B Effective treatment of severe COVID-19 patients with tocilizumab. Proc Natl Acad Sci U S A (2020) 117:10970–5. 10.1073/pnas.2005615117 PMC7245089 32350134

[238] Melody M Nelson J Hastings J Propst J Smerina M Mendez J Case report: use of lenzilumab and tocilizumab for the treatment of coronavirus disease 2019. Immunotherapy (2020) 12(15):1121–6. 10.2217/imt-2020-0136 PMC7319491 32546029

[239] Jordan SC Zakowski P Tran HP Smith EA Gaultier C Marks G Compassionate Use of Tocilizumab for Treatment of SARS-CoV-2 Pneumonia. Clin Infect Dis (2020). 10.1093/cid/ciaa812/5861638 PMC7337689 32575124

[240] Campochiaro C Della-Torre E Cavalli G De Luca G Ripa M Boffini N Efficacy and safety of tocilizumab in severe COVID-19 patients: a single-centre retrospective cohort study. Eur J Intern Med (2020) 76:43–9. 10.1016/j.ejim.2020.05.021 PMC7242960 32482597

[241] Morena V Milazzo L Oreni L Bestetti G Fossali T Bassoli C Off-label use of tocilizumab for the treatment of SARS-CoV-2 pneumonia in Milan, Italy. Eur J Intern Med (2020) 76:36–42. 10.1016/j.ejim.2020.05.011 32448770 PMC7241995

[242] Toniati P Piva S Cattalini M Garrafa E Regola F Castelli F Tocilizumab for the treatment of severe COVID-19 pneumonia with hyperinflammatory syndrome and acute respiratory failure: A single center study of 100 patients in Brescia, Italy. Autoimmun Rev (2020) 19:102568. 10.1016/j.autrev.2020.102568 32376398 PMC7252115

[243] Khiali S Khani E Entezari-Maleki T A Comprehensive Review on Tocilizumab in COVID-19 Acute Respiratory Distress Syndrome. J Clin Pharmacol (2020) 60(9):1131–46. 10.1002/jcph.1693 PMC7323169 32557541

[244] Scherger S Henao-Martínez A Franco-Paredes C Shapiro L Rethinking interleukin-6 blockade for treatment of COVID-19. Med Hypotheses (2020) 144:110053. 10.1016/j.mehy.2020.110053 32758889 PMC7320867

[245] Zhang Y Zhong Y Pan L Dong J Treat 2019 novel coronavirus (COVID-19) with IL-6 inhibitor: Are we already that far? Drug Discovery Ther (2020) 14:100–2. 10.5582/ddt.2020.03006 32378647

[246] Crisafulli S Isgrò V La Corte L Atzeni F Trifirò G Potential Role of Anti-interleukin (IL)-6 Drugs in the Treatment of COVID-19: Rationale, Clinical Evidence and Risks. BioDrugs (2020) 34:415–22. 10.1007/s40259-020-00430-1 PMC7299248 32557214

[247] Jamilloux Y Henry T Belot A Viel S Fauter M El Jammal T Should we stimulate or suppress immune responses in COVID-19? Cytokine and anti-cytokine interventions. Autoimmun Rev (2020) 19:102567. 10.1016/j.autrev.2020.102567 32376392 PMC7196557

[248] Cavalli G De Luca G Campochiaro C Della-Torre E Ripa M Canetti D Interleukin-1 blockade with high-dose anakinra in patients with COVID-19, acute respiratory distress syndrome, and hyperinflammation: a retrospective cohort study. Lancet Rheumatol (2020) 2:325–31. 10.1016/S2665-9913(20)30127-2 PMC7252085 32501454

[249] Giudice V Pagliano P Vatrella A Masullo A Poto S Polverino BM Combination of Ruxolitinib and Eculizumab for Treatment of Severe SARS-CoV-2-Related Acute Respiratory Distress Syndrome: A Controlled Study. Front Pharmacol (2020) 11:857. 10.3389/fphar.2020.00857 32581810 PMC7291857

[250] Magro G COVID-19: Review on latest available drugs and therapies against SARS-CoV-2. Coagulation and inflammation cross-talking. Virus Res (2020) 286:198070. 10.1016/j.virusres.2020.198070 32569708 PMC7305708

[251] Roschewski M Lionakis MS Sharman JP Roswarski J Goy A Monticelli MA Inhibition of Bruton tyrosine kinase in patients with severe COVID-19. Sci Immunol (2020) 5:eabd0110. 10.1126/sciimmunol.abd0110 32503877 PMC7274761

[252] Wilkinson T Dixon R Page C Carroll M Griffiths G Ho LP ACCORD: A Multicentre, Seamless, Phase 2 Adaptive Randomisation Platform Study to Assess the Efficacy and Safety of Multiple Candidate Agents for the Treatment of COVID-19 in Hospitalised Patients: A structured summary of a study protocol for a randomised controlled trial. Trials (2020) 21:691. 10.1186/s13063-020-04584-9 32736596 PMC7393340

[253] Crizanlizumab for Treating COVID-19 Vasculopathy - Full Text View - ClinicalTrials.gov. Available at: https://clinicaltrials.gov/ct2/show/NCT04435184?term=monoclonal+antibody&cond=COVID-19&draw=2 (Accessed July 9, 2020).

[254] Treatment With CSL312 in Adults With Coronavirus Disease 2019 (COVID−19) - Full Text View - ClinicalTrials.gov . Available at: https://clinicaltrials.gov/ct2/show/NCT04409509?term=monoclonal+antibody&cond=COVID-19&draw=2 (Accessed July 9, 2020).

[255] Efficiency and Security of NIVOLUMAB Therapy in Obese Individuals With COVID-19(COrona VIrus Disease) Infection - Full Text View - ClinicalTrials.gov. Available at: https://clinicaltrials.gov/ct2/show/NCT04413838?term=monoclonal+antibody&cond=COVID-19&draw=2 (Accessed July 9, 2020).

[256] Immunoregulatory Therapy for 2019-nCoV - Full Text View - ClinicalTrials.gov. Available at: https://clinicaltrials.gov/ct2/show/NCT04268537 (Accessed September 10, 2020).

[257] COVID-19: A Pilot Study of Adaptive Immunity and Anti-PD1 - Full Text View - ClinicalTrials.gov . Available at: https://clinicaltrials.gov/ct2/show/NCT04356508?term=pd1&cond=Covid19&draw=2&rank=2 (Accessed September 10, 2020).

[258] Syal K COVID-19: Herd Immunity and Convalescent Plasma Transfer Therapy. J Med Virol (2020) 92:1380–2. 10.1002/jmv.25870 PMC7262166 32281679

[259] Zhang B Liu S Tan T Huang W Dong Y Chen L Treatment with convalescent plasma for critically ill patients with SARS-CoV-2 infection. Chest (2020) 158(1):p9–13. 10.1016/j.chest.2020.03.039 PMC7195335 32243945

[260] Zhai P Ding Y Wu X Long J Zhong Y Li Y The epidemiology, diagnosis and treatment of COVID-19. Int J Antimicrob Agents (2020) 55(5):105955. 10.1016/j.ijantimicag.2020.105955 32234468 PMC7138178

[261] Bloch EM Shoham S Casadevall A Sachais BS Shaz B Winters JL Deployment of convalescent plasma for the prevention and treatment of COVID-19. J Clin Invest (2020) 130(6):2757–65. 10.1172/JCI138745 PMC7259988 32254064

[262] Tiberghien P de Lambalerie X Morel P Gallian P Lacombe K Yazdanpanah Y Collecting and evaluating convalescent plasma for COVID-19 treatment: why and how. Vox Sang (2020) 115 (6):488–94. 10.1111/vox.12926 32240545

[263] Cheng Y Wong R Soo YOY Wong WS Lee CK Ng MHL Use of convalescent plasma therapy in SARS patients in Hong Kong. Eur J Clin Microbiol Infect Dis (2005) 24:44–6. 10.1007/s10096-004-1271-9 PMC7088355 15616839

[264] Ko J-H Seok H Cho SY Ha YE Baek JY Kim SH Challenges of convalescent plasma infusion therapy in Middle East respiratory coronavirus infection: a single centre experience. Antivir Ther (2018) 23:617–22. 10.3851/IMP3243 29923831

[265] Lin Q Zhu L Ni Z Meng H You L Duration of serum neutralizing antibodies for SARS-CoV-2: Lessons from SARS-CoV infection. J Microbiol Immunol Infect (2020) 53(5):821–2. 10.1016/j.jmii.2020.03.015 PMC7141458 32249185

[266] Zhou G Zhao Q Perspectives on therapeutic neutralizing antibodies against the Novel Coronavirus SARS-CoV-2. Int J Biol Sci (2020) 16:1718–23. 10.7150/ijbs.45123 PMC7098029 32226289

[267] Gunn BM Yu W-H Karim MM Brannan JM Herbert AS Wec AZ A Role for Fc Function in Therapeutic Monoclonal Antibody-Mediated Protection against Ebola Virus. Cell Host Microbe (2018) 24:221–33.e5. 10.1016/j.chom.2018.07.009 PMC6298217 30092199

[268] Sharun K Tiwari R Iqbal Yatoo M Patel SK Natesan S Dhama J Antibody-based immunotherapeutics and use of convalescent plasma to counter COVID-19: advances and prospects. Expert Opin Biol Ther (2020) 20:1033–46. 10.1080/14712598.2020.1796963 32744917

[269] World Health Organization Maintaining a safe and adequate blood supply during the pandemic outbreak of coronavirus disease (COVID-19) . Available at: https://www.who.int/publications-detail/maintaining-a-safe-and-adequate-blood-supply-during-the-pandemic-outbreak-of-coronavirus-disease-(covid-19) (Accessed April 28, 2020).

[270] World Health Organization Use of Convalescent Whole Blood or Plasma Collected from Patients Recovered from Ebola Virus Disease for Transfusion, as an Empirical Treatment during Outbreaks. World Health Organization (2014). Available at: WHO/HIS/SDS/2014.8 (Accessed April 28, 2020).

[271] Kumar GV Jeyanthi V Ramakrishnan S A short review on antibody therapy for COVID-19. New Microbes New Infect (2020) 35:100682. 10.1016/j.nmni.2020.100682 32313660 PMC7167584

[272] Wu R Wang L Kuo HCD Shannar A Peter R Chou PJ An Update on Current Therapeutic Drugs Treating COVID-19. Curr Pharmacol Rep (2020) 6:56–70. 10.1007/s40495-020-00216-7 PMC7211915 32395418

[273] Uddin M Mustafa F Rizvi TA Loney T Al Suwaidi H Al-Marzouqi AHH SARS-CoV-2/COVID-19: Viral Genomics, Epidemiology, Vaccines, and Therapeutic Interventions. Viruses (2020) 12(5):526. 10.3390/v12050526 PMC7290442 32397688

[274] Li L Li L Zhang W Zhang W Hu Y Tong X Effect of Convalescent Plasma Therapy on Time to Clinical Improvement in Patients with Severe and Life-threatening COVID-19: A Randomized Clinical Trial. JAMA J Am Med Assoc (2020) 324(5):460–70. 10.1001/jama.2020.10044 PMC7270883 32492084

[275] Tobaiqy M Qashqary M Al-Dahery S Mujallad A Hershan AA Kamal MA Therapeutic management of patients with COVID-19: a systematic review. Infect Prev Pract (2020) 2:100061. 10.1016/j.infpip.2020.100061 PMC7162768 34316558

[276] Keam S Megawati D Patel SK Tiwari R Dhama K Harapan H Immunopathology and immunotherapeutic strategies in severe acute respiratory syndrome coronavirus 2 infection. Rev Med Virol (2020) 30 (5):e2123. 10.1002/rmv.2123 32648313 PMC7404843

[277] Felsenstein S Herbert JA McNamara PS Hedrich CM COVID-19: Immunology and treatment options. Clin Immunol (2020) 215:108448. 10.1016/j.clim.2020.108448 32353634 PMC7185015

[278] De Alwis R Chen S Gan ES Ooi EE Impact of immune enhancement on Covid-19 polyclonal hyperimmune globulin therapy and vaccine development-NC-ND license. (http://creativecommons.org/licenses/by-nc-nd/4.0/). EBioMedicine (2020) 55:102768. 10.1016/j.ebiom.2020.102768 32344202 PMC7161485

[279] Díez JM Romero C Vergara-Alert J Belló-Perez M Rodon J Honrubia JM Cross-neutralization activity against SARS-CoV-2 is present in currently available intravenous immunoglobulins. Immunotherapy (2020) imt–2020-0220. 10.2217/imt-2020-0220 PMC7480323 32900263

[280] Catalan-Dibene J Human antibodies can neutralize SARS-CoV-2. Nat Rev Immunol (2020) 20:1–1. 10.1038/s41577-020-0313-6 PMC7186926 32286538

[281] Ou X Liu Y Lei X Li P Mi D Ren L Characterization of spike glycoprotein of SARS-CoV-2 on virus entry and its immune cross-reactivity with SARS-CoV. Nat Commun (2020) 11:1–12. 10.1038/s41467-020-15562-9 32221306 PMC7100515

[282] Market M Angka L Martel AB Bastin D Olanubi O Tennakoon G Flattening the COVID-19 Curve With Natural Killer Cell Based Immunotherapies. Front Immunol (2020) 11:1512. 10.3389/fimmu.2020.01512 32655581 PMC7324763

[283] Natural Killer Cell (CYNK-001) Infusions in Adults With COVID-19 (CYNK-001-COVID-19) - Full Text View - ClinicalTrials.gov . Available at: https://clinicaltrials.gov/ct2/show/NCT04365101?term=NK%2C+natural+killer&cond=Covid19&draw=2&rank=1 (Accessed September 10, 2020).

[284] A Phase I/II Study of Universal Off-the-shelf NKG2D-ACE2 CAR-NK Cells for Therapy of COVID-19 - Full Text View - ClinicalTrials.gov . Available at: https://clinicaltrials.gov/ct2/show/NCT04324996?term=cell+therapy&cond=Covid19&draw=2 (Accessed September 10, 2020).

[285] Bluestone JA Buckner JH Fitch M Gitelman SE Gupta S Hellerstein MK Type 1 diabetes immunotherapy using polyclonal regulatory T cells. Sci Transl Med (2015) 7(315):315ra189. 10.1126/scitranslmed.aad4134 PMC4729454 26606968

[286] Stephen-Victor E Das M Karnam A Pitard B Gautier J-F Bayry J Potential of regulatory T-cell-based therapies in the management of severe COVID-19. Eur Respir J (2020) 56(3):2002182. 10.1183/13993003.02182-2020 32616599 PMC7331657

[287] Gladstone DE Kim BS Mooney K Karaba AH D’Alessio FR Regulatory T Cells for Treating Patients With COVID-19 and Acute Respiratory Distress Syndrome: Two Case Reports. Ann Intern Med (2020). 10.7326/l20-0681 PMC7370819 32628535

[288] REgulatory T Cell infuSion fOr Lung Injury Due to COVID-19 PnEumonia - Full Text View - ClinicalTrials.gov . Available at: https://clinicaltrials.gov/ct2/show/NCT04468971?cond=treg+covid-19&draw=2&rank=2 (Accessed September 10, 2020).

[289] RAPA-501-Allo Off-the-Shelf Therapy of COVID-19 - Full Text View - ClinicalTrials.gov . Available at: https://clinicaltrials.gov/ct2/show/study/NCT04482699 (Accessed September 10, 2020).

[290] Anti-SARS Cov-2 T Cell Infusions for COVID 19 - Full Text View - ClinicalTrials.gov . Available at: https://clinicaltrials.gov/ct2/show/NCT04401410?term=NCT04401410&draw=2&rank=1 (Accessed September 14, 2020).

[291] Part Two of Novel Adoptive Cellular Therapy With SARS-CoV-2 Specific T Cells in Patients With Severe COVID-19 - Full Text View - ClinicalTrials.gov . Available at: https://clinicaltrials.gov/ct2/show/NCT04457726?term=NCT04457726&draw=2&rank=1 (Accessed September 14, 2020).

[292] Novel Adoptive Cellular Therapy With SARS-CoV-2 Specific T Cells in Patients With Severe COVID-19 - Full Text View - ClinicalTrials.gov . Available at: https://clinicaltrials.gov/ct2/show/NCT04351659?term=NCT04351659&draw=1&rank=1 (Accessed September 14, 2020).

[293] Papadopoulos EB Ladanyi M Emanuel D Mackinnon S Boulad F Carabasi MH Infusions of donor leukocytes to treat epstein-barr virus-associated lymphoproliferative disorders after allogeneic bone marrow transplantation. N Engl J Med (1994) 330:1185–91. 10.1056/NEJM199404283301703 8093146

[294] Tzannou I Papadopoulou A Naik S Leung K Martinez CA Ramos CA Off-the-shelf virus-specific T cells to treat BK virus, human herpesvirus 6, cytomegalovirus, Epstein-Barr virus, and adenovirus infections after allogeneic hematopoietic stem-cell transplantation. J Clin Oncol (2017) 35:3547–57. 10.1200/JCO.2017.73.0655 PMC5662844 28783452

[295] Rooney CM Ng CYC Loftin S Smith CA Li C Krance RA Use of gene-modified virus-specific T lymphocytes to control Epstein-Barr-virus-related lymphoproliferation. Lancet (1995) 345:9–13. 10.1016/S0140-6736(95)91150-2 7799740

[296] Prockop S Doubrovina E Suser S Heller G Barker J Dahi P Off-the-shelf EBV-specific T cell immunotherapy for rituximab-refractory EBV-associated lymphoma following transplantation. J Clin Invest (2020) 130:733–47. 10.1172/JCI121127 PMC6994129 31689242

[297] Hanley B Roufosse CA Osborn M Naresh KN Convalescent donor SARS-COV-2-specific cytotoxic T lymphocyte infusion as a possible treatment option for COVID-19 patients with severe disease has not received enough attention till date. Br J Haematol (2020) 189:1062–3. 10.1111/bjh.16780 32369628

[298] Barata JT Durum SK Seddon B Flip the coin: IL-7 and IL-7R in health and disease. Nat Immunol (2019) 20:1584–93. 10.1038/s41590-019-0479-x 31745336

[299] MacKall CL Fry TJ Gress RE Harnessing the biology of IL-7 for therapeutic application. Nat Rev Immunol (2011) 11:330–42. 10.1038/nri2970 PMC7351348 21508983

[300] Francois B Jeannet R Daix T Walton AH Shotwell MS Unsinger J Interleukin-7 restores lymphocytes in septic shock: the IRIS-7 randomized clinical trial. JCI Insight (2018) 3. 10.1172/jci.insight.98960 PMC5922293 29515037

[301] Monneret G de Marignan D Coudereau R Bernet C Ader F Frobert E Immune monitoring of interleukin-7 compassionate use in a critically ill COVID-19 patient. Cell Mol Immunol (2020) 17. 10.1038/s41423-020-0516-6 PMC7387803 32728202

[302] Laterre PF François B Collienne C Hantson P Jeannet R Remy KE Association of Interleukin 7 Immunotherapy With Lymphocyte Counts Among Patients With Severe Coronavirus Disease 2019 (COVID-19). JAMA Netw Open (2020) 3:e2016485. 10.1001/jamanetworkopen.2020.16485 32697322 PMC7376391

[303] Zhang L Liu Y Potential interventions for novel coronavirus in China: A systematic review. J Med Virol (2020) 92:479–90. 10.1002/jmv.25707 PMC7166986 32052466

[304] Chu CM Cheng CC Hung FN Wong MML Chan H Chan S Role of lopinavir/ritonavir in the treatment of SARS: initial virological and clinical findings. Thorax (2004) 59:252–6. 10.1136/thorax.2003.012658 PMC1746980 14985565

[305] Choy K-T Yin-Lam Wong A Kaewpreedee P Sia S-F Chen D Yan Hui KP Remdesivir, lopinavir, emetine, and homoharringtonine inhibit SARS-CoV-2 replication in vitro. Antiviral Res (2020) 178:104786. 10.1016/j.antiviral.2020.104786 32251767 PMC7127386

[306] Kang CK Seong M-W Choi S-J Kim TS Choe PG Song SH *In vitro* activity of lopinavir/ritonavir and hydroxychloroquine against severe acute respiratory syndrome coronavirus 2 at concentrations achievable by usual doses. Korean J Intern Med (2020) 35(4):728–87. 10.3904/kjim.2020.157 PMC7373950 32460458

[307] Lim J Jeon S Shin HY Kim MJ Seong YM Lee WJ Case of the index patient who caused tertiary transmission of coronavirus disease 2019 in Korea: The application of lopinavir/ritonavir for the treatment of COVID-19 pneumonia monitored by quantitative RT-PCR. J Korean Med Sci (2020) 35. 10.3346/jkms.2020.35.e79 32080993

[308] Han W Quan B Guo Y Zhang J Lu Y Feng G The course of clinical diagnosis and treatment of a case infected with coronavirus disease 2019. J Med Virol (2020) 92:461–3. 10.1002/jmv.25711 PMC7167012 32073161

[309] Kim JY Choe PG Oh Y Oh KJ Kim J Park SJ The first case of 2019 novel coronavirus pneumonia imported into korea from wuhan, china: Implication for infection prevention and control measures. J Korean Med Sci (2020) 35. 10.3346/jkms.2020.35.e61 PMC7008073 32030925

[310] Wang Z Chen X Lu Y Chen F Zhang W Clinical characteristics and therapeutic procedure for four cases with 2019 novel coronavirus pneumonia receiving combined Chinese and Western medicine treatment. Biosci Trends (2020) 14. 10.5582/BST.2020.01030 32037389

[311] Liu F Xu A Zhang Y Xuan W Yan T Pan K Patients of COVID-19 may benefit from sustained lopinavir-combined regimen and the increase of eosinophil may predict the outcome of COVID-19 progression. Int J Infect Dis (2020) 95:P183–91. 10.1016/j.ijid.2020.03.013 PMC7193136 32173576

[312] Deng L Li C Zeng Q Liu X Li X Zhang H Arbidol combined with LPV/r versus LPV/r alone against Corona Virus Disease 2019: A retrospective cohort study. J Infect (2020) 81(1):p1–5. 10.1016/j.jinf.2020.03.002 PMC7156152 32171872

[313] Cao B Wang Y Wen D Liu W Wang J Fan G A Trial of Lopinavir–Ritonavir in Adults Hospitalized with Severe Covid-19. N Engl J Med (2020) 382:NEJMoa2001282. 10.1056/NEJMoa2001282 PMC7121492 32187464

[314] Villalaín J Membranotropic effects of arbidol, a broad anti-viral molecule, on phospholipid model membranes. J Phys Chem B (2010) 114:8544–54. 10.1021/jp102619w 20527735

[315] Vankadari N Arbidol: A potential antiviral drug for the treatment of SARS-CoV-2 by blocking trimerization of the spike glycoprotein. Int J Antimicrob Agents (2020) 56:105998. 10.1016/j.ijantimicag.2020.105998 32360231 PMC7187825

[316] Chen W Yao M Fang Z Lv X Deng M Wu Z A study on clinical effect of Arbidol combined with adjuvant therapy on COVID-19. J Med Virol (2020) 92:2702–8. 10.1002/jmv.26142 PMC7300876 32510169

[317] Zhu Z Lu Z Xu T Chen C Yang G Zha T Arbidol Monotherapy is Superior to Lopinavir/ritonavir in Treating COVID-19. J Infect (2020) 81(1):E21–3. 10.1016/j.jinf.2020.03.060 PMC7195393 32283143

[318] Khalid M Al Rabiah F Khan B Al Mobeireek A Butt TS Al Mutairy E Ribavirin and interferon-α2b as primary and preventive treatment for Middle East respiratory syndrome coronavirus: A preliminary report of two cases. Antivir Ther (2015) 20:87–91. 10.3851/IMP2792 24831606

[319] Ölschläger S Neyts J Günther S Depletion of GTP pool is not the predominant mechanism by which ribavirin exerts its antiviral effect on Lassa virus. Antiviral Res (2011) 91:89–93. 10.1016/j.antiviral.2011.05.006 21616094

[320] Elfiky AA Anti-HCV, nucleotide inhibitors, repurposing against COVID-19. Life Sci (2020) 248. 10.1016/j.lfs.2020.117477 PMC7089605 32119961

[321] Li X Yang Y Liu L Yang X Zhao X Li Y Effect of combination antiviral therapy on hematological profiles in 151 adults hospitalized with severe coronavirus disease 2019. Pharmacol Res (2020) 160:105036. 10.1016/j.phrs.2020.105036 32565309 PMC7301803

[322] Hung IFN Lung KC Tso EYK Liu R Chung TWH Chu MY Triple combination of interferon beta-1b, lopinavir–ritonavir, and ribavirin in the treatment of patients admitted to hospital with COVID-19: an open-label, randomised, phase 2 trial. Lancet (2020) 395:1695–704. 10.1016/S0140-6736(20)31042-4 PMC7211500 32401715

[323] Yuan J Zou R Zeng L Kou S Lan J Li X The correlation between viral clearance and biochemical outcomes of 94 COVID-19 infected discharged patients. Inflammation Res (2020) 69:1–8. 10.1007/s00011-020-01342-0 PMC7103893 32227274

[324] Khalili JS Zhu H Mak NSA Yan Y Zhu Y Novel coronavirus treatment with ribavirin: Groundwork for an evaluation concerning COVID-19. J Med Virol (2020) 92:740–6. 10.1002/jmv.25798 PMC7228408 32227493

[325] Amirian ES Levy JK Current knowledge about the antivirals remdesivir (GS-5734) and GS-441524 as therapeutic options for coronaviruses. One Health. (2020) 9:100128. 10.1016/j.onehlt.2020.100128 32258351 PMC7118644

[326] Wang M Cao R Zhang L Yang X Liu J Xu M Remdesivir and chloroquine effectively inhibit the recently emerged novel coronavirus (2019-nCoV) in vitro. Cell Res (2020) 30:269–71. 10.1038/s41422-020-0282-0 PMC7054408 32020029

[327] Grein J Ohmagari N Shin D Diaz G Asperges E Castagna A Compassionate Use of Remdesivir for Patients with Severe Covid-19. N Engl J Med (2020) 382:NEJMoa2007016. 10.1056/NEJMoa2007016 PMC7169476 32275812

[328] Beigel JH Tomashek KM Dodd LE Mehta AK Zingman BS Kalil AC Remdesivir for the Treatment of Covid-19 — Preliminary Report. N Engl J Med (2020) 8:NEJMoa2007764. 10.1056/nejmoa2007764 32649078

[329] Antinori S Cossu MV Ridolfo AL Rech R Bonazzetti C Pagani G Compassionate remdesivir treatment of severe Covid-19 pneumonia in intensive care unit (ICU) and Non-ICU patients: Clinical outcome and differences in post-treatment hospitalisation status. Pharmacol Res (2020) 158. 10.1016/j.phrs.2020.104899 PMC7212963 32407959

[330] Goldman JD Lye DCB Hui DS Marks KM Bruno R Montejano R Remdesivir for 5 or 10 Days in Patients with Severe Covid-19. N Engl J Med (2020) 27:NEJMoa2015301. 10.1056/nejmoa2015301 PMC7377062 32459919

[331] Furuta Y Komeno T Nakamura T Favipiravir (T-705), a broad spectrum inhibitor of viral RNA polymerase. Proc Japan Acad Ser B (2017) 93:449–63. 10.2183/pjab.93.027 PMC5713175 28769016

[332] Li G De Clercq E Therapeutic options for the 2019 novel coronavirus (2019-nCoV). NLM (Medline) (2020) 19(3):149–50. 10.1038/d41573-020-00016-0 32127666

[333] Cai Q Yang M Liu D Chen J Shu D Xia J Experimental Treatment with Favipiravir for COVID-19: An Open-Label Control Study. Engineering (Beijing) (2020). 10.1016/j.eng.2020.03.007 PMC7185795 32346491

[334] Sallard E Lescure F-X Yazdanpanah Y Mentre F Peiffer-Smadja N Type 1 interferons as a potential treatment against COVID-19. Antiviral Res (2020) 178:104791. 10.1016/j.antiviral.2020.104791 32275914 PMC7138382

[335] Dong L Hu S Gao J Discovering drugs to treat coronavirus disease 2019 (COVID-19). Drug Discov Ther (2020) 14:58–60. 10.5582/ddt.2020.01012 32147628

[336] Shen KL Yang YH Diagnosis and treatment of 2019 novel coronavirus infection in children: a pressing issue. World J Pediatr (2020) 1–3. 10.1007/s12519-020-00344-6 PMC7091265 32026147

[337] McCreary EK Pogue JM Coronavirus Disease 2019 Treatment: A Review of Early and Emerging Options. Open Forum Infect Dis (2020) 7:ofaa105. 10.1093/ofid/ofaa105 32284951 PMC7144823

[338] Mantlo E Bukreyeva N Maruyama J Paessler S Huang C Antiviral activities of type I interferons to SARS-CoV-2 infection. Antiviral Res (2020) 179:104811. 10.1016/j.antiviral.2020.104811 32360182 PMC7188648

[339] Clementi N Ferrarese R Criscuolo E Diotti RA Castelli M Scagnolari C Interferon-β 1a Inhibits SARS-CoV-2 in Vitro When Administered After Virus Infection - PubMed. J Infect Dis (2020) 222(5):722–5. 10.1093/infdis/jiaa350 PMC7337790 32559285

[340] Zuo Y Liu Y Zhong Q Zhang K Xu Y Wang Z Lopinavir/ritonavir and interferon combination therapy may help shorten the duration of viral shedding in patients with COVID-19: a retrospective study in two designated hospitals in Anhui, China. J Med Virol (2020) 92(11):2666–74. 10.1002/jmv.26127 PMC7300569 32492211

[341] Zhou Q Chen V Shannon CP Wei XS Xiang X Wang X Interferon-α2b Treatment for COVID-19. Front Immunol (2020) 11:1061. 10.3389/fimmu.2020.01061 32574262 PMC7242746

[342] Xu P Huang J Fan Z Huang W Qi M Lin X Arbidol/IFN-α2b therapy for patients with corona virus disease 2019: a retrospective multicenter cohort study. Microbes Infect (2020) 22. 10.1016/j.micinf.2020.05.012 PMC7238991 32445881

[343] Dastan F Nadji SA Saffaei A Marjani M Moniri A Jamaati H Subcutaneous administration of interferon beta-1a for COVID-19: A non-controlled prospective trial. Int Immunopharmacol (2020) 85. 10.1016/j.intimp.2020.106688 PMC7275997 32544867

[344] Wang N Zhan Y Zhu L Hou Z Liu F Song P Retrospective Multicenter Cohort Study Shows Early Interferon Therapy Is Associated with Favorable Clinical Responses in COVID-19 Patients. Cell Host Microbe (2020) 28:455–464.e2. 10.1016/j.chom.2020.07.005 PMC7368656 32707096

[345] Davoudi-Monfared E Rahmani H Khalili H Hajiabdolbaghi M Salehi M Abbasian L A Randomized Clinical Trial of the Efficacy and Safety of Interferon β-1a in Treatment of Severe COVID-19. Antimicrob Agents Chemother (2020) 64. 10.1128/aac.01061-20 PMC7449227 32661006

[346] World Health Organization WHO Model Lists of Essential Medicines. (Geneva: World Health Organization) (2020).

[347] Schrezenmeier E Dörner T Mechanisms of action of hydroxychloroquine and chloroquine: implications for rheumatology. Nat Rev Rheumatol (2020) 16:155–66. 10.1038/s41584-020-0372-x 32034323

[348] Devaux CA Rolain J-M Colson P Raoult D New insights on the antiviral effects of chloroquine against coronavirus: what to expect for COVID-19? Int J Antimicrob Agents (2020) 55:105938. 10.1016/j.ijantimicag.2020.105938 32171740 PMC7118659

[349] Savarino A Boelaert JR Cassone A Majori G Cauda R Effects of chloroquine on viral infections: An old drug against today’s diseases? Lancet Infect Dis (2003) 3:722–7. 10.1016/S1473-3099(03)00806-5 PMC7128816 14592603

[350] McChesney EW Animal toxicity and pharmacokinetics of hydroxychloroquine sulfate. Am J Med (1983) 75:11–8. 10.1016/0002-9343(83)91265-2 6408923

[351] Vincent MJ Bergeron E Benjannet S Erickson BR Rollin PE Ksiazek TG Chloroquine is a potent inhibitor of SARS coronavirus infection and spread. Virol J (2005) 2. 10.1186/1743-422X-2-69 PMC1232869 16115318

[352] Mauthe M Orhon I Rocchi C Zhou X Luhr M Hijlkema KJ Chloroquine inhibits autophagic flux by decreasing autophagosome-lysosome fusion. Autophagy (2018) 14:1435–55. 10.1080/15548627.2018.1474314 PMC6103682 29940786

[353] Savarino A Di Trani L Donatelli I Cauda R Cassone A New insights into the antiviral effects of chloroquine. Lancet Infect Dis (2006) 6:67–9. 10.1016/S1473-3099(06)70361-9 PMC7129107 16439323

[354] Wallace DJ Linker-Israeli M Hyun S Klinenberg JR Stecher V The effect of hydroxychloroquine therapy on serum levels of immunoregulatory molecules in patients with systemic lupus erythematosus [6]. J Rheumatol (1994) 21:375–6. 8182661

[355] Klinefelter HF Achurra A Effect of gold salts and antimalarials on the rheumatoid factor in rheumatoid arthritis. Scand J Rheumatol (1973) 2:177–82. 10.3109/03009747309097086 4203561

[356] Dixon JS Pickup ME Bird HA Lee MR Wright V Downie WW Biochemical indices of response to hydroxychloroquine and sodium aurothiomalate in rheumatoid arthritis. Ann Rheum Dis (1981) 40:480–8. 10.1136/ard.40.5.480 PMC1000785 6796009

[357] Zhou D Dai SM Tong Q COVID-19: a recommendation to examine the effect of hydroxychloroquine in preventing infection and progression. J Antimicrob Chemother (2020) 75(7):1667–70. 10.1093/jac/dkaa114 PMC7184499 32196083

[358] Accapezzato D Visco V Francavilla V Molette C Donato T Paroli M Chloroquine enhances human CD8+ T cell responses against soluble antigens in vivo. J Exp Med (2005) 202:817–28. 10.1084/jem.20051106 PMC2212941 16157687

[359] Wallace DJ Linker-Israeli M Metzger AL Stecher VJ The Relevance of Antimalarial Therapy with Regard to Thrombosis, Hypercholesterolemia and Cytokines in SLE. Lupus (1993) 2:13–5. 10.1177/0961203393002001041 8485565

[360] Liu J Cao R Xu M Wang X Zhang H Hu H Hydroxychloroquine, a less toxic derivative of chloroquine, is effective in inhibiting SARS-CoV-2 infection in vitro. Cell Discov (2020) 6:16. 10.1038/s41421-020-0156-0 32194981 PMC7078228

[361] Yao X Ye F Zhang M Cui C Huang B Niu P In Vitro Antiviral Activity and Projection of Optimized Dosing Design of Hydroxychloroquine for the Treatment of Severe Acute Respiratory Syndrome Coronavirus 2 (SARS-CoV-2). Clin Infect Dis (2020) 71(15):732–9. 10.1093/cid/ciaa237 PMC7108130 32150618

[362] Andreani J Le Bideau M Duflot I Jardot P Rolland C Boxberger M In vitro testing of combined hydroxychloroquine and azithromycin on SARS-CoV-2 shows synergistic effect. Microb Pathog (2020) 145. 10.1016/j.micpath.2020.104228 PMC7182748 32344177

[363] Gao J Tian Z Yang X Breakthrough: Chloroquine phosphate has shown apparent efficacy in treatment of COVID-19 associated pneumonia in clinical studies. Biosci Trends (2020) 14. 10.5582/BST.2020.01047 32074550

[364] Damle B Vourvahis M Wang E Leaney J Corrigan B Clinical Pharmacology Perspectives on the Antiviral Activity of Azithromycin and Use in COVID-19. Clin Pharmacol Ther (2020) 108:cpt.1857. 10.1002/cpt.1857 PMC7262099 32302411

[365] Gautret P Lagier J-C Parola P Hoang VT Meddeb L Mailhe M Hydroxychloroquine and azithromycin as a treatment of COVID-19: results of an open-label non-randomized clinical trial. Int J Antimicrob Agents (2020) 56:105949. 10.1016/j.ijantimicag.2020.105949 32205204 PMC7102549

[366] Gautret P Lagier J-C Parola P Hoang VT Meddeb L Sevestre J Clinical and microbiological effect of a combination of hydroxychloroquine and azithromycin in 80 COVID-19 patients with at least a six-day follow up: A pilot observational study. Travel Med Infect Dis (2020) 34:101663. 10.1016/j.tmaid.2020.101663 32289548 PMC7151271

[367] Arshad S Kilgore P Chaudhry ZS Jacobsen G Wang DD Huitsing K Treatment with hydroxychloroquine, azithromycin, and combination in patients hospitalized with COVID-19. Int J Infect Dis (2020) 97:396–403. 10.1016/j.ijid.2020.06.099 32623082 PMC7330574

[368] Lee TC MacKenzie LJ McDonald EG Tong SYC An observational cohort study of hydroxychloroquine and azithromycin for COVID-19: (Can’t Get No) Satisfaction. Int J Infect Dis (2020) 98:216–7. 10.1016/j.ijid.2020.06.095 PMC7331530 32623080

[369] Varisco TJ Johnson ML Thornton D Comment on Arshad et al: Treatment with Hydroxychloroquine, Azithromycin, and Combination in Patients Hospitalized with COVID-19. Int J Infect Dis (2020) 99:373. 10.1016/j.ijid.2020.07.071 32771630 PMC7409789

[370] Atkinson JG Problems with the analysis in “Treatment with Hydroxychloroquine, Azithromycin, and Combination in Patients Hospitalized with COVID-19.” Int J Infect Dis (2020) 99:37. 10.1016/j.ijid.2020.07.057 32738492 PMC7390751

[371] Rosenberg ES Holtgrave DR Udo T Clarifying the record on hydroxychloroquine for the treatment of patients hospitalized with COVID-19. Int J Infect Dis (2020) 99:38–9. 10.1016/j.ijid.2020.07.055 PMC7388815 32738483

[372] Malviya A The continued dilemma about usage of Hydroxychloroquine: Respite is in randomized control trials. Int J Infect Dis (2020) 99:310–1. 10.1016/j.ijid.2020.07.054 PMC7388893 32738490

[373] Brito-Azevedo A Hydroxychloroquine in COVID-19: taking care of statistic to take care of patients. Int J Infect Dis (2020) 99:324. 10.1016/j.ijid.2020.07.079 32768698 PMC7406426

[374] Million M Lagier JC Gautret P Colson P Fournier PE Amrane S Early treatment of COVID-19 patients with hydroxychloroquine and azithromycin: A retrospective analysis of 1061 cases in Marseille, France. Travel Med Infect Dis (2020) 35:101738. 10.1016/j.tmaid.2020.101738 32387409 PMC7199729

[375] Boulware DR Pullen MF Bangdiwala AS Pastick KA Lofgren SM Okafor EC A Randomized Trial of Hydroxychloroquine as Postexposure Prophylaxis for Covid-19. N Engl J Med (2020) 383:517–25. 10.1056/nejmoa2016638 PMC7289276 32492293

[376] Huang M Tang T Pang P Li M Ma R Lu J Treating COVID-19 with Chloroquine. J Mol Cell Biol (2020) 12:322. 10.1093/JMCB/MJAA014 32236562 PMC7232130

[377] Satlin MJ Goyal P Magleby R Maldarelli GA Pham K Kondo M Safety, tolerability, and clinical outcomes of hydroxychloroquine for hospitalized patients with coronavirus 2019 disease. PLoS One (2020) 15. 10.1371/journal.pone.0236778 PMC7377460 32701969

[378] Magagnoli J Narendran S Pereira F Cummings TH Hardin JW Sutton SS Outcomes of Hydroxychloroquine Usage in United States Veterans Hospitalized with COVID-19. Med (2020) 1. 10.1016/j.medj.2020.06.001 PMC7274588 32838355

[379] Rosenberg ES Dufort EM Udo T Wilberschied LA Kumar J Tesoriero J Association of Treatment with Hydroxychloroquine or Azithromycin with In-Hospital Mortality in Patients with COVID-19 in New York State. JAMA J Am Med Assoc (2020) 323:2493–502. 10.1001/jama.2020.8630 PMC7215635 32392282

[380] Ip A Berry DA Hansen E Goy AH Pecora AL Sinclaire BA Hydroxychloroquine and tocilizumab therapy in COVID-19 patients-An observational study. PLoS One (2020) 15:e0237693. 10.1371/journal.pone.0237693 32790733 PMC7425928

[381] Mahévas M Tran VT Roumier M Chabrol A Paule R Guillaud C Clinical efficacy of hydroxychloroquine in patients with covid-19 pneumonia who require oxygen: Observational comparative study using routine care data. BMJ (2020) 369:369. 10.1136/bmj.m1844 PMC7221472 32409486

[382] Tang W Cao Z Han M Wang Z Chen J Sun W Hydroxychloroquine in patients with mainly mild to moderate coronavirus disease 2019: Open label, randomised controlled trial. BMJ (2020) 369. 10.1136/bmj.m1849 PMC7221473 32409561

[383] Borba MGS Val FFA Sampaio VS Alexandre MAA Melo GC Brito M Effect of High vs Low Doses of Chloroquine Diphosphate as Adjunctive Therapy for Patients Hospitalized With Severe Acute Respiratory Syndrome Coronavirus 2 (SARS-CoV-2) Infection: A Randomized Clinical Trial. JAMA Netw Open (2020) 3:e208857. 10.1001/jamanetworkopen.2020.8857 32330277 PMC12124691

[384] Saleh M Gabriels J Chang D Kim BS Mansoor A Mahmood E The Effect of Chloroquine, Hydroxychloroquine and Azithromycin on the Corrected QT Interval in Patients with SARS-CoV-2 Infection. Circ Arrhythm Electrophysiol (2020) 13. 10.1161/CIRCEP.120.008662 PMC7299095 32347743

[385] Geleris J Sun Y Platt J Zucker J Baldwin M Hripcsak G Observational Study of Hydroxychloroquine in Hospitalized Patients with Covid-19. N Engl J Med (2020) 382:2411–8. 10.1056/nejmoa2012410 PMC7224609 32379955

[386] Xu J Shi P-Y Li H Zhou J Broad Spectrum Antiviral Agent Niclosamide and Its Therapeutic Potential. ACS Infect Dis (2020) 6(5):909–15. 10.1021/acsinfecdis.0c00052 PMC7098069 32125140

[387] Caly L Druce JD Catton MG Jans DA Wagstaff KM The FDA-approved Drug Ivermectin inhibits the replication of SARS-CoV-2 in vitro. Antiviral Res (2020) 178:104787. 10.1016/j.antiviral.2020.104787 32251768 PMC7129059

[388] Wagstaff KM Sivakumaran H Heaton SM Harrich D Jans DA Ivermectin is a specific inhibitor of importin α/β-mediated nuclear import able to inhibit replication of HIV-1 and dengue virus. Biochem J (2012) 443:851–6. 10.1042/BJ20120150 PMC3327999 22417684

[389] Lehrer S Rheinstein PH Ivermectin Docks to the SARS-CoV-2 Spike Receptor-binding Domain Attached to ACE2. Vivo (Brooklyn) (2020) 34:3023–6. 10.21873/invivo.12134 PMC7652439 32871846

[390] Guzzo CA Furtek CI Porras AG Chen C Tipping R Clineschmidt CM Safety, tolerability, and pharmacokinetics of escalating high doses of ivermectin in healthy adult subjects. J Clin Pharmacol (2002) 42:1122–33. 10.1177/009127002401382731 12362927

[391] Navarro M Camprubí D Requena-Méndez A Buonfrate D Giorli G Kamgno J Safety of high-dose ivermectin: A systematic review and meta-analysis. J Antimicrob Chemother (2020) 75:827–34. 10.1093/jac/dkz524 31960060

[392] Duthaler U Suenderhauf C Karlsson MO Hussner J Meyer zu Schwabedissen H Krähenbühl S Population pharmacokinetics of oral ivermectin in venous plasma and dried blood spots in healthy volunteers. Br J Clin Pharmacol (2019) 85:626–33. 10.1111/bcp.13840 PMC6379217 30566757

[393] Rossignol JF Nitazoxanide, a new drug candidate for the treatment of Middle East respiratory syndrome coronavirus. J Infect Public Health (2016) 9:227–30. 10.1016/j.jiph.2016.04.001 PMC7102735 27095301

[394] Yavuz S Ünal S Antiviral Treatment of COVID-19. Turkish J Med Sci (2020) 50. 10.3906/sag-2004-145 PMC7195979 32293834

[395] Tang N Bai H Chen X Gong J Li D Sun Z Anticoagulant treatment is associated with decreased mortality in severe coronavirus disease 2019 patients with coagulopathy. J Thromb Haemost (2020) 18:1094–9. 10.1111/jth.14817 PMC9906401 32220112

[396] Iba T Arakawa M Di Nisio M Gando S Anan H Sato K Newly Proposed Sepsis-Induced Coagulopathy Precedes International Society on Thrombosis and Haemostasis Overt-Disseminated Intravascular Coagulation and Predicts High Mortality. J Intens Care Med (2020) 35:643–9. 10.1177/0885066618773679 29720054

[397] Lillicrap D Disseminated intravascular coagulation in patients with 2019-nCoV pneumonia. J Thromb Haemost (2020) 18:786–7. 10.1111/jth.14781 PMC7166410 32212240

[398] Paranjpe I Fuster V Lala A Russak A Glicksberg BS Levin MA Association of Treatment Dose Anticoagulation with In-Hospital Survival Among Hospitalized Patients with COVID-19. J Am Coll Cardiol (2020) 76 (1):122–4. 10.1016/j.jacc.2020.05.001 PMC7202841 32387623

[399] Thachil J The versatile heparin in COVID-19. J Thromb Haemost (2020) 18. 10.1111/jth.14821 PMC9906146 32239799

[400] Liu J Li J Arnold K Pawlinski R Key NS Using heparin molecules to manage COVID-2019. Res Pract Thromb Haemost (2020) 4:518–23. 10.1002/rth2.12353 PMC7264589 32542212

[401] Lindahl U Li JP Heparin – an old drug with multiple potential targets in Covid-19 therapy. J Thromb Haemost (2020) 18(9):2422–4. 10.1111/jth.14898 PMC7276884 32426897

[402] Patel SK Saikumar G Rana J Dhama J Yatoo MI Tiwari R Dexamethasone: A boon for critically ill COVID-19 patients? Travel Med Infect Dis (2020) 37:101844. 10.1016/j.tmaid.2020.101844 32791213 PMC7416109

[403] Sharun K Tiwari R Dhama J Dhama K Dexamethasone to combat cytokine storm in COVID-19: Clinical trials and preliminary evidence. Int J Surg (2020) 82:179–81. 10.1016/j.ijsu.2020.08.038 PMC7472975 32896649

[404] RECOVERY Collaborative Group Horby P Lim WS Emberson JR Mafham M Bell JL Dexamethasone in Hospitalized Patients with Covid-19 — Preliminary Report. N Engl J Med (2020) NEJMoa2021436. 10.1056/nejmoa2021436 PMC7383595 32678530

[405] Tomazini BM Maia IS Cavalcanti AB Berwanger O Rosa RG Veiga VC Effect of Dexamethasone on Days Alive and Ventilator-Free in Patients With Moderate or Severe Acute Respiratory Distress Syndrome and COVID-19: The CoDEX Randomized Clinical Trial. JAMA (2020) 324(13):1307–16. 10.1001/jama.2020.17021 PMC7489411 32876695

[406] Villar J Ferrando C Martínez D Ambrós A Muñoz T Soler JA Dexamethasone treatment for the acute respiratory distress syndrome: a multicentre, randomised controlled trial. Lancet Respir Med (2020) 8:267–76. 10.1016/S2213-2600(19)30417-5 32043986

